# Longitudinal evaluation of cognition after stroke – A systematic scoping review

**DOI:** 10.1371/journal.pone.0221735

**Published:** 2019-08-29

**Authors:** Juan Pablo Saa, Tamara Tse, Carolyn Baum, Toby Cumming, Naomi Josman, Miranda Rose, Leeanne Carey

**Affiliations:** 1 Occupational Therapy, School of Allied Health, Human Services and Sport, College of Science Health and Engineering, La Trobe University, Melbourne, Australia; 2 Neurorehabilitation and Recovery, Florey Institute of Neuroscience and Mental Health, University of Melbourne, Melbourne, Australia; 3 Stroke Division, Florey Institute of Neuroscience and Mental Health, University of Melbourne, Melbourne, Australia; 4 Occupational Therapy, Washington University School of Medicine, Saint Louis, Missouri, United States of America; 5 George Warren Brown School of Social Work, Washington University in Saint Louis, Saint Louis, Missouri, United States of America; 6 Department of Occupational Therapy, University of Haifa, Haifa, Israel; 7 Speech Pathology, School of Allied Health, Human Services and Sport, College of Science Health and Engineering, La Trobe University, Melbourne, Australia; University of Glasgow, UNITED KINGDOM

## Abstract

**Background:**

Cognitive impairment affects up to 80 percent of the stroke population, however, both the available evidence about post-stroke cognition and the measures used to evaluate it longitudinally have not been well described. The aims of this systematic scoping review were: to identify and characterize studies evaluating cognition longitudinally after stroke; to summarize the cognitive instruments used and the domains they target; and to organize cognitive domains assessed using the International Classification of Functioning, Disability and Health (ICF).

**Methods:**

We used a systematic scoping approach to search for peer-reviewed articles involving adults with stroke that evaluated cognition longitudinally. Screening of titles, abstracts, and full reports was completed independently by two reviewers, across six electronic databases (PubMed, PsycInfo, Medline, Cinahl Plus, Embase, and Web of Science). Cognitive domains were mapped to an ICF function independently by the same two reviewers, using a previously tested, standardized approach.

**Results:**

A total of 5,540 records were found; 257 were included, representing a total pooled sample of 120,860 stroke survivors. Of these studies, 200 (78%) provided specific cognitive outcomes from the longitudinal evaluations, 57 (22%) reported model predictions, and 77 (30%) included interventions. Cognition was evaluated with 356 unique instruments, targeting 95 distinct cognitive domains, and 17 mental functions from the ICF. The Mini-Mental State Examination was the most frequently used instrument (117 reports, 46%). Other tools used longitudinally were the Trail Making Test (17% of reports), tests of verbal fluency (14%), the Functional Independence Measure (14%), the Montreal Cognitive Assessment (13%), the Digit Span (11%), and the Stroop test (10%). Global cognition was evaluated in 170 reports (66%), followed by higher-level cognitive functioning (29%), memory (28%), language (21%), attention (21%), and perceptual skills (14%). Studies using functional (or performance-based) cognitive assessments over time were scarce (< 1%).

**Conclusion:**

Our findings indicate that whilst there is a substantial number of studies available that report longitudinal evaluations of cognition after stroke, there is large variability in the measures used and the cognitive domains they target. Nonetheless, the available data for evaluation of cognition over time after stroke can be organized and described systematically.

## Background

Impairment in cognitive function is one of the most common issues affecting stroke patients [1]. It is estimated that up to 80 percent of the stroke population experience some level of cognitive impairment during their recovery [2,3]. Cognitive skills play a crucial role in the successful execution of most daily life activities, hence its accurate evaluation is a vital aspect of stroke rehabilitation and recovery [4].

Despite the high prevalence of cognitive impairment amongst the stroke population, a relative lack of evidence appears to exist about its assessment over time [5,6]. Large-scale longitudinal studies are limited [7,8], with very few reports describing cognitive outcomes five years or more after symptom onset [8–10]. Additionally, these studies often report the incidence of cognitive impairment, without quantifying overall decline. Published and ongoing systematic reviews [5,11] have adopted the same approach, reporting rates of impaired individuals over time, as opposed to improvement or decline in a continuous scale. These studies have for the most part been unsuccessful at gathering detailed data to describe a trajectory of cognitive function using meta-analytic methods.

A second issue in describing post stroke cognition is the great variability found in survivors’ cognitive profiles [12]. This issue can be attributed to the inherent differences seen in individual patients, and also to the well-known inconsistencies in the instruments used to assess cognition [13]. For instance, recent epidemiological evidence [14] indicated that the proportion of cognitively impaired individuals in studies with similar baseline characteristics [15,16] varied from 24 to 96 percent, 1–3 months post stroke onset, when the evaluation administered at follow-up was a screening tool, versus a more comprehensive cognitive battery. Alongside these inconsistencies, the trajectory of cognitive function can be biased due to reasons that include, but are not limited to, test insensitivity [17,18], patient dropout [19], and inaccurate representation of common comorbidities like aphasia or dementia [16,17,20–22].

Last, but not least, an important group of limitations is the scarce use of evaluation tools that directly assess the impact of cognition in the context of functional activities. Interestingly, activity performance is hardly addressed in stroke cognitive evaluation, despite being a clear priority for survivors [23]. Common areas affected by cognitive impairment documented in the literature include work [24–26], driving [27,28], leisure [29], physical activity [30], and community and social participation [31,32]. Addressing these limitations is an ongoing challenge in stroke rehabilitation, particularly for stroke survivors who are young and/or viewed as having mild or moderate stroke severity; which represent more than half the stroke population when taken as a subgroup [23,33].

In short, the characterization of cognitive profiles and their association with functional recovery is a manifest and extremely challenging task in current research and clinical practice that needs thorough review and organization. The literature is limited in this area, particularly when looking at the assessment of cognition beyond the impairment level [34]. Assessment of cognition is usually accompanied by global disability or functional activity measures. Yet, these two areas are typically assessed separately, with focus on identifying impairment or reduced independence, rather than quantifying cognitive impairment in functional tasks with specified cognitive demands.

In this review, we undertook steps to explore cognition over time including, where available, measures that directly assess the impact of cognition on function, using a systematic scoping approach. The specific aims of this review were: (1) to identify and characterize studies that evaluate cognition longitudinally after stroke; (2) to identify the instruments used in these studies and the domains they target; and (3) to map the cognitive domains reported in the studies to a common classification framework, the International Classification of Functioning, Disability and Health (ICF) [35].

## Methods

For this study, we followed the Preferred Reporting Items for Systematic Reviews and Meta-Analyses (PRISMA) [36] as well as the guidelines for systematic scoping studies outlined by Peters et al. [37]. Scoping studies aim to map the key concepts of a research area as well as the main sources and types of evidence available. A scoping study can be undertaken as a stand-alone project, especially when an area is complex or has not been reviewed comprehensively before (p194). Systematic scoping studies follow the same steps as a systematic review, with the distinction that they may or may not involve a quality appraisal phase [37]. Because the goal of this review was to identify and synthesize the available evidence, we did not undertake a formal assessment of the methodological quality. Our protocol was registered with the International Prospective Registry of Systematic Studies PROSPERO in August, 2017 (CRD42017054449). This protocol includes the present systematic scoping review focusing on identifying the available literature on longitudinal evaluation of post-stroke cognition; and a subsequent meta-analysis investigating changes in the trajectory of cognitive function in a subset of the studies included in the present review. For more details, visit: http://www.crd.york.ac.uk/PROSPERO/display_record.php?ID=CRD42017054449

### Searching for relevant studies

Six online libraries were searched: Embase, Pubmed, Web of Science, Cinahl Plus, Medline, and PsycInfo. These libraries were selected on the basis of the disciplines represented, yearly uploaded records, regional databases included, and countries covered. Search limits included peer-reviewed studies, published in English, from January 1^st^, 2001, to February 21^st^, 2019. The commencing year limit was used to capture relatively recent studies and cognitive measures; and was aligned with the year in which the ICF framework was first published.

The ICF framework was selected as it provides a structure to classify health conditions and health domains under the umbrella of functioning. The ICF describes body functions, activities, and participation areas [35] and it is encouraged as a standard language for clinical practice and research. The ICF adopts a tree-like classification in which categories are nested, forming subcategories of the broader category. For instance, mental functions are sub-grouped into global and specific mental functions. These two sub-groups are then further divided into more distinct functions. For each function, a definition is provided based on their essential attributes, qualities or characteristics (p21).

### Selection of studies

Our inclusion criteria encompassed observational and intervention studies. Reports on adults (18 or older), with ischemic, hemorrhagic, or mixed stroke types, with at least two cognitive evaluation time-points, using the same cognitive instrument were included. Additionally, time after stroke onset had to be clearly indicated in the reports. Stroke was defined as a rapidly developing clinical sign of focal (or global) disturbance of cerebral function, with symptoms lasting 24 hours or more, or leading to death, with no apparent cause other than vascular origin [38]. As per this definition, studies looking solely at patients with transient ischemic attack (TIA) or sub-arachnoid hemorrhage (SAH) were excluded. Conference proceedings and unpublished trials were not included in this review. Further exclusions were applied to case studies, non-human studies, and stroke in the pediatric and adolescent population. Full reports that were not available in the libraries mentioned above were requested by contacting the corresponding authors directly. These studies were discarded after one month of no response.

We developed a targeted search strategy using the key words “stroke”, “longitudinal study”, “recovery”, and “outcome” (Table 1). Subject headings and synonyms were used to expand the search, along with wildcards (i.e. “?”), truncations (i.e. cognit*), and boolean operators (i.e. AND, OR). The final search encompassed a combination of key words, synonyms, and operators that were evaluated, piloted for result consistency, and used equivalently across the six online libraries (see full search strategy with results in the S1 Table). We also searched for similar ongoing or published reviews in the Cochrane database, Embase, and PROSPERO using the same combination of key words. Synonyms, wildcards, and truncations in these libraries were used when available.

**Table 1:** Key words, index terms, and synonyms included in the search strategy.

Key words	Stroke	Recovery	Longitudinal studies	Cognition
**Subject Heading^a^**	Cerebrovascular accident [Emtree]	Recovery of function [MeSH]	Longitudinal study [Emtree], Longitudinal studies [MeSH and Thesaurus]	Cognition [MeSH, Emtree, Thesaurus, and CINAHL Heading]
	Cerebrovascular accidents [MeSH]	Recovery [CINAHL heading]	Follow up [Emtree], follow-up studies [MeSH], followup studies [Thesaurus]	Executive function [MeSH, Emtree, Thesaurus, and CINAHL Heading]
	Stroke [Thesaurus and CINAHL heading]	Recovery (disorders) [Thesaurus]	Prospective studies [CINAHL heading]	
**Synonyms^b^**	Cerebro vascular accident, cerebral vascular accident, brain ischemic attack, brain vascular accident, ischemic cerebral attack, ischemic cerebral attack	Functional recovery, function recovery	Longitudinal evaluation, longitudinal survey, prospective study, follow* up, follow up stud*, followup stud*	Cognitive accessibility, cognitive balance, cognitive dissonance, cognitive function, cognitive structure, cognitive symptoms, cognitive task, cognitive thinking, neurobehavioral manifestations, volition, executive functions, executive control, cognit*, attention, memory

^a^Subject heading source provided [in brackets].

^b^Each synonym was searched in the title and abstract.

Titles, abstracts, and full reports were screened by two independent reviewers (JPS and TT). Disagreements were resolved by consensus after reviewed in full, or by a third, senior researcher (LC), who made the final decision. Full reports were then thoroughly inspected for data extraction.

### Charting the data

For this review, all data were extracted as reported in the original papers by one of the review authors (JPS). The following information was extracted from each report: general study descriptors (e.g. author, year of publication, and country), study type (i.e. intervention trial or observational), sample size at baseline, stroke type (e.g. ischemic, lacunar, hemorrhagic, ICH, TIA, or mixed type), and a complete description of the cognitive evaluations performed (instruments used, cognitive domains evaluated, time from stroke onset to evaluation, and type of outcome reported e.g. frequencies, mean scores).

### Collating, summarizing, and reporting the results

General characteristics about each study were collated and summarized in tables and figures. Time from stroke onset to evaluation was used as reported in papers, with a few exceptions: for acute stage reports in which time of onset was unclear, time of admission and baseline evaluations were assumed within the first two weeks of symptom onset, as recommended by recent international guidelines [39–41]

Cognitive evaluations were identified as either full assessments, or sub-tests used as stand-alone assessments. For instance, sub-tests from a larger assessment (i.e. “MMSE-Orientation Subtest”, “Stroop-Naming Subtest”) were considered stand-alone instruments when used in isolation to evaluate a specific cognitive domain.

Cognitive domains were mapped to a mental function of the ICF following a previously published systematic approach [42]. This approach integrates the definitions, inclusions, and exclusions provided by the ICF, in addition to 10 linking rules, designed to generate guided consensus when mapping health measures to an ICF category (see S2 Table for details on how the linking rules were applied to our data). Reliability of these rules has been previously tested, ensuring consistency and comparability of health information [42,43]. In short, we first extracted the “raw” cognitive domains—or domains as reported in the studies. These domains were then mapped to an ICF function independently by two reviewers (JPS and TT). In the case of consensus, the item was linked to the ICF. If there was disagreement, a discussion between the same two reviewers occurred, with direct reference to the ICF definitions and linking rules, until consensus was achieved.

Following the same ICF linking rules, multi-domain tools were classified as evaluating “Global cognition” (chapter 1 of the ICF) when the combination of mental functions evaluated in these tools led to a more general category than the individual mental functions targeted in them (e.g. tool provided a “general” score for all the mental functions evaluated). Accordingly, we assigned “Global cognition” to all the cognitive tools evaluating a combination of global mental functions (ICF functions b110-b139), and specific mental functions (ICF functions b140-b189).

Raw domains were also mapped to a broader semantic category, by the same two reviewers. This step was undertaken to compare the final count of reported domains, versus the ICF domains mapped using the linking rules. Semantic categories were created based on distinct domains that are commonly used in evaluation. For example, “verbal skills”, “verbal fluency”, and “language skills” were grouped under “verbal skills”. Similarly, “long-term memory”, “working memory”, and “verbal memory” were grouped together as “memory skills”.

Agreement in the domains evaluated between studies were calculated by comparing the mapped ICF domains for selected assessments. Agreement levels were interpreted based on the interpretive guidelines for categorical data by Landis and Koch [44].

Data were processed using algorithmic analysis, which was created with the data available from all the included studies. All data pre-processing, agreement calculations, and figures were completed using R version 3.5 [45]. A complete dataset with all the data extracted or analyzed are included in the S3 Table.

## Results

**Fig 1:**
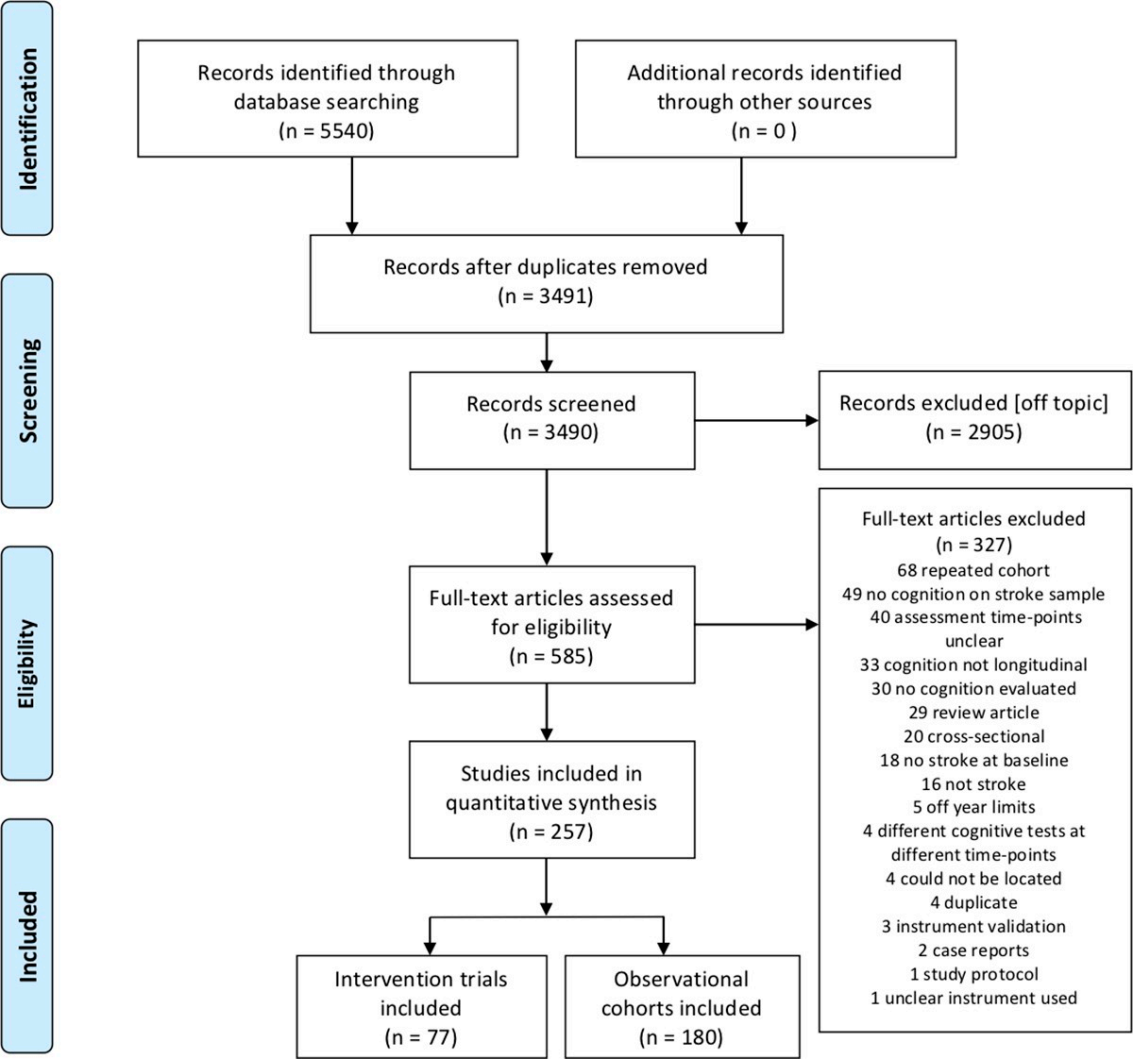
Included studies and reasons for exclusion.

Our search yielded a total of 5,540 records published from Jan. 1^st^, 2001 to February 21^st^, 2019 (Fig 1). After removing duplicates, 3,490 records remained. Independent screening by title and abstract resulted in an initial 585 papers eligible for full-text examination. Independent full-text review of these papers led to the exclusion of another 328 reports. Most excluded studies were publications of the same study cohorts, looking at different outcomes (68 studies, 21%), longitudinal studies not reporting cognitive outcomes (49 studies, 15%), or longitudinal studies with unclear evaluation time-points (e.g. time after stroke was not reported, too wide, or unclear) (40 studies, 12%).

### General study characteristics

**Fig 2:**
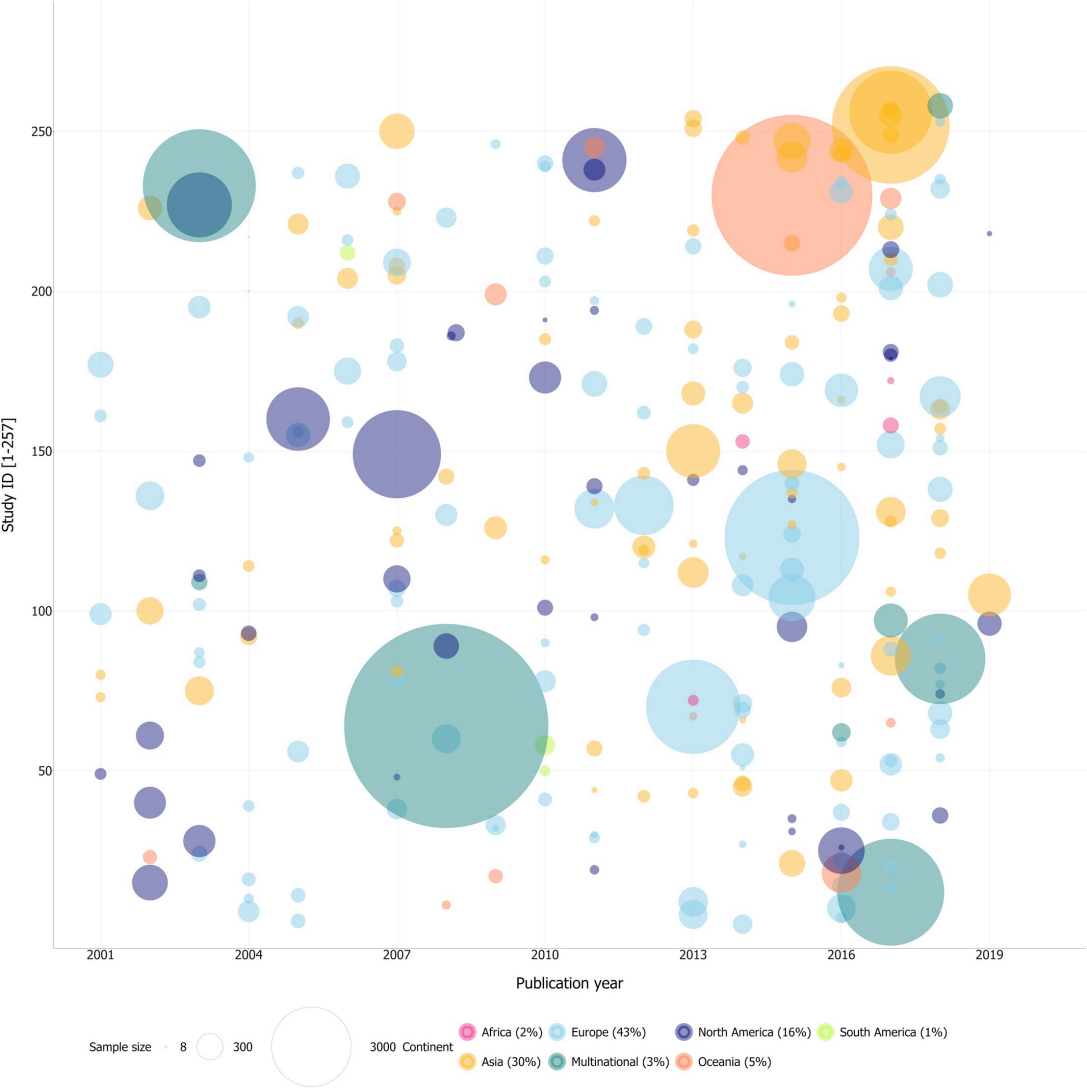
Sample size and origin of included studies (N = 257).

In all, a total of 257 studies, representing a pooled sample of 120,860 stroke patients, were included in this review. The sample size of cohorts ranged from 8 to 20,332 patients. Sixty-two percent of the studies had a sample size of 150 patients or less (see Fig 2 for visual representation). Seventy-seven studies included an intervention and 180 were observational cohorts. Most study samples came from Europe (42%), followed by Asia (30%), North America (16%), Oceania (5%), Africa (2%), and South America (1%). There were also 8 multinational collaborations (3%). A more complete description of these articles can be found in the S2 Table.

As for the timing of cognitive assessments, most included studies (194 reports, 75%) evaluated patients from the first day, and up to two years post-stroke onset (Table 2). Longitudinal descriptive data were available for 9 studies (14,663 patients) in the acute stage (≤ 1 month post stroke onset,); 27 papers (7,037 patients) in the early sub-acute stage (>1 month ≤3 months post stroke onset); and for 38 papers (11,388 patients) in the late sub-acute stage (>3 months and ≤6 months). There were also 46 studies (14,495 patients) reporting longitudinal cognitive outcomes in the early chronic stage (6–12 months post onset); and 36 studies (4,436 patients) in the long-term chronic stage (12–24 months post stroke onset).

**Table 2:** General characteristics of included studies (n = 257).

Study characteristics	Reports cognitive outcomes over time ^a^		Total	
	Yes	No	Count	Percent
**Intervention**	63	14	77	30
**Observational**	137	43	180	70
**Sample size at baseline** (range 8–20,332)				
<25	16	5	21	8
>25 ≤ 50	36	9	45	18
>50 ≤ 100	42	11	53	20
>100 ≤ 150	31	8	39	16
>150 ≤ 500	51	21	72	28
>500 ≤ 1500	11	2	13	5
>1500 ≤ 4000	7	0	7	3
>4000	6	1	7	3
**Furthest follow-up** (range 1 wk-15yrs)				
≤ 1 mo	4	9	13	5
> 1 to ≤ 3 mo	27	13	40	16
> 3 to ≤ 6 mo	38	6	44	17
> 6 to ≤ 12 mo	46	9	55	21
> 12 to ≤ 24 mo	36	10	46	18
> 24 to ≤ 36 mo	21	6	27	10
> 36 to ≤ 60 mo	15	5	20	8
> 60 mo	8	4	12	5

^***a***^ Cognitive outcomes are defined as any descriptive outcome such as a count, percentage, mean, median, with or without their associated measures of dispersion (standard error, range, inter-quartile range, etc)

Papers looking at cognition from two to five years post stroke were also numerous (47 studies, 56,781 patients). From this group, 36 studies (48,912 patients) reported cognitive data over time. Lastly, 12 studies (6,379 patients) had follow-up assessments measuring cognition 5 years post-stroke or after, 8 of which (5,358 patients) reported cognitive outcomes. The two longest follow-ups with outcome data available were reported at 8.3 and 15 years post-stroke [9,46].

### Instruments and domains evaluated

**Fig 3:**
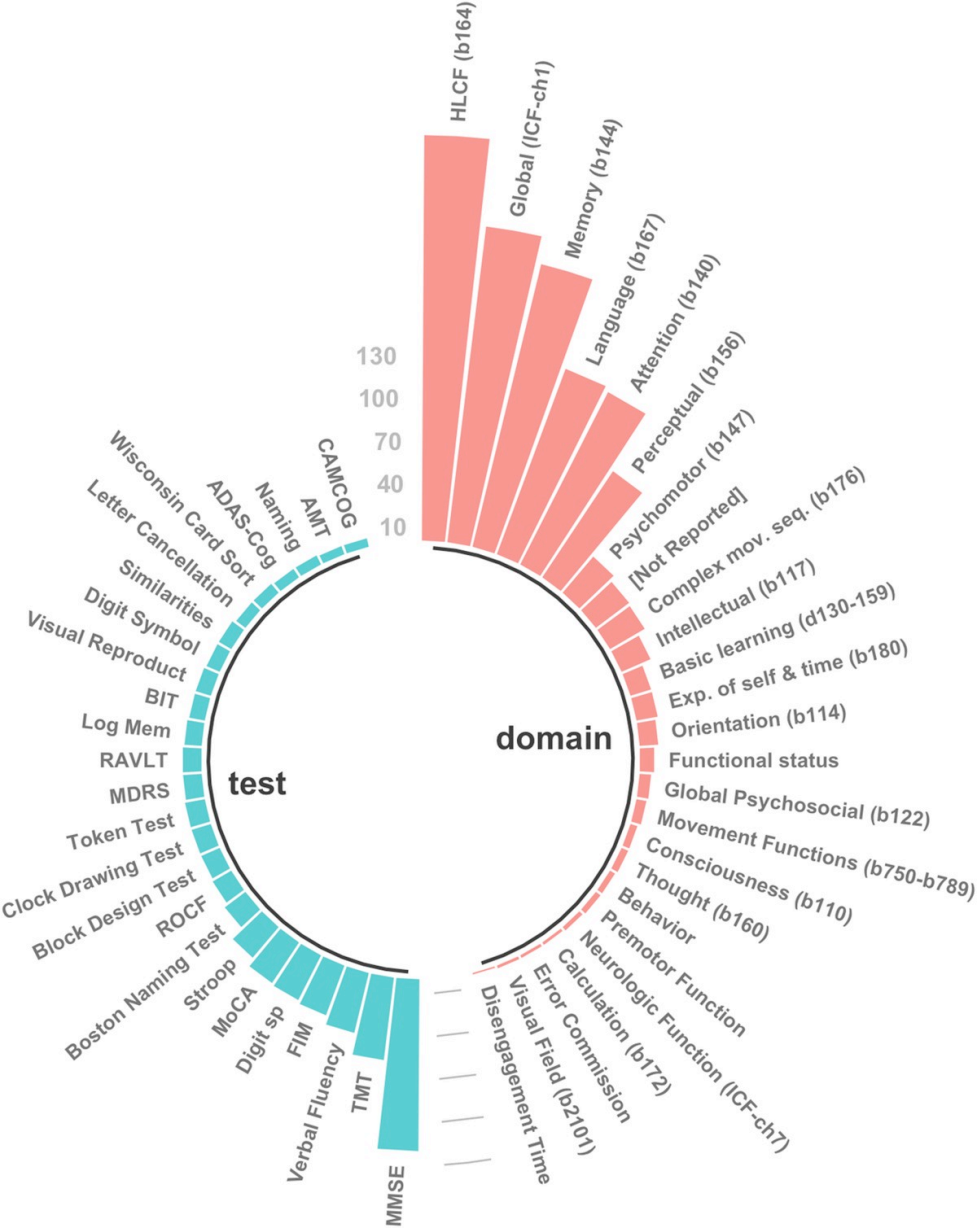
Twenty-five most frequently used cognitive tests and ICF domains across included studies. ADAS-cog, Alzheimer's Disease Assessment Scale; AMT, Abbreviated Mental Test; BIT, Behavioral Inattention Test; Digit sp, Wechsler's Digit Span; FAB, Frontal Assessment Battery; FIM, Functional Independence Measure; HLCF, Higher-Level Cognitive Functioning; Log Mem, Wechsler's Logical Memory; MDRS, Mattis-Dementia Rating Scale; MMSE, Mini-Mental State Examination; MoCA, Montreal Cognitive Assessment; RAVLT, Rey Auditory Verbal Learning Test; ROCF, Rey-Osterrieth Complex Figure; TMT, Trail Making Test.

We recorded 1033 follow-up evaluations across the 257 studies, using 356 unique instruments of cognition (see Fig 3 for the 25 most-commonly used instruments and domains). The majority of the studies (72.4%) used screening tools, that is, brief assessments that screen across multiple cognitive domains in a short time [47]. Among all cognitive tools administered longitudinally, the Mini-Mental State Examination (MMSE) [48] was the most frequently used (117 studies, 46%). Other tools commonly administered longitudinally were the Trail Making Test (17%) [49], different forms of the Verbal Fluency Test (14%), the Functional Independence Measure (cognitive subtest, 14%) [50], the Montreal Cognitive Assessment (13%) [51] the Wechsler Digit Span (11%) [52], and the Stroop test (9%) [53]. Altogether, these seven instruments were used in 209 studies (81%), representing 355 (34%) of the 1033 longitudinal assessment instances recorded.

We identified 317 “raw” domains of cognition, that is, cognitive domains as reported in the studies. Classification of these raw domains into broader semantic categories led to the identification of 95 distinct cognitive domains. Our initial inter-rater agreement level was strong (69%) [44]. After all disagreements were resolved, 302 of the 317 raw domains initially recorded were mapped onto 17 defined mental functions of the ICF. Examples on how we used the ICF mapping rules can be found in the S2 Table. Further, a comprehensive list of all the raw domains recoded, with their final ICF domain assignment can be found in the S1 Fig.

### Domains evaluated by frequently used cognitive instruments

**Fig 4:**
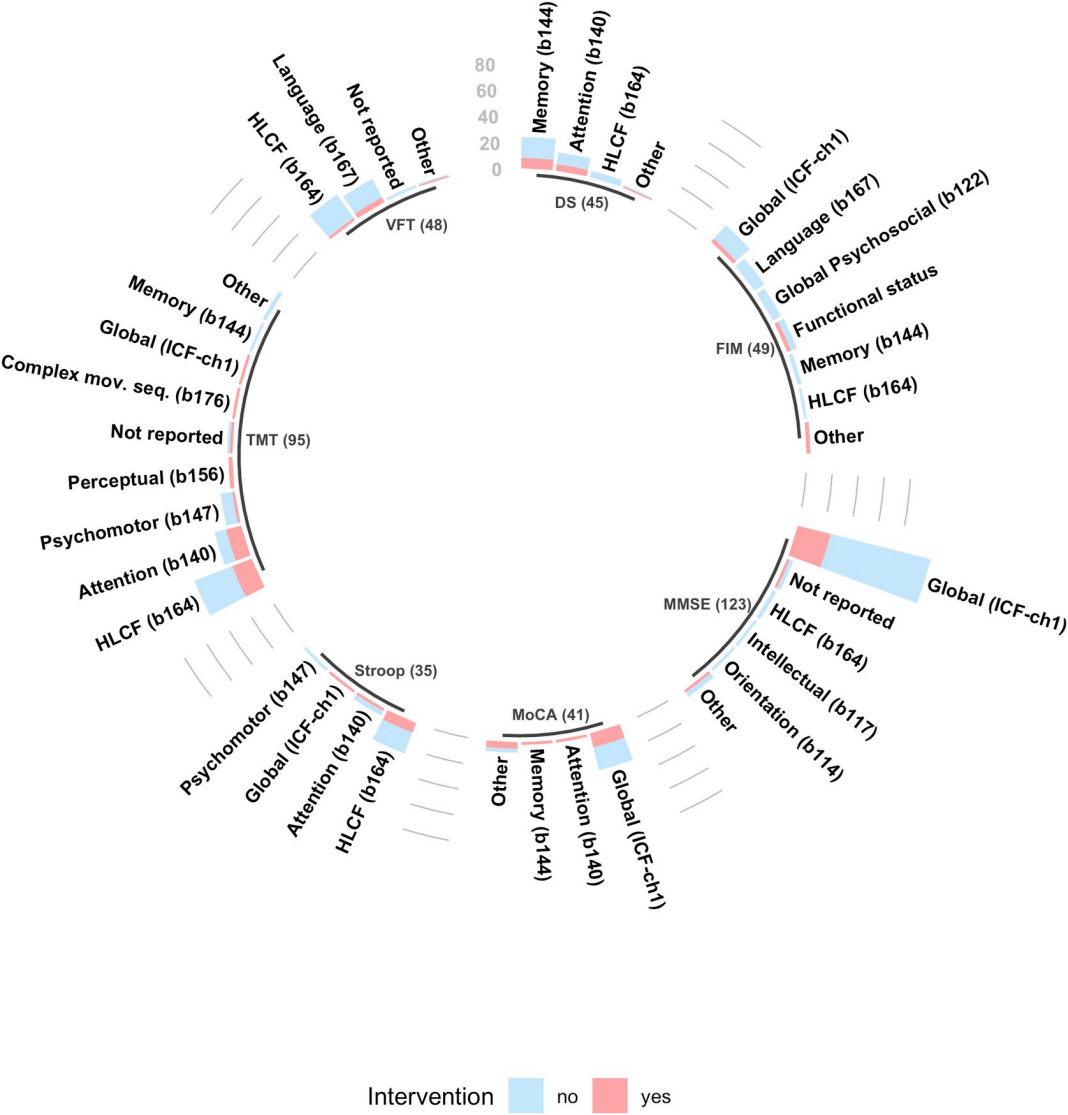
Cognitive tools most commonly used and the ICF functions they evaluate. The top 7 instruments were used in 209 of 257 articles, representing 34% of the evaluations completed across all the studies. The 'Not reported' category was assigned to studies that did not specify what cognitive domain was being evaluated when using a specific assessment; The 'Other' category was assigned to indicate a cognitive domain (or group of cognitive domains) that is different from the ones listed for the specific tool. DS, Wechsler's Digit Span; FIM, Functional Independence; HLCF, Higher-Level Cognitive Functioning; ICF-ch1, International Classification of Functioning and Disability, chapter 1; MMSE, Mini-Mental State Examination; MoCA, Montreal Cognitive Assessment; TMT, Trail Making Test; VFT, Verbal Fluency Test.

Examination of the cognitive domains targeted by the top seven instruments revealed the extent of agreement between studies on a common ICF mental function when using each tool (Fig 4). Observed agreement was very strong only for the MMSE, with 85% agreement on evaluation of global cognition (chapter 1 of the ICF framework), which encompasses the evaluation of global mental functions (ICF functions b110-b139); as well as specific mental functions (ICF functions b140-b189).

**Fig 5:**
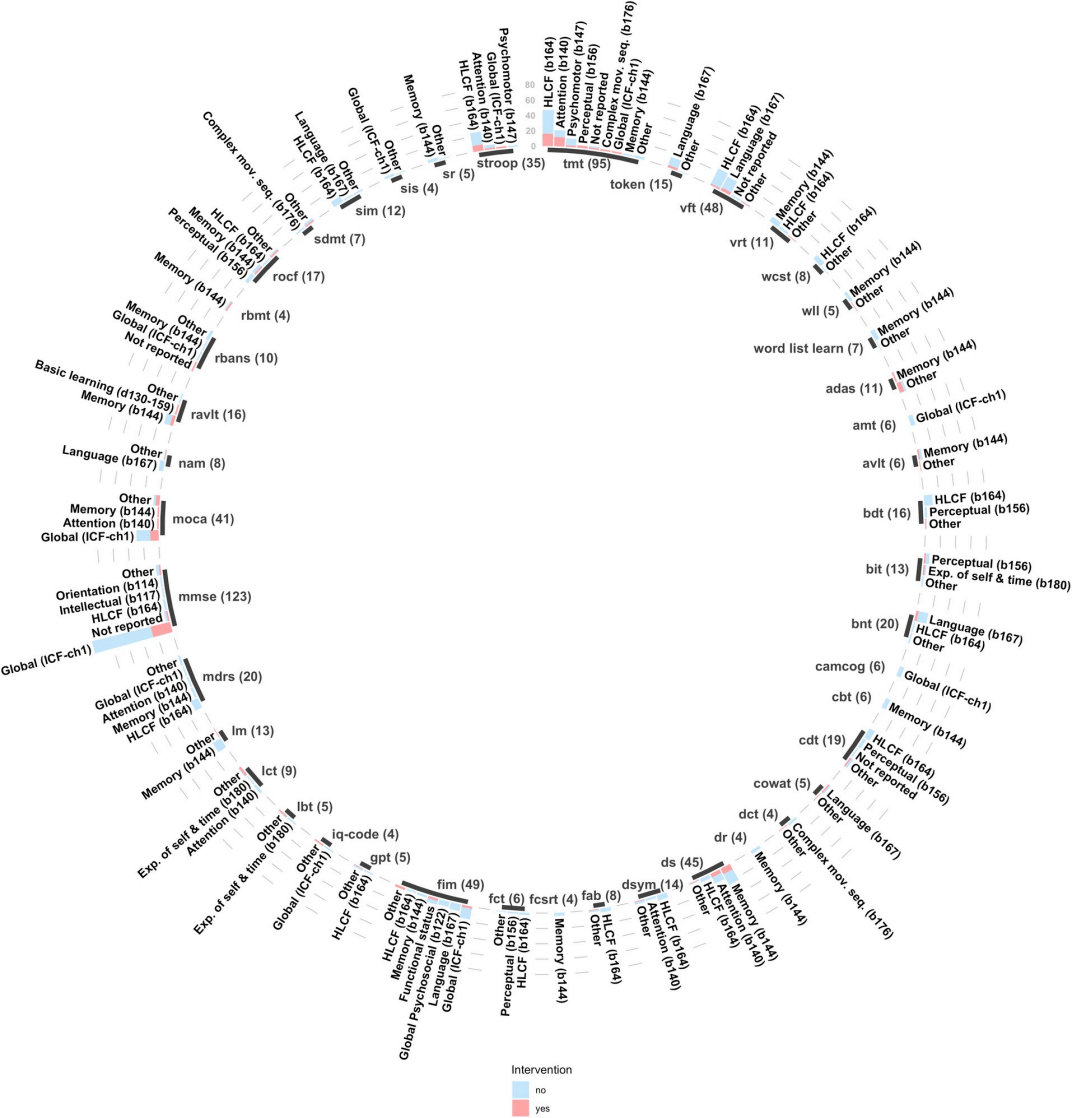
Mental functions from the ICF evaluated by each instrument. The 43 instruments described in this figure were used in 228 of 257 studies, and represent 74% of all the evaluations performed. Only common tools, used more than 3 times, across all papers were used to build this figure; adas, alzheimers disease assessment scale; amt, abbreviated mental test; avlt, auditory verbal learning test; bdt, block design test; bit, behavioral inattention test; camcog, cambridge cognition examination; cbt, corsi blocks test; cdt, clock drawing test; cowat, controlled oral word association test; dct, digit cancel task; dr, delayed recall test; ds, digit span test; dsym, digit symbol test; fab, frontal assessment battery; fct, figure copying test; fim, functional independence measure; gpt, grooved peg test; iq-code, informant questionnaire on cognitive decline in the elderly; lct, letter cancellation test; lm, logical memory test; mdrs, mattis-dementia rating scale; mmse, mini mental examination; moca, montreal cognitive assessment; nam, naming test; ravlt, rey auditory verbal learning test; rbans, repeatable battery for the assessment of neuropsychological status; rocf, rey-osterrieth complex figure test; sdmt, symbol digit modalities test; sim, similarities test; sis, stroke impact scale; sr, story recal test; stroop, stroop test (also called color word interference test); tmt, trail making test; token, token test; vft, verbal fluency test; vrt, visual reproduction test; wcst, wisconsin card sort test; wll, word list learning; wlr, word list recall & recognition.

Agreement for the rest of the instruments varied from weak (FIM, 35% agreement on evaluation of global cognition), to strong (MoCA, 71% agreement on evaluation of global cognition) (Table 3). A summary of the exhaustive analysis performed on most the instruments utilized, and the ICF domains they target can be found in Fig 5.

**Table 3:** Agreement on ICF domains evaluated by the top 7 instruments.

Instrument	Function Evaluated (ICF code)	Agreement level ^a^
MMSE	Global cognition (ICF-ch1)	85% | very strong
TMT	Higher-level cognition (b164)	49%| moderate
VFT	Higher-level cognition (b164)	50% | moderate
FIM-Cog	Global cognition (ICF-ch1)	35% | weak
Digit Span	Memory (b114)	53% | moderate
Stroop	Higher-level cognition (b164)	71% | strong
MoCA	Global cognition (ICF-ch1)	71% | strong

ICF-ch1, International Classification of Functioning and Disability, chapter 1 (mental functions); MMSE, Mini-Mental State Examination; TMT, Trail Making Test; VFT, Verbal Fluency Test; FIM-cog, Functional Independence Measure, cognitive subtest; MoCA = Montreal Cognitive Assessment.

^a^ Agreement level interpretation. Adapted from Landis and Koch (1977)

### Types of longitudinal cognitive outcomes reported in studies

As highlighted in the general study characteristics, 200 studies (78%) reported cognitive outcomes over time and 57 studies did not report any descriptive data on cognition, focusing mostly on reporting model predictions. In general, there were more observational than intervention studies reporting cognitive descriptive outcomes (137 vs. 63 reports). However, when seen proportionally (percentage intervention studies reporting outcomes vs. percentage observational studies reporting outcomes), a higher percentage of intervention studies (82% vs. 76%) had cognitive data available over time. Most studies described cognitive outcomes using summary statistics with a mean and a standard deviation (57%). These were followed by studies reporting non-parametric measures of central tendency (18%), that is, a median or mode accompanied by a range or interquartile range. A third group of studies reported frequencies of impaired/unimpaired individuals over time (15%). Lastly, a fourth group of studies reported incomplete descriptive data (10%), such as mean, median, or mode alone, without a measure of dispersion (standard deviation, inter-quartile range, or range).

## Discussion

The aims of this review were to identify and characterize studies that measure cognition over time; to identify the cognitive instruments used and the domains they target; and to classify cognitive domains under the ICF framework. Our scoping approach helped us organize a large amount of research. A major finding is that there is a sizeable amount of descriptive data available in the literature and for different rehabilitation stages to describe changes in cognition over time. These longitudinal cognitive outcomes span a variety of multi-domain and domain-specific tests, for both intervention trials and observational studies.

Importantly, we found that the vast majority of studies reporting outcomes had scores available with a mean and standard deviation (57%). Very recent efforts in collating longitudinal data have focused on recording the incidence of cognitively impaired patients over time [5,11]. These studies have lacked sufficient data to describe a trajectory of cognition. Our findings indicate that efforts should be redirected towards gathering continuous-level data, as opposed to discrete or dichotomized data. This is a valuable finding as analysis of continuous-level will permit an analysis of cognition that is sensitive to small changes in scores over time [54,55].

In relation to the cognitive instruments used, our findings are consistent with previous reports [17,56] in that the MMSE continues to be the preferred assessment. A positive observation, as far as using newer and more sensitive cognitive evaluation tools is concerned, was that studies reporting cognition with the MoCA were included only in the more recent studies (2014 and onwards). This might be suggestive of a shift towards the use of tools that are more sensitive to mild cognitive impairment [57,58]. The shift is also clinically relevant as it targets the mild stroke population, which has been defined as the most prevalent type of stroke [23]. Stroke specific scales such as the Oxford Cognitive Screen [59], and the Cognitive Assessment Scale for stroke Patients (CASP) [60] were not used in any of the included studies. These assessments have been described in the literature since 2015, yet were not used in any of the longitudinal studies identified. It is recommended that tools such as these could be included in future investigations looking at post-stroke cognition.

Our findings also highlight the overwhelming preference to use cognitive tools that focus on impairment level, as opposed to activity limitation and/or measures that directly link cognition with functional activities. In this regard, evaluation of cognition at the performance level is an urgent need in stroke rehabilitation, as highlighted by the present and previous systematic reports [34]. Examples of tools that evaluate functional cognition at the performance level include the Executive Function Performance Test (EFPT) [61], the Complex Task Performance Assessment (CTPA) [18,62], and the Cognitive Performance Test (CPT) [63]. The advantage of using these tools is that they provide insights on cognition in the context of a task, using ecologically valid methods [64]. Further validation of these tools with larger samples could lead to a better understanding of stroke survivors’ challenges when facing tasks that are referred to as highly cognitively demanding (i.e. work, driving, money management). Moreover, functionally-oriented measures are likely to have particular relevance in longitudinal studies where the link between cognition and functional activities is core to recovery outcomes. It is recommended that future studies include performance-based cognitive outcomes in order to determine how these constructs are supporting or inhibiting the functional recovery of stroke.

One novel aspect of this report is that we used the ICF to conceptualize and classify cognitive function. While we found no studies reporting outcomes with this framework, most domains found in the papers could be categorized into an aspect of mental functioning defined by the ICF (e.g. higher-level cognitive function, general cognition, memory, language). From the 15 domains that we could not categorize, learning was the only major function routinely evaluated (9 studies) and mapped into its own dedicated ICF function (Basic Learning, codes 130–159). The high number of domains that we could classify is a favorable result that suggests good feasibility for this conceptual framework to be used for dissemination of findings, and to facilitate communication across rehabilitation, disability fields, and science [35]. Other frameworks that clearly separate functional from domain-specific cognition [65,66] should also be considered when looking at cognition in the context of learning, activity performance, and rehabilitation.

Strengths of this review include: a targeted search strategy, which was piloted, and used consistently across search libraries; the use of three independent researchers to determine study inclusion; and the analysis of results through decision-making algorithms, which minimized human error when handling and restructuring large amounts of information. Additionally, the scoping nature of this study allowed us to look at the evaluation of post-stroke cognition broadly, which resulted in the inclusion of different types of research. Further, our search strategy captured studies of people with different post-stroke syndromes or related conditions, such as dementia, aphasia, neglect, fatigue, and depression, which represent important subgroups of the stroke population.

Possible limitations of this review include the exclusive use of peer-reviewed literature, which may not be reflective of the assessments used in everyday clinical practice. Another limitation of our study is that our search may not have been able to capture all the published literature assessing cognition over time. For example, as part of the review process the authors became aware of a cross-sectional study that included a reassessment in a subset of the cohort [67], but was missed in our search. Future studies might consider inclusion of additional search terms such as ‘reassessment’. Additionally, our inclusion was limited to articles published from 2001 to 2019. The use of these time limits may have biased our analysis and conclusions towards newer cognitive assessments, thus under-representing the more traditional evaluation tools. Further, even though our data extraction process was meticulous and done without interpretation, it may have had some inaccuracies/bias as it was completed by a single researcher. Finally, this study did not include a risk of bias assessment. Because of the number of studies found and their very heterogeneous nature, evaluating the risk of bias both qualitatively and quantitatively is an important next step recommended that can help identify the best evidence available in this topic.

Future research looking at post stroke cognition should incorporate and weight the role of different factors that may influence cognitive recovery, such as demographic variables, different types of stroke, and the presence of baseline comorbidities and risk factors. Incorporation of these variables should lead to a more thorough and comprehensive understanding of the longitudinal trajectory of cognitive function after stroke.

## Conclusion

The systematic scoping review undertaken found a substantial number of papers reporting cognitive outcomes in the stroke population longitudinally. While the scoping nature of this report does not allow for a thorough quantitative description of cognition over time, analysis of these data is necessary and timely to accurately describe the trajectory of cognitive function after stroke. The large number of assessments found highlights the work that needs to be done in order to organize the current knowledge in cognitive evaluation after stroke. Systematic approaches can help in the process of achieving consensus on which cognitive measures may be used to target different cognitive domains. Finally, as suggested by the World Health Organization and Hachinski and colleagues over a decade ago [13,35], the advance in the development of guidelines and protocols for testing will facilitate replication of studies, as well as transferability of better evaluation strategies among different clinical populations. We are at a stage that we can use better standardized procedures to effectively move the field towards a more accurate and systematic analysis of post-stroke cognition. This in turn will serve to advance our understanding of the trajectory of cognition and its functional impact over time.

## Supporting information

S1 Table Detailed search strategy for all online libraries. (DOCX)

S2 Table ICF linking rules with examples. (DOCX)

S3 Table Included studies and abbreviations used to describe cognitive tools. (DOCX)

S4 Table PRISMA checklist. (DOC)

S1 Fig Recoding of cognitive domains and instruments. (PDF)
