# Supplementary material for: Longitudinal evaluation of cognition after stroke – A systematic scoping review
Source: PLoS One. 2019 Aug 29;14(8):e0221735. doi: 10.1371/journal.pone.0221735 (PMC6715188; doi:10.1371/journal.pone.0221735)
Supplement: S1 Table — (DOCX) [file pone.0221735.s001.docx]

### Supplementary Table 1

|  | Detailed Search Strategy for all Libraries | | | | | | | Index Terms | | |
| --- | --- | --- | --- | --- | --- | --- | --- | --- | --- | --- |
|  |  |  |  |  |  |  |  | Results on February 21st, 2019 | | |
| **LOGIC GRID EMBASE** | | | | | | | | | | |
|  | **Stroke** |  | **Cognition** |  | **Longitudinal Study** |  | **Outcome** | |  | **Search Limits** |
|  |  | **AND** |  | **AND** |  | **AND** |  | |  |  |
| OVID | cerebrovascular accident.sh. OR stroke.ti. OR stroke.ab. OR cerebro vascular accident.ti. OR cerebro vascular accident.ab. OR cerebral vascular accident.ti. OR cerebral vascular accident.ab. OR brain ischaemic attack.ti. OR brain ischaemic attack.ab. OR brain ischemic attack.ti. OR brain ischemic attack.ab. OR brain vascular accident.ti. OR brain vascular accident.ab. OR CVA.ti. OR CVA.ab. OR ischaemic cerebral attack.ti. OR ischaemic cerebral attack.ab. OR ischemic cerebral attack.ti. OR ischemic cerebral attack.ab. |  | cognition.sh. OR cognit*.ti. OR cognit*.ab. OR cognitive accessibility.ti. OR cognitive accessibility.ab. OR cognitive balance.ti. OR cognitive balance.ab. OR cognitive dissonance.ti. OR cognitive dissonance.ab. OR cognitive function*.ti. OR cognitive function*.ab. OR cognitive structure*.ti. OR cognitive structure*.ab. OR cognitive symptom*.ti. OR cognitive symptom*.ab. OR cognitive task*.ti. OR cognitive task*.ab. OR cognitive thinking.ti. OR cognitive thinking.ab. OR neurobehavioral manifestations.ti. OR neurobehavioral manifestations.ab. OR neurobehavioural manifestations.ti. OR neurobehavioural manifestations.ab. OR volition.ti. OR volition.ab. OR executive function.sh. OR executive function*.ti. OR executive function*.ab. OR executive control*.ti. OR executive control*.ab. OR memory.sh. OR memory.ti. OR memory.ab. OR attention.sh OR attention.ti. OR attention.ab. |  | longitudinal study.sh. OR longitudinal stud*.ti. OR longitudinal stud*.ab. OR longitudinal eval*.ti. OR longitudinal eval*.ab. OR longitudinal survey.ti. OR longitudinal survey.ab. OR prospective stud*.ti. OR prospective stud*.ab. OR follow up.sh. OR follow* up.ti. OR follow* up.ab. OR follow up stud*.ti. OR follow up stud*.ab. |  | outcome assessment.sh. OR outcome measurement.sh. OR patient outcome assessment.sh. OR treatment outcome.sh. OR outcome*.ti. OR outcome*.ab. OR measure*.ti. OR measure*.ab. OR asses*.ti. OR asses*.ab. OR eval*.ti. OR eval*.ab. | |  | Peer reviewed, human, english, 2001-Current, adults (18+) , exclude medline  308 |
| **LOGIC GRID PUBMED** | | | | | | | | | | |
|  | **Stroke** |  | **Cognition** |  | **Longitudinal Study** |  | **Outcome** | |  | **Search Limits** |
|  |  | **AND** |  | **AND** |  | **AND** |  | |  |  |
|  | stroke[mh] OR stroke[tiab] OR cerebrovascular accident[tiab] OR cerebro vascular accident[tiab] OR cerebral vascular accident[tiab] OR brain ischaemic attack[tiab] OR brain ischemic attack[tiab] OR brain vascular accident[tiab] OR CVA[tiab] OR ischaemic cerebral attack[tiab] OR ischemic cerebral attack[tiab] |  | cognition[mh] OR cognit*[tiab] OR cognitive accessibility[tiab] OR cognitive balance[tiab] OR cognitive dissonance[tiab] OR cognitive function*[tiab] OR cognitive structure*[tiab] OR cognitive symptom*[tiab] OR cognitive task*[tiab] OR cognitive thinking[tiab] OR neurobehavioral manifestation*[tiab] OR neurobehavioural manifestation*[tiab] OR volition[tiab] OR executive function[mh] OR executive function*[tiab] OR executive control*[tiab] OR memory[mh] OR memory[tiab] OR attention[mh] OR attention[tiab] |  | longitudinal studies[mh] OR longitudinal study[tiab] OR longitudinal stud*[tiab] OR longitudinal eval*[tiab] OR longitudinal survey[tiab] OR prospective study[tiab] OR prospective stud*[tiab] OR follow-up studies[mh] OR follow up stud*[tiab] OR followup study[tiab] OR follow* up[tiab] |  | outcome and process assessment[mh] OR outcome and process assessment*[tiab] OR outcome measure*[tiab] OR outcome assessment*[tiab] OR patient outcome assessment*[tiab] OR treatment outcome*[tiab] OR outcome*[tiab] OR measure*[tiab] OR asses*[tiab] OR eval*[tiab] | |  | Journal, 2001-present, english, core clinical journals, nursing journals, adults 19+, exclude medline  287 |
| **LOGIC GRID WEB OF SCIENCE** | | | | | | | | | | |
|  | **Stroke** |  | **Cognition** |  | **Longitudinal Study** |  | **Outcome** | |  | **Search Limits** |
|  |  | **AND** |  | **AND** |  | **AND** |  | |  |  |
|  | TS=(stroke OR "cerebrovascular accident" OR "cerebro vascular accident" OR "cerebral vascular accident" OR "brain ischaemic attack" OR "brain ischemic attack" OR "brain vascular accident" OR CVA OR "ischaemic cerebral attack" OR "ischemic cerebral attack") |  | TS=(cognit* OR "cognitive accessibility" OR "cognitive balance" OR "cognitive dissonance" OR "cognitive function*" OR "cognitive structure*" OR "cognitive symptom*" OR "cognitive task*" OR "cognitive thinking" OR "neurobehavioral manifestation*" OR volition OR "executive function*" OR "executive control*" OR memory OR attention) |  | TS=("longitudinal stud*" OR "longitudinal eval*" OR "longitudinal survey*" OR "prospective stud*" OR "followup stud*" OR "follow up stud*" OR "follow* up") |  | TS=(outcome and process assessment* OR outcome measure* OR outcome asses* OR patient outcome assessment* OR treatment outcome* OR outcome* OR measure* OR asses* OR eval*) | |  | article or review, english, 2005-2017  2624 |
| **LOGIC GRID CINAHL** | | | | | | | | | | |
|  | **Stroke** |  | **Cognition** |  | **Longitudinal Study** |  | **Outcome** | |  | **Search Limits** |
|  |  | **AND** |  | **AND** |  | **AND** |  | |  |  |
|  | MH stroke+ OR TI "cerebrovascular accident" OR TI "cerebro vascular accident" OR TI "cerebral vascular accident" OR TI "brain ischaemic attack" OR TI "brain ischemic attack" OR TI "brain vascular accident" OR TI CVA OR TI "ischaemic cerebral attack" OR TI "ischemic cerebral attack" OR AB "cerebrovascular accident" OR AB "cerebro vascular accident" OR AB "cerebral vascular accident" OR AB "brain ischaemic attack" OR AB "brain ischemic attack" OR AB "brain vascular accident" OR AB CVA OR AB "ischaemic cerebral attack" OR AB "ischemic cerebral attack" |  | MH cognition+ OR TI cognit* OR TI "cognitive accessibility" OR TI "cognitive balance" OR TI "cognitive dissonance" OR TI "cognitive function*" OR TI "cognitive structure*" OR TI "cognitive symptom*" OR TI "cognitive task*" OR TI "cognitive thinking" OR TI "neurobehavioral manifestation*" OR TI "neurobehavioural manifestation*" OR TI volition MH "executive function"+ OR TI "executive function*" OR TI "executive control*" OR AB cognit* OR AB "cognitive accessibility" OR AB "cognitive balance" OR AB "cognitive dissonance" OR AB "cognitive function*" OR AB "cognitive structure" OR AB "cognitive symptom*" OR AB "cognitive task*" OR AB "cognitive thinking" OR AB "neurobehavioral manifestation*" OR AB "neurobehavioural manifestation*" OR AB volition OR AB "executive function*" OR AB "executive control*" OR MH memory+ OR TI memory OR AB memory OR MH attention+ OR TI attention OR AB attention |  | MH "prospective studies+" OR TI "longitudinal stud*" OR TI "longitudinal eval*" OR TI "longitudinal survey" OR TI "prospective stud*" OR AB "'longitudinal study" OR AB "longitudinal studies" OR AB "longitudinal eval*" OR AB "longitudinal survey" OR AB "prospective stud*" OR TI "follow up stud*" OR AB "follow up stud*" OR TI "followup stud*" OR AB "followup stud*" OR TI "follow* up" OR AB "follow* up" |  | MH "outcome assessment" OR TI "outcome assessment*" OR AB "outcome assessment*" OR MH "treatment outcomes+" OR TI "treatment outcome*" OR AB "treatment outcome*" OR MH "patient-reported outcomes+" OR TI "patient-reported outcome*" OR AB "patient-reported outcome*" OR TI outcome* OR TI measure* OR TI asses* OR TI eval* OR AB outcome* OR AB measure* OR AB asses* OR AB eval* | |  | Peer reviewed, english, 2005-2017, exclude medline, human, all adult  37 |
| **LOGIC GRID MEDLINE** | | | | | | | | | | |
|  | **Stroke** |  | **Cognition** |  | **Longitudinal Study** |  | **Outcome** | |  | **Search Limits** |
|  |  | **AND** |  | **AND** |  | **AND** |  | |  |  |
| OVID | cerebrovascular accidents.ti OR stroke.sh OR stroke.ti. OR cerebrovascular accident.ti OR cerebro vascular accident.ti OR cerebral vascular accident.ti OR brain ischaemic attack.ti OR brain ischemic attack.ti OR brain vascular accident.ti OR CVA.ti OR ischaemic cerebral attack.ti OR ischemic cerebral attack.ti OR cerebrovascular accident.ab OR stroke.ab OR cerebro vascular accident.ab OR cerebral vascular accident.ab OR brain ischaemic attack.ab OR brain ischemic attack.ab OR brain vascular accident.ab OR CVA.ab OR ischaemic cerebral attack.ab OR ischemic cerebral attack.ab |  | cognition.sh OR cognit*.ti OR cognitive accessibility.ti OR cognitive balance.ti OR cognitive dissonance.ti OR cognitive function*.ti OR cognitive structure*.ti OR cognitive symptom*.ti OR cognitive task*.ti OR cognitive thinking.ti OR neurobehavioral manifestation*.ti OR neurobehavioural manifestation*.ti OR volition.ti OR executive function.sh OR executive function*.ti OR executive control*.ti OR cognit*.ab OR cognitive accessibility.ab OR cognitive balance.ab OR cognitive dissonance.ab OR cognitive function*.ab OR cognitive structure*.ab OR cognitive symptom*.ab OR cognitive task*.ab OR cognitive thinking.ab OR neurobehavioral manifestation*.ab OR neurobehavioural manifestation*.ab OR volition.ab OR executive function*.ab OR executive control*.ab OR memory.sh OR memory.ti. OR memory.ab. OR attention.sh OR attention.ti. OR attention.ab. |  | longitudinal studies.sh OR prospective studies.sh OR longitudinal study.ti OR longitudinal studies.ti OR longitudinal evaluation.ti OR longitudinal survey.ti OR prospective study.ti OR longitudinal study.ab OR longitudinal studies.ab OR longitudinal evaluation.ab OR longitudinal survey.ab OR prospective study.ab OR follow-up studies.sh OR follow-up study.ti OR follow-up study.ab OR followup study.ti OR followup study.ab OR follow* up.ti OR follow* up.ab |  | outcome assessment.sh. OR outcome assessment*.ti. OR outcome assessment*.ab. OR process assessment.sh. OR treatment outcome.sh. OR treatment outcome*.ti. OR treatment outcome*.ab. OR measure*.ti. OR measure*.ab. OR asses*.ti. OR asses*.ab. OR eval*.ti. OR eval*.ab. | |  | 2005-2017, all adult 19 plus years, english, humans, journal article, medline |
|  |  |  |  |  |  |  |  | |  | 1800 |
| **LOGIC GRID PSYCINFO** | | | | | | | | | | |
|  | **Stroke** |  | **Cognition** |  | **Longitudinal Study** |  | **Outcome** | |  | **Search Limits** |
|  |  | **AND** |  | **AND** |  | **AND** |  | |  |  |
|  |  |  |  |  |  |  |  | |  |  |
| OVID | cerebrovascular accidents.sh OR stroke.ti OR cerebrovascular accident.ti OR cerebro vascular accident.ti OR cerebral vascular accident.ti OR brain ischaemic attack.ti OR brain ischemic attack.ti OR brain vascular accident.ti OR CVA.ti OR ischaemic cerebral attack.ti OR ischemic cerebral attack.ti OR cerebrovascular accident.ab OR stroke.ab OR cerebro vascular accident.ab OR cerebral vascular accident.ab OR brain ischaemic attack.ab OR brain ischemic attack.ab OR brain vascular accident.ab OR CVA.ab OR ischaemic cerebral attack.ab OR ischemic cerebral attack.ab |  | cognition.sh OR cognit*.ti OR cognitive accessibility.ti OR cognitive balance.ti OR cognitive dissonance.ti OR cognitive function*.ti OR cognitive structure*.ti OR cognitive symptom*.ti OR cognitive task*.ti OR cognitive thinking.ti OR neurobehavioral manifestation*.ti OR neurobehavioural manifestation*.ti OR volition.ti OR executive function.sh OR executive function*.ti OR executive control*.ti OR cognit*.ab OR cognitive accessibility.ab OR cognitive balance.ab OR cognitive dissonance.ab OR cognitive function*.ab OR cognitive structure*.ab OR cognitive symptom*.ab OR cognitive task.ab OR cognitive thinking.ab OR neurobehavioral manifestation*.ab OR neurobehavioural manifestation*.ab OR volition.ab OR executive function*.ab OR executive control*.ab OR memory.sh OR memory.ti. OR memory.ab. OR attention.sh OR attention.ti. OR attention.ab. |  | longitudinal studies.sh OR prospective studies.sh OR longitudinal stud*.ti OR longitudinal eval*.ti OR longitudinal survey.ti OR prospective stud*.ti OR longitudinal stud*.ab OR longitudinal eval*.ab OR longitudinal survey.ab OR prospective stud*.ab OR followup studies.sh OR follow up stud*.ti OR follow up stud*.ab OR followup stud*.ti OR followup stud*.ab OR follow* up.ti OR follow* up.ab |  | treatment outcomes.sh. OR treatment outcome*.ti. OR treatment outcome*.ab. OR psychotherapeutic outcomes.sh. OR psychotherapeutic outcome*.ti. OR psychotherapeutic outcome*.ab. OR treatment effectiveness evaluation.sh. OR treatment effectiveness eval*.ti. OR treatment effectiveness evaluation.ab. OR measure*.ti. OR measure*.ab. OR asses*.ti. OR asses*.ab. OR eval*.ti. OR eval*.ab. | |  | 2005-current, adulthood 18 years and older, peer reviewed journal, english, human  484 |

Total 5,540 studies
