# Supplementary material for: Longitudinal evaluation of cognition after stroke – A systematic scoping review
Source: PLoS One. 2019 Aug 29;14(8):e0221735. doi: 10.1371/journal.pone.0221735 (PMC6715188; doi:10.1371/journal.pone.0221735)
Supplement: S2 Table — (DOCX) [file pone.0221735.s002.docx]

| **Supplementary Table 2 – ICF linking rules and examples, adapted from Cieza et al., 2002 [1]** | | |
| --- | --- | --- |
| **Rule** | **Original Rule Description** | **Adapted Example** |
| 1 | Before one links health-status measures to the ICF categories, one should have acquired good knowledge of the conceptual and taxonomical fundamentals of the ICF, as well as of the chapters, domains and categories of the detailed classification, including definitions |  |
| 2 | Each item of a health-status measure should be linked to the most precise ICF category | Assessments described as evaluating "dementia" were linked to "intellectual functions (b117)". The ICF explicitly defines dementia under general mental functions; excluding it from memory (b144), thought (b160), or higher-level cognitive functions (b164) |
| 3 | If a single item encompasses different constructs, the information in each construct should be linked | The FIM-cog [2] encompasses constructs related to mental functions (e.g. expression and memory); and contextual constructs (e.g. assisted expression using a communication device). Therefore, both the *mental function constructs* (ICF Mental Functions, ICF chapter 1) as well as the *contextual constructs* (Environmental Factors, chapter2) from this assessment should be linked |
| 4 | All constructs of the item to be linked have to be highlighted (e.g. italicized, bold, or underlined. | In our case we have italicized the mental function and explicitly mentioned its code in parenthesis).  Item 3 of the Montreal Cognitive Assessment (MoCA) [3] refers to *Memory (b144)* |
| 5 | The response options of an item are linked if they refer to additional constructs | Item 2 of the Stroke Impact Scale (SIS) [4] addresses *memory* and *thinking.*  However, the response options under this item also address other mental functions. For instance:  Sub-item a) “*Remember* things that people just told you?” 🡪 *remember* is linked with *memory (b144)*  e) “*Concentrate?”* 🡪 *concentrate* is linked with *attention (b140)*  f) *Think quickly? 🡪* linked with *Higher-level cognition (b164)* |
| 6 | If the content of an item is not explicitly named in the corresponding ICF category, then the “other specified” option at the third and fourth coding level of the ICF classification is linked. The additional information not covered by the ICF classification is documented. | The *initiation* item of the Executive Function Performance Test (EFPT) [5] is not defined in the ICF mental functions at the third (b164), or fourth coding level (b1640-b1646). Therefore, because this evaluation is specific to higher-level cognition, the function was linked to Higher-level cognitive function, other specified (b1647) |
| 7 | If the content of an item is more general than the corresponding ICF category, then the code of the higher level is linked | The MoCA, overall score, encompasses *global mental functions* (orientation), as well as *specific mental functions* (attention, memory, psychomotor, perceptual, higher-level cognition, and language). Therefore, because both *general* and *specific mental functions* are linked, the level above is all mental functions (Mental Functions b110 through b189; or ICF-chapter 1) |
| 8 | If the content of an item is more general than any ICF category but otherwise the item specifies by examples partial aspects of the concept contained in one or more ICF categories, then the “unspecified” option of the ICF classification is linked (Code 99 for the second coding level, Code 9 for third and fourth coding levels) | In the Stroke Impact Scale (SIS), under memory and thinking, item c-In the past week, how difficult was it for you to r*emember* to do things (e.g. *keep scheduled appointments* or *take medication*)? Is linked to *memory (b144);* *Interpersonal interactions and relationships, other unspecified (d799);* and *looking after one’s health (d570)* |
| 9 | If the information provided by the item is not sufficient for making a decision about which ICF category the item should be linked to, this item is assigned nd (not definable). | Item 7 of the Mental Slowness Questionnaire (MSQ) [6] states – “*4. Mental recovery* - If you have to take a break, how long do you need to recover after you have worked ‘until you drop’ or are no longer able to concentrate on what you are doing?”  *Mental recovery* would be assigned the nd (not definable) category under the mental functions construct |
| 10 | If an item is not contained in the ICF classification, then this item is assigned nc (not covered by ICF). | *Premotor functions* were evaluated in two of the included studies (Dacosta-Aguayo et al., 2014; and Grau-Olivares et al., 2010) using Luria's sequences. *Premotor functions* were coded as nc (not covered by the ICF) |
| ICF, International Classification of Functioning, Disability, and Health.  Examples were adapted from Cieza et. al., 2002, table 1, p.206; linking rules are the same as reported in the original paper. For rule #6 we did not use the sub-items a) and b) | | |

***References***

1. Cieza A, Brockow T, Ewert T, Amman E, Kollerits B, Chatterji S, et al. Linking health-status measurements to the international classification of functioning, disability and health. J Rehabil Med. 2002;34: 205–210.

2. Keith RA, Granger CV, Hamilton BB, Sherwin FS. The functional independence measure. Adv Clin Rehabil. 1987;1: 6–18.

3. Nasreddine ZS, Phillips NA, Bédirian V, Charbonneau S, Whitehead V, Collin I, et al. The Montreal Cognitive Assessment, MoCA: a brief screening tool for mild cognitive impairment. J Am Geriatr Soc. 2005;53: 695–699.

4. Duncan PW, Reker DM, Horner RD, Samsa GP, Hoenig H, LaClair BJ, et al. Performance of a mail-administered version of a stroke-specific outcome measure, the Stroke Impact Scale. Clin Rehabil. 2002;16: 493–505. doi:10.1191/0269215502cr510oa

5. Baum CM, Connor LT, Morrison T, Hahn M, Dromerick AW, Edwards DF. Reliability, validity, and clinical utility of the Executive Function Performance Test: a measure of executive function in a sample of people with stroke. Am J Occup Ther Off Publ Am Occup Ther Assoc. 2008;62: 446–455.

6. Johansson B, Starmark A, Berglund P, Rodholm M, Ronnback L. A self-assessment questionnaire for mental fatigue and related symptoms after neurological disorders and injuries. Brain Inj. 2010;24: 2–12. doi:10.3109/02699050903452961
