# Supplementary material for: Longitudinal evaluation of cognition after stroke – A systematic scoping review
Source: PLoS One. 2019 Aug 29;14(8):e0221735. doi: 10.1371/journal.pone.0221735 (PMC6715188; doi:10.1371/journal.pone.0221735)
Supplement: S3 Table — (DOCX) [file pone.0221735.s003.docx]

#

# Assessing cognitive function longitudinally after stroke – A systematic scoping review

# Supplementary Table #3 - included studies and abbreviations used

[Cognitive evaluation descriptions for 257 stroke samples]

| **ID** | **Author and Country** | **Year** | **Title** | **Trial** | **Sample** | **Stroke type** | **Furthest followup (months)** | **Cognitive domain (instrument as reported by authors)** | **Evaluation context** | **Data reported** |
| --- | --- | --- | --- | --- | --- | --- | --- | --- | --- | --- |
| 1 | Cherney et al. (USA) | 2001 | **Recovery of functional status after right hemisphere stroke: relationship with unilateral neglect** | no | 52 | Right hemisphere stroke (CT diagnosed) | 4.0 | **Memory, problem-solving, social interaction, comprehension, and expression** (FIM); **and communication** (RIC-FAS) | Trained personnel administered tests, 3 months post discharge obtained by telephone interview with the patient and/or caregiver | mean & SD |
| 2 | Eldar et al. (Israel) | 2001 | **Quality of care for urinary incontinence in a rehabilitation setting for patients with stroke. Simultaneous monitoring of process and outcome** | no | 37 | Ischemic and hemorrhagic | 4.3 | **Cognition** (FIM) | Registered nurse in charge of follow-ups | no raw data |
| 3 | Fong et al. (China) | 2001 | **Relationship of motor and cognitive abilities to functional performance in stroke rehabilitation** | no | 37 | First stroke | 1.6 | **Cognitive function** (NCSE and FIM) | An experienced occupational therapist was responsible for administering all the assessments to the patients | no raw data |
| 4 | Henon et al. (France) | 2001 | **Poststroke dementia: incidence and relationship to prestroke cognitive decline** | no | 202 | Ischemic or hemorrhagic | 42.0 | **Cognitive impairment** (MMSE)**; global cognitive functions** (MDRS); **attention and frontal lobe functions** (mental control [from WMS], target detection tasks, TMT B, Stroop, subtests of the MDRS); **short-term verbal memory** (DS, immediate recall [from FACSRT]); **long-term verbal memory** (FACSRT, subtests of the MDRS); **visual memory** (BEM 144, CBT, subtests of the MDRS); **orientation** (orientation items of the MMSE); **EF** (subtests of the MDRS, WCST, verbal fluency); **language ability** (confrontation naming of 36 figures, and Token Test [SF]); **gestual praxis** (subtests of the MDRS, symbolic gesture, pantomiming of object use without objects); **gnosia** (identification of famous faces and naming of pictures of objects); **constructional and visuospatial functions** (subtests of the MDRS, and construction of the MMSE); **concept formation** (subtests of the MDRS, and WCST); and **reasoning** (calculation, arithmetic problem solving, evaluation of judgment by the criticism of verbal absurdities) | Visit with a neurologist or by telephone contact with the patient’s family or general practitioner | no raw data |
| 5 | Ozdemir et al. (Turkey) | 2001 | **Comparing stroke rehabilitation outcomes between acute inpatient and nonintense home settings** | yes | 60 | Multiple subtypes (hemorrhagic, thrombotic, lacunar, and embolic) | 3.5 | **Cognitive status** (MMSE) | Unblinded rehabilitation physician | non-parametric |
| 6 | Pohjasvaara et al. (Finland) | 2001 | **Suicidal ideas in stroke patients 3 and 15 months after stroke** | no | 286 | Ischemic | 15.0 | **Cognition** (MMSE) | Examination conducted by a board-certified neurologist | mean & SD |
| 7 | Bagg et al. (Canada) | 2002 | **Effect of age on functional outcomes after stroke rehabilitation** | no | 561 | First ischemic, hemorrhagic and SAH | 2.2 | **Cognition** (FIM) | Recorded by each patient’s treating therapists and primary nurse within 48 hours of admission and discharge | mean & SD |
| 8 | Bennet et al. (Australia) | 2002 | **Subcortical vascular disease and functional decline: A 6-year predictor study** | no | 77 | Ischemic | 71.7 | **Cognitive performance** (MMSE); [no area] (CDR); **attention and information processing** (DSF & DSB, and mental control [from WMS]); **language-confrontation naming** (naming 5 objects and naming 5 parts of object); **language-phonemic verbal fluency** (letters F-A-S); **language-auditory comprehension** (clinical observation); **spatial skills** (copy cross, clock drawing and house drawing); **memory** (LM [DR], and VR [from WMS], RAVLT); **EF** (CFST, similarities, and BDT [from WAIS-R], and Porteus Mazes); **behavioral change** (disorder of drive, disorder of control, and GDS) | Interviews with the participants and informants (n=36) | frequencies |
| 9 | Cavanagh et al. (USA) | 2002 | **Assessing cognitive function after stroke using the FIM instrument** | no | 441 | Ischemic stroke | 6.0 | **Cognitive function** (FIM) | Evaluation at rehab hospital | incomplete |
| 10 | Desmond et al. (USA) | 2002 | **Incidence of dementia after ischemic stroke: results of a longitudinal study** | no | 334 | Ischemic stroke | 120.0 | **Cognitive impairment** (MMSE) | Visited subjects’ homes or healthcare facilities if unable or unwilling to be examined in clinic. “Refusals” for a particular interval until had at least 4 attempts to contact and examine them. When in-person examinations were not possible, telephone information obtained | no raw data |
| 11 | Heruti et al. (Israel) | 2002 | **Rehabilitation outcome of elderly patients after a first stroke: effect of cognitive status at admission on the functional outcome** | no | 315 | First acute [not specified] | 1.7 | **Cognitive status** (FIM) | Patients are evaluated within 72 hours after admission by the rehabilitation team (composed of a nurse, a physiotherapist, an occupational therapist, a speech therapist | mean & SD |
| 12 | Lowery et al. (England) | 2002 | **Cognitive decline in a prospectively studied group of stroke survivors, with a particular emphasis on the > 75's** | no | 351 | Ischemic and hemorrhagic | 12.0 | Cognitive impairment (AMT and CDT) | NA | frequencies |
| 13 | Tham et al. (Singapore) | 2002 | **Progression of cognitive impairment after stroke: one year results from a longitudinal study of Singaporean stroke patients** | no | 252 | Ischemic and TIA | 15.0 | **[no area]** (MMSE); **attention** (DSB & DSF, VMSF & VMSB, and ADT); **language** (BNT [modified version], VFT); **verbal memory** (WLR [IR-DR & DRec], SR [IR & DR]); **visual memory** (PR [IR-DR & DRec], VR [IR-DR & DRec] [from WMS-R]); **visuoconstruction** (VR [Copy score] [from WMS-R], CDT, BDT [from WAIS-R]); **visuomotor speed** (DCT, DSMT, and Maze Task) | NA | incomplete |
| 14 | Berg et al. (Finland) | 2003 | **Poststroke depression: an 18-month follow-up** | no | 100 | First ischemic | 18.0 | **Intellectual abilities** (information, comprehension, similarities, arithmetic, BDT, picture arrangement, picture completion, and digit symbol [from WAIS]); **memory** (WMS-R); **motor functions** (hand sequencing, hand posture, hand reciprocal coordination [from Luria's tasks], and right & left hand tapping); **language** (WAB) | NA | no raw data |
| 15 | Bohannon et al. (USA) | 2003 | **Functional gains during acute hospitalization for stroke** | no | 451 | Acute ischemic | 0.3 | **Expression and memory** (FIM) | A trained research nurse gathered information about patients’ functional independence at admission and discharge by retrieving FIM scores | mean & SD |
| 16 | Fang et al. (China) | 2003 | **A study on additional early physiotherapy after stroke and factors affecting functional recovery** | yes | 360 | extract | 6.0 | **Cognitive status** (MMSE) | two trained neurologists blinded to the grouping allocation | mean & SD |
| 17 | Fruehwald et al. (Austria) | 2003 | **Early fluoxetine treatment of post-stroke depression - A three-month double-blind placebo-controlled study with an open-label long-term follow up** | yes | 54 | Ischemic and hemorrhagic | 18.0 | **Cognitive impairment** (MMSE) | Clinical eval performed by senior clinical psychiatrists | no raw data |
| 18 | Giaquinto and Fraoli (Italy) | 2003 | **Enhancement of the somatosensory N140 component during attentional training after stroke** | yes | 40 | Ischemic and hemorrhagic | 1.2 | **Disability** (FIM) | Rehabilitation setting | no raw data |
| 19 | Hochstenbach et al. (Netherlands) | 2003 | **Cognitive recovery after stroke: a 2-year follow-up** | no | 65 | Ischemic and hemorrhagic | 28.0 | **Orientation** (time, length of time, place, and person); **memory** (AVLT, DS [from WAIS-II], RBMT, and recounting a story); **attention** (TMT A & B, digit symbol [from WAIS-II], letter cancellation task, and picture scanning [from BIT]); **visuospatial and visuoconstuctive functions** (BDT [from WAIS-II], Bobertag test, structured clock test, CDT, copying task [from BIT], and Money's road map test); **language** (similarities subtest [from WAIS-II], writing task, and word comprehension, sentence comprehension, naming, and verbal fluency [animals] [from DAS]); **arithmetic** (handling money [recognizing-counting & arithmetic]) | All patients living at home at time of assessments | incomplete |
| 20 | Jorge et al. (Multinational) | 2003 | **Mortality and poststroke depression: a placebo-controlled trial of antidepressants** | yes | 104 | Ischemic or hemorrhagic | 24.0 | **Cognitive functioning** (MMSE) | Usually in the patient’s home or long-term care facility. Patients enrolled in Argentina had clinical follow-up at the Neuropsychiatry Outpatient Clinic of the Raúl Corea Institute of Neurological Research | incomplete |
| 21 | Kaneko et al. (Canada) | 2003 | **Relationship of sleep apnea to functional capacity and length of hospitalization following stroke** | no | 61 | Ischemic and hemorrhagic | 2.1 | **Cognitive function** (MMSE); **and social cognition** (FIM) | all patients underwent a series of tests administered by a research assistant to determine their level of functional and mental disability | no raw data |
| 22 | Narushima et al. (USA) | 2003 | **Does cognitive recovery after treatment of poststroke depression last? A 2-year follow-up of cognitive function associated with poststroke depression** | no | 59 | Ischemic or hemorrhagic | 24.0 | **Cognitive impairment** (MMSE) | Not specified in methods | incomplete |
| 23 | Riepe et al. (Germany) | 2003 | **Screening for cognitive impairment in patients with acute stroke** | no | 209 | Acute ischemic | 3.0 | **Orientation** (orientation [from ADAS-cog]); **naming** (aphasia scale [from ADAS-cog]); **memory** (MIS); **working memory and concentration** (letter sorting) | Not addressed | mean & SD |
| 24 | Tooth et al. (USA) | 2003 | **Effect of functional gain on satisfaction with medical rehabilitation after stroke** | no | 1967 | Ischemic, hemo, ICH, SAH, and unspecified | 3.7 | **Communication and social cognition** (FIM) | Inpatient rehabilitation and home living patients | incomplete |
| 25 | Tzourio et al. (Multinational) | 2003 | **Effects of blood pressure lowering with perindopril and indapamide therapy on dementia and cognitive decline in patients with cerebrovascular disease** | yes | 6105 | Ischemic, hemorrhagic, TIA and unknown | 54.8 | **Cognitive function** (MMSE) | Patient visits to 172 collaborating centres from 10 countries. 1049 were assessed “face to face” and the remaining 503 were assessed “in absentia" | frequencies |
| 26 | Altieri et al. (Italy) | 2004 | **Delayed poststroke dementia - A 4-year follow-up study** | no | 191 | Ischemic and hemorrhagic | 48.0 | **Memory** (AVLT, ROCF, and CBT); **attention** (Stroop); **Visuospatial functions** (ROCF); **EF** (TMT, and WCST); **conceptual thinking** (RCPM); **language** (COWAT, and Aachener Naming Subtest) | NA | no raw data |
| 27 | Appelros et al. (Sweden) | 2004 | **Recovery from unilateral neglect after right-hemisphere stroke** | no | 37 | First ever ischemic or hemorrhagic | 12.0 | **Cognitive function** (MMSE) | Not specified in methods | incomplete |
| 28 | Bakker et al. (Netherlands) | 2004 | **Cognition and quality of life in patients with carotid artery occlusion: a follow-up study** | no | 73 | TIA or ischemic stroke | 15.0 | **General intelligence** (RSPM and Vocabulary subtest [from WAIS-R]); **learning and memory** (WMS, VLMT); **EF** (TMT A & B, MCST, Word Production According to Lexical Rules-UNKA test); **reaction speed** (go-no-go test) | NA | incomplete |
| 29 | Cassidy et al. (Ireland) | 2004 | **Prevalence of post-stroke depression in an Irish sample and its relationship with disability and outcome following inpatient rehabilitation** | no | 50 | First stroke [CT or MRI] | 9.0 | **Cognition** (MMSE) | A Specialist Registrar in Rehabilitation Medicine carried out assessments of disability and cognition in the morning and a Liaison Psychiatry Research Fellow carried out the assessment of mood in the afternoon. The two assessors were blinded to each other’s findings | mean & SD |
| 30 | Greenberg et al. (Israel) | 2004 | **Post-stroke follow-up in a rehabilitation center outpatient clinic** | no | 120 | Ischemic and hemorrhagic | 16.8 | **Cognition** (FIM) | Ambulatory rehabilitation clinic | no raw data |
| 31 | Greenberg et al. (USA) | 2004 | **Hemorrhage burden predicts recurrent intracerebral hemorrhage after lobar hemorrhage** | no | 94 | ICH | 33.0 | **Cognitive impairment** (complete a list of daily cognitive tasks [informant reported]) | Systematic telephone interviews performed at 6 month intervals | frequencies |
| 32 | Keren et al. (Israel) | 2004 | **Relationship between rehabilitation therapies and outcome of stroke patients in Israel: a preliminary study** | no | 50 | First stroke | 3.4 | **Impairment** (MMSE and SIS); **cognitive Function** (FIM) | Acute rehabilitation setting | no raw data |
| 33 | Nas et al. (Turkey) | 2004 | **The relationship between physical impairment and disability during stroke rehabilitation: effect of cognitive status** | no | 40 | Ischemic, hemorrhagic and other | 16.4 | **Cognitive communicative skills** (FIM) | Physician certified by the Uniform Data System for Medical Rehabilitation. | mean & SD |
| 34 | Sacher et al. (Israel) | 2004 | **Role of disengagement failure and attentional gradient in unilateral spatial neglect - a longitudinal study** | no | 8 | First Ischemic or hemorrhagic | 2.5 | **Neglect** (BIT and LBT); **signal detection** (Starry Night Test); disengagement time (Spatial Cueing) | Not addressed | mean & SD |
| 35 | Sturm et al. (Germany) | 2004 | **Functional reorganisation in patients with right hemisphere stroke after training of alertness: a longitudinal PET and fMRI study in eight cases** | yes | 8 | Ischemic and vasculitis | 14.4 | **Intrinsic alertness, vigilance and visual scanning** (TBAP) | Not addressed | non-parametric |
| 36 | Akinwuntan et al. (Belgium) | 2005 | **Effect of simulator training on driving after stroke: a randomized controlled trial** | yes | 83 | First time ischemic or hemorrhagic | 4.8 | **Field of vision** (UFOV); **executive reasoning and attention** (SDSA) | Rehabilitation hospital, administered by neuropsychologists and driving assessment experts | mean & SD |
| 37 | Appelros et al. (Sweden) | 2005 | **Lacunar infarcts: Functional and cognitive outcomes at five years in relation to MRI findings** | no | 81 | First ever lacunar | 60.0 | **Cognitive function** (MMSE) | Assessed by a neurologist and an occupational therapist | non-parametric |
| 38 | Del Ser et al. (Spain) | 2005 | **Evolution of cognitive impairment after stroke and risk factors for delayed progression** | no | 193 | Ischemic and hemorrhagic | 24.0 | **Cognitive status** (SPMSQ) | Follow-up visits | mean & SD |
| 39 | Nys et al. (Netherlands) | 2005 | **The prognostic value of domain-specific cognitive abilities in acute first-ever stroke** | no | 243 | First-ever stroke (hemorrhagic or infarct) | 10.0 | **Reasoning** (RAPM [SF], and similarities [from WAIS-III]); **verbal memory** (RAVLT, DS [from WAIS-III], and SR [from WMS]); **EF** (BSAT, and VET [from TEA], and letter fluency); **visual perception and construction** (JLO [SF], TFR [SF], and ROCF [copy score]); **visual memory** (CBS and ROCF [delay score]); **language** (Token Test [SF], BNT [SF]); and **unilateral neglect** (star cancellation [from BIT]) | Three stroke units from three hospitals in the Netherlands (baseline); and follow-up visits | frequencies |
| 40 | O'Connor et al. (Ireland) | 2005 | **Late multidisciplinary rehabilitation in young people after stroke** | no | 50 | First ischemic or hemorrhagic (within 12 months post admission) | 10.4 | **Cognitive ability** (MMSE) | Research fellow in rehabilitation medicine carried out the assessments at the Hospital. | non-parametric |
| 41 | Ostir et al. (USA) | 2005 | **Functional status and satisfaction with community participation in persons with stroke following medical rehabilitation** | no | 1870 | Stroke (ICD Codes 4.36-4.39 - Cerebrovascular disease, no hemorrhage, no ICH, no SAH, and no TIA) | 4.0 | **Communication and social cognition** (FIM) | Follow-up information collected by nurses trained in functional assessments and telephone data collection methods | incomplete |
| 42 | Rameezan et al. (Malaysia) | 2005 | **Functional status of acute stroke patients in University Malaya Medical Centre (UMMC), Kuala Lumpur, Malaysia** | no | 51 | Ischemic (n=42), and hemorrhagic (n=5) [36 cases were first ever, and 15 recurrent] | 3.0 | **Cognitive impairment** (MMSE); and **functional status** (FIM) | Assessed by principal investigator (master in rehab medicine) | mean & SD |
| 43 | Rasquin et al. (Netherlands) | 2005 | **Cognitive performance after first ever stroke related to progression of vascular brain damage: a 2 year follow up CT scan study** | no | 189 | First ever ischemic or hemorrhagic | 24.0 | **Cognitive functioning** (MMSE and CAMCOG); **memory** (AVLT); **EF** (CST, and Stroop); **and calculation and visuospatial abilities** (GIT) | Outpatient clinic of University Hospital Maastricht (Jan 2000- Aug 2001) for baseline, and follow-up appointments | mean & SD |
| 44 | Tang et al. (China) | 2005 | **Effects of problem-oriented willed-movement therapy on motor abilities for people with poststroke cognitive deficits** | yes | 180 | First stroke [subtype not specified] | 4.0 | **Cognitive function** (MMSE) | Neurologist (admission) and after 8 weeks of therapy (not specified) | mean & SD |
| 45 | van Zandvoort et al. (Netherlands) | 2005 | **Early neuropsychological evaluation in patients with ischaemic stroke provides valid information** | no | 57 | First ever ischemic stroke | 24.0 | **Intellectual ability** (vocabulary [from WAIS], and RAPM); **language** (BNT, category fluency, and letter fluency); **memory** (DS [from WAIS], CBT, ROCF [delay score], RAVLT [IR-DR-DRec], and doors test); **attention** (TMT [sets A1-A2 & B]); **perception and visuospatial construction** (ROCF [copy score], TFP, and JLO) | Not specified in methods | frequencies |
| 46 | Olsson et al. (Sweden) | 2006 | **Effects of Day Hospital Rehabilitation After Stroke** | yes | 52 | First ever cerebral infarction (n=37), intracerebral bleeding (n=6), and subarachnoidal bleeding (n=9) (average time since onset 180 days, range 22-473 days) | 18.0 | **Cognitive function** (FIM) | Administered by accredited FIM educator | non-parametric |
| 47 | Pettersen et al. (Norway) | 2006 | **Prognostic significance of micturition disturbances after acute stroke** | no | 315 | Ischemic and hemorrhagic | 3.0 | **[no area]** (MMSE); **[no area]** (CDT); **EF** (TMT A) | NA | no raw data |
| 48 | Saxena et al. (Singapore) | 2006 | **Functional outcomes in inpatient rehabilitative care of stroke patients: predictive factors and the effect of therapy intensity** | no | 178 | Ischemic and hemorrhagic | 1.4 | **Cognitive impairment** (AMT) | Questionnaire interviews were performed by a trained research nurse, with translations for non-English- speaking patients. | no raw data |
| 49 | Simis et al. (Brazil) | 2006 | **Cognitive improvement after treatment of depressive symptoms in the acute phase of stroke** | yes | 93 | Acute supratentorial ischemic stroke | 3.0 | **Attention and memory** (DRS) | Visits completed by a blinded psychologist | mean & SD |
| 50 | Spalletta et al. (Italy) | 2006 | **Response of emotional unawareness after stroke to antidepressant treatment** | yes | 50 | First ever stroke [subtype not specified] | 7.0 | **Cognitive impairment** (MMSE) | Two clinical psychiatrists interviewed the patients. The same psychiatrists administered, at day zero and at weeks 1, 2, 4, 6, and 8 of treatment, | incomplete |
| 51 | van de Port et al. (Netherlands) | 2006 | **Susceptibility to deterioration of mobility long-term after stroke: a prospective cohort study** | no | 264 | First ischemic or hemorrhagic | 36.0 | **Cognitive status** (MMSE) | patients were visited by a trained research assistant for an assessment at home or at the institution where the patient resided | no raw data |
| 52 | Cardonnier et al. (France) | 2007 | **Early epileptic seizures after stroke are associated with increased risk of new-onset dementia** | no | 169 | Ischemic and hemorrhagic | 36.0 | **Global cognitive function** (MDRS) | Visit to a neurologist or by telephone contact | no raw data |
| 53 | Cherney and Halper (USA) | 2007 | **Performance on the California Verbal Learning Test following right hemisphere stroke: A longitudinal study** | no | 18 | Ischemic (n=14) and hemorrhagic (n=4) | 18.0 | **Learning and recall over time** (CVLT) | Administered by a trained research assistant. | mean & SD |
| 54 | Firbank et al. (UK) | 2007 | **Medial temporal atrophy rather than white matter hyperintensities predict cognitive decline in stroke survivors** | no | 108 | Stroke (multiple subtypes) | 27.0 | **Global cognition** (CAMCOG-R [section B only]); **dementia diagnostic instrument** (MMSE, and DSM IV criteria) | Not specified in methods | mean & SD |
| 55 | Fong et al. (China) | 2007 | **The effect of voluntary trunk rotation and half-field eye-patching for patients with unilateral neglect in stroke: a randomized controlled trial** | yes | 60 | First or second, ischemic or hemorrhagic stroke patients with unilateral neglect or inattention, right handed | 2.4 | **Cognitive performance** (MMSE); **unilateral neglect or visual inattention** (BIT) | RCT at rehab hospital in Hong Kong comparing voluntary trunk rotation (TR), TR plus half-eye patching, and controls; assessed by blinded assessors at follow-up at subjects' homes or other residence (i.e. institution) | no raw data |
| 56 | Hofgren et al. (Sweden) | 2007 | **Recovery after stroke: cognition, ADL function and return to work** | no | 58 | First ischemic or hemorrhagic stroke (not SAH) and < 65 YO | 12.0 | **Neuropsychological functions** (BNIS); and **cognition** (FIM) | Inpatient Rehabilitation Unit at University Hospital and follow-up visits | mean & SD |
| 57 | Hyndman et al. (UK) | 2007 | **The influence of attention deficits on functional recovery post stroke during the first 12 months after discharge from hospital** | no | 122 | Both infarction and hemorrhage (according to Oxford Community Stroke Project Classification) | 14.6 | **Attention** (TEA) | Not specified in methods | incomplete |
| 58 | Jutai et al. (Canada) | 2007 | **Mobility assistive device utilization in a prospective study of patients with first-ever stroke** | yes | 316 | First ever ischemic and hemorrhagic | 12.0 | **Cognitive impairment** (MMSE [brief version]) | Registered occupational or physical therapists (at baseline) while the person was still hospitalized; FU evaluations done over the telephone by therapists experienced in interviewing | non-parametric |
| 59 | Kulkantrakorn et al. (Thailand) | 2007 | **A prospective study in one year cumulative incidence of depression after ischemic stroke and Parkinson's disease: A preliminary study** | no | 77 | First time ischemic stroke | 12.0 | **Cognitive function** (MMSE [Thai version]) | face-to-face interview by experienced research worker and physician | no raw data |
| 60 | Lee and Yeh (Taiwan) | 2007 | **Correlation of common carotid artery intima media thickness, intracranial arterial stenosis and post-stroke cognitive impairment** | no | 30 | First time ischemic stroke | 3.0 | **Cognitive performance** (CASI) | Administered by a single investigator | mean & SD |
| 61 | Newman et al. (USA) | 2007 | **Association of diabetes, homocysteine, and HDL with cognition and disability after stroke** | yes | 3680 | Mild to moderate ischemic stroke | 26.0 | **Cognition** (MMSE) | 56 medical Centers in the USA, Canada, and Scotland (VISP trial). Intervention with pharmaceuticals. Baseline was evaluated at admission; follow-up visits followed. | mean & SD |
| 62 | Popovic et al. (Croatia) | 2007 | **Mild cognitive impairment in symptomatic and asymptomatic cerebrovascular disease** | no | 155 | First ischemic stroke or TIA | 6.0 | **Cognitive function** (MMSE and MoCA) | Baseline testing done during initial visit to outpatient's clinic | non-parametric |
| 63 | Pyoria et al. (Finland) | 2007 | **The effect of two physiotherapy approaches on physical and cognitive functions and independent coping at home in stroke rehabilitation. A preliminary follow-up study** | yes | 80 | Ischemic (33) and hemorrhagic (7) stroke in both groups. Previous stroke included (6 in active physio and 1 in traditional) | 12.0 | **Language** (Token Test, BNT, and Verbal Fluency Test); **visuospatial function** (CP, GCT, and ADAS [Four pictures]); **visual inattention** (LBT, and LCT); **memory** (verbal learning recall test [from WMS], and ADAS [recall of four pictures]) | Intervention context: 40 traditional physio and 40 activating physio. Evaluation completed in the neurological ward | non-parametric |
| 64 | Saxena et al. (Singapore) | 2007 | **Is improvement in impaired cognition and depressive symptoms in post-stroke patients associated with recovery in activities of daily living?** | no | 141 | Ischemic and hemorrhagic | 6.0 | **Cognitive impairment** (AMT) | Two rehab hospitals in Singapore (baseline); and residential place (own home, daycare centers, and nursing homes) at time of follow-up | frequencies |
| 65 | Seo & Oh (South Korea) | 2007 | **Acute physiologic predictors of mortality and functional and cognitive recovery in hemorrhagic stroke: 1-, 3-, and 6-month assessments** | no | 108 | Hemorrhagic and SAH | 6.0 | **Degree of cognitive abilities** (CAS) | Neurologic intensive care department for admission. Cognition measured directly or over the phone for those discharged | no raw data |
| 66 | Serrano et al. (Spain) | 2007 | **Frequency of cognitive impairment without dementia in patients with stroke - A two-year follow-up study** | no | 327 | Ischemic (88.4%) and hemorrhagic (11.6%) | 24.0 | **Cognitive status** (IQCODE-SS); **orientation** (temporal & spatial orientation [from MMSE]); **tonic attention** (hearing and simple visual reaction time); **phasic attention** (random visual reaction time); **verbal fluency** (category and phonetic fluency); **comprehension** (token test); **memory** (free immediate recall, free delayed recall, delayed logic memory); **and visuoconstructive ability** (BDT [from WAIS]) | Evaluation visits (administered by a neuropsychologist) - see Barba et al (2000) P-S dementia: clinical features and risk factors | mean & SD |
| 67 | Thajeb et al. (Taiwan) | 2007 | **Clinical analysis of first-ever acute ischemic stroke involving the territory of paramedian mesencephalic arteries** | no | 28 | First ever ischemic (PMAS) | 12.0 | **Cognitive function** (MMSE and MMT) | NA | no raw data |
| 68 | Townend et al. (Australia) | 2007 | **Longitudinal prevalence and determinants of early mood disorder post-stroke** | no | 125 | Ischemic and hemorrhagic | 3.0 | **Cognition** (MMSE) | Face to face interviews | mean & SD |
| 69 | Yang et al. (China) | 2007 | **Logistic regression analysis on risk factors for vascular dementia following cerebral infarction in 403 patients from Chongqing City. Hospital and family follow-up studies** | no | 546 | Ischemic | 3.3 | **Dementia diagnosis** (MMSE) | attending or higher-titled physicians from the Department of Neurology of our hospital to clinically diagnose the dementia on admission or at 3 months after admission | frequencies |
| 70 | Anderson et al. (Australia) | 2008 | **Individuals with first-ever clinical presentation of a lacunar infarction syndrome: Is there an increased likelihood of developing mild cognitive impairment in the first 12 months after stroke?** | no | 30 | First ever lacunar syndrome | 12.0 | **Mental status** (MMSE and KSNAP [Mental Status subtest]); **attention** (Bannatynes sequential index [developed from WAIS-R Arithmetic - DS & Digit Symbol]); **memory** (RBMT, ROCF [delay score], RAVLT total number of learnt words from A1-A5); **verbal processing** (KSNAP [four letter word subtest], Information and Similarities subtests [from WAIS-R], COWAT [total number of words]); **visuo-perceptual** (ROCF [copy score], BDT [from WAIS-R], KSNAP [Gestalt Closure subtest]); **[no area]** (CDT) | Study neuropsychologist, geriatrician and research nurse administered tests over three visits (over a 2-3 week period) in the participant's home | mean & SD |
| 71 | Denti et al. (Italy) | 2008 | **Outcome predictors of rehabilitation for first stroke in the elderly** | no | 359 | First ischemic, hemorrhagic or undetermined stroke | 1.7 | **Neuropsychological impairment** (MMSE); and **extent of disability** (FIM) | Not addressed | mean & SD |
| 72 | Diener et al. (Multinational) | 2008 | **Effects of aspirin plus extended-release dipyridamole versus clopidogrel and telmisartan on disability and cognitive function after recurrent stroke in patients with ischaemic stroke in the Prevention Regimen for Effectively Avoiding Second Strokes (PRoFESS) trial: a double-blind, active and placebo-controlled study** | yes | 20332 | Ischemic | 31.5 | **Cognitive function** (MMSE) | When patients were unable to attend a follow-up visit, scores were assigned by telephone assessment. For patients with severe aphasia, responses were obtained through a proxy or the treating physician | frequencies |
| 73 | Glymour et al. (USA) | 2008 | **Social ties and cognitive recovery after stroke: Does social integration promote cognitive resilience?** | no | 272 | Ischemic and Hemorrhagic | 6.0 | **Cognitive function** (MMSE); **attention** (DSF [from WAIS]); **repetition and comprehension** (BDAE); i**mmediate recall** (10-word list); d**elayed word recall** (11-min average delay); **[no area]** (1-min Animal Naming Test, TMT A & B) | Context of FIRST study – an RCT in 4 acute care and 4 rehab care hospitals testing efficacy of psychosocial intervention in stroke patients aged 45 or older. Intervention completed at patients’ home (1/week, 12 weeks, and tri-weekly for another 12 months); first evaluation performed ~13 days P-S, and 6 months post randomization | mean & SD |
| 74 | Lesniak et al. (UK) | 2008 | **Frequency and prognostic value of cognitive disorders in stroke patients** | no | 200 | First-ever ischemic or hemorrhagic stroke | 12.0 | **Orientation** (personal & temporal orientation); **attention** (reverse repetition: weekdays-months & 4 digits); **higher level perception** (visual gnosis, tactile gnosis, and finger gnosis); **memory-short term** (5-word repetition, figure recognition with 10s delay, and 5-word repetition with 3 min delay); **memory**-**long term** (date recollection [World War-II - first day of school year & current president]); **praxis** (limb imitations, oral imitations, and ideomotor commands); **visuospatial functions** (constructional ability, spatial attention, and calculation); **language** (spontaneous speech, repetition, naming, comprehension, reading, and writing); **EF** (go-no-go test [initiation & response-inhibition], verbal similarities [abstract thinking from WAIS], TMT [set-shifting], and VFT [generation]) | Stroke unit of the neurological department of the Institute of Psychiatry and Neurology; follow-up visit at 12 months | frequencies |
| 75 | Mok et al. (China) | 2008 | **A case-controlled study of cognitive progression in Chinese lacunar stroke patients** | no | 100 | Lacunar | 31.6 | **General cognitive function** (MMSE and ADAS-cog); **EF** (Initiation and perseveration [from MDRS]) | One of the authors from the Department of Medicine and Therapeutics) administered the cognitive tests at both assessments | mean & SD |
| 76 | Te Winkel-Witlox et al. (Netherlands) | 2008 | **Efficient screening of cognitive dysfunction in stroke patients: comparison between the CAMCOG and the R-CAMCOG, Mini Mental State Examination and Functional Independence Measure-cognition score** | no | 169 | First ever stroke (multiple types not specified) | 12.0 | **Cognitive dysfunction** (MMSE, CAMCOG, R-CAMCOG, and FIM) | Rehabilitation center on admission and home visit at 1 year by research assistants | mean & SD |
| 77 | Rabadi et al. (USA) | 2008.1 | **A pilot study of activity-based therapy in the arm motor recovery post stroke: a randomized controlled trial** | yes | 30 | First ischemic or Hemorrhagic | 2.8 | **Degree of disability** (FIM) | Recorded by certified therapist blinded to treatment allocation | mean & SD |
| 78 | Rabadi et al. (USA) | 2008.2 | **Intensive nutritional supplements can improve outcomes in stroke rehabilitation** | yes | 116 | First ischemic or hemorrhagic (within 4 weeks of admission to inpatient rehab) | 2.8 | **Outcome of rehabilitation** (FIM) | Trained, certified therapists | mean & SD |
| 79 | Barker-Collo et al. (New Zealand) | 2009 | **Reducing attention deficits after stroke using attention process training: a randomized controlled trial** | yes | 78 | Stroke (all pathological subtypes) | 6.6 | **Sustained, selective, divided, alternating attention** (Bells Test, IVA-CPT, TMT A & B, and PASAT [2 slowest trials]) | Administration and scoring were via standard procedures. Assessments took up to 2.5 hours and were well-tolerated, occurring over 2 sessions if required | non-parametric |
| 80 | Brioschi et al. (Switzerland) | 2009 | **Effect of Modafinil on Subjective Fatigue in Multiple Sclerosis and Stroke Patients** | yes | 20 | First ever stroke with minor neurological impairment (NIHSS 3 or less) | 28.0 | **Learning** (Rey Osterrieth [15 words IR & DR] [15 words adapted in French]); **EF** (Word fluency [phonemic & semantic], and Stroop animal test); **alertness and divided attention** (two computerized attention subtests [selected from the Test for Attentional Performance]) | Not addressed | no raw data |
| 81 | Bruandet et al. (France) | 2009 | **Alzheimer disease with cerebrovascular disease and vascular dementia: clinical features and course compared with Alzheimer disease** | no | 166 | Not specified | 100.8 | **Cognitive decline** (MMSE and DRS) | A multidisciplinary staff conducted a standardised examination including neurological, neuropsychological, behavioural, laboratory and imaging assessments for each patient | frequencies |
| 82 | Lee et al. (China) | 2009 | **Predictors of poststroke quality of life in older Chinese adults** | no | 214 | First ever ischemic | 6.0 | **Cognitive impact resulting from stroke** (AMT) | Research nurse at 1 month and 6 months after stroke onset at a rehabilitation unit or outpatient clinic during follow-up | mean & SD |
| 83 | Sachdev et al. (Australia) | 2009 | **The determinants and longitudinal course of post-stroke mild cognitive impairment** | no | 198 | Ischemic and TIA | 40.5 | **Verbal** **memory** (LM I & LM II [from WMS-R]); **visual memory** (VR I & VR II [from WMS-R]); **working memory** (DSB, Arithmetic [from WAIS-R]); **attention** (DS [from WAIS-R]); **mental control** (WMS-R); **language** (15-item BNT); **information processing speed** (TMT A and SDMT); **visuoconstruction** (BDT [from WAIS-R], and copying simple figures); **praxis-gnosis** (ideomotor apraxia subtest items, WAB [finger gnosis & stereognosis]); **abstract reasoning** (Similarities, Picture Completion [from WAIS-R]); **semantic fluency** (category of animals); **EF - Mental flexibility and verbal fluency** (CFST, TMT B, and phonemic fluency [letters F-A-S]); **premorbid ability** (NART-R) | Assessments performed by trained clinical psychologists. | incomplete |
| 84 | Winkens et al. (Netherlands) | 2009 | **Efficacy of Time Pressure Management in Stroke Patients With Slowed Information Processing: A Randomized Controlled Trial** | yes | 37 | Stroke (not specified) | 17.5 | **Performance in time pressure situations** (MSOT); **mental slowness in relation to daily activities** (MSQ) | Measurements were done by a research assistant (E.J.H.) who was unaware of the allocation of patients. Success of blinding was checked after- wards. | mean & SD |
| 85 | Cederfeldt et al. (Sweden) | 2010 | **Recovery in personal care related to cognitive impairment before and after stroke - a 1-year follow-up** | no | 74 | Ischemic or hemorrhagic | 12.0 | **Cognitive function** (MMSE); c**ognitive dysfunction** (CIMP-QUEST); **auditory memory** (WLM); **visual memory [nonverbal]** (CMT); **visuospatial function** (draw mirror image of a cup, count number of cubes, copy cube); **EF** (I-flex [a short form of the EXIT], and Stroop [Victoria version]); **speed and attention** (TMT A); **logical deductive ability** (RCM [set A]) | The same neuropsychologist measured cognitive functions with neuropsychological battery during the acute phase at the geriatric stroke unit and at 12 months after stroke onset at the policlinic (87%) or in the persons' home (13%) | frequencies |
| 86 | da Costa et al. (Brazil) | 2010 | **Cognitive evolution by MMSE in poststroke patients** | no | 42 | Ischemic (91%) | 6.0 | **Cognitive function** (MMSE) | All evaluation scales were carried out by two physiotherapists blinded to the results of the earlier application tests. | mean & SD |
| 87 | Delgado et al. (Chile) | 2010 | **Frequency and Determinants of Poststroke Cognitive Impairment at Three and Twelve Months in Chile** | no | 164 | Ischemic or hemorrhagic | 12.0 | **Global cognitive assessment** (MMSE); **attention, initiation and perseveration, construction, conceptualization and memory** (MDRS); **EF and processing speed** (FAB, 1-min phonemic verbal fluency [letters F-A-S], and 1-min semantic fluency tests with animals) | Assessment was performed by a blinded neuropsychologist | mean & SD |
| 88 | Farner et al. (Norway) | 2010 | **Depressive symptoms in stroke patients: A 13 month follow-up study of patients referred to a rehabilitation unit** | no | 194 | Ischemic or Hemorrhagic | 14.2 | **Cognitive impairment** (MMSE); g**eneral cognitive functioning, Immediate memory, visuospatial and constructional function, language, attention and delayed memory** (RBANS); **neglect and general visual attention** (SCT) | FU by authors at the clinic or patients' home | no raw data |
| 89 | Grau-Olivares et al. (Spain) | 2010 | **Progressive Gray Matter Atrophy in Lacunar Patients with Vascular Mild Cognitive Impairment** | no | 30 | First-ever stroke presenting clinical features of lacunar stroke | 18.0 | **Attention and short-term memory** (DSF and DSS [from WAIS-III]); **working memory** (DSB [from WAIS-III]); **premotor functions** (Lurias Premotor Sequences); **EF** (Categorical Verbal Fluency [animals-1-min], TMT A & B, and Stroop [interference subtest]); **memory** (VR [from WMS-III] OR delayed recall [from RAVLT]) | Not addressed | mean & SD |
| 90 | Hinkle (USA) | 2010 | **Outcome three years after motor stroke** | no | 100 | Ischemic (motor stroke) | 36.0 | **Cognitive status** (MMSE and COGNISTAT); and **function** (FIM) | Due to the advanced age of this population and the presence of neurological deficits affecting vision and strength, all instruments were read to the subjects. Total time for administration of the study instruments was approximately 1 hour. Breaks were taken during the interviews as needed to relieve subject fatigue | mean & SD |
| 91 | Kim et al. (Korea) | 2010 | **Effects of hypnotics on sleep patterns and functional recovery of patients with subacute stroke** | no | 30 | First ever ischemic or hemorrhagic | 1.1 | **Cognitive function** (MMSE and CNT [visual continuous performance test - auditory continuous performance test - DSF & DSB - visual span forward & backward tests for assessing **attention -** AVLT - & VRT for measurement of **memory function**]) | Examiners were blinded to the identity and symptoms of each patient | mean & SD |
| 92 | Perrier et al. (Canada) | 2010 | **Patient Factors Associated With Return to Driving Poststroke: Findings From a Multicenter Cohort Study** | no | 446 | Ischemic and hemorrhagic | 3.0 | **Cognition** (SIS memory and MMSE) | Evaluator administered | mean & SD |
| 93 | Quddus et al. (Bangladesh) | 2010 | **Assessment of stroke outcome based on initial stroke severity measured on different stroke severity scales** | no | 55 | Ischemic and hemorrhagic | 1.0 | **Functional status** (FIM) | Not specified in methods | incomplete |
| 94 | Rand et al. (Canada) | 2010 | **Feasibility of a 6-Month Exercise and Recreation Program to Improve Executive Functioning and Memory in Individuals With Chronic Stroke** | yes | 11 | Chronic stroke (ischemic and hemorrhagic) | 58.8 | **EF** - **response inhibition** (Stroop); **EF - attention and working memory** (DSB); **EF -** **psychomotor performance** (DST); **EF - visuomotor scanning, divided attention, and cognitive flexibility** (TMT B); **EF - attention switching** (WWT); **EF - learning, delayed recall, and long-term memory** (RAVLT) | The assessments were conducted in a quiet room in a research laboratory by trained examiners. | mean & SD |
| 95 | Sarkamo et al. (Finland) | 2010 | **Auditory and cognitive deficits associated with acquired amusia after stroke: a magnetoencephalography and neuropsychological follow-up study** | no | 53 | MCA territory ischemic stroke verified by MRI | 6.0 | **Working memory** (DS [from WMS-R] and Memory Interference); **verbal learning and memory** (ALL, and Story Recall [from RBMT]); **verbal expression and comprehension** (Repetition and reading [from BDAE], semantic fluency and naming [from CERAD], and Token Test [SF]); **visuospatial cognition** (Clock Task, Copying Designs, and BVRT [SF]); **music cognition** (MBEA-SF**); EF and attention** (FAB, Phonemic Test, Balloons Test, Simple Reaction Time, Subtraction Task, Stroop, and Vigilance Task) | All assessments were carried out in a quiet room reserved for clinical neuropsychological assessments. The 1-week P-S assessment was carried out in two or three testing sessions to avoid interference due to fatigue. On the average, the assessments were spread over 2.98 days (range 2–7 days). | mean & SD |
| 96 | Sholomov et al. (Russia) | 2010 | **The potential of transcranial magnetotherapy in color and rhythm therapy in the rehabilitation of ischemic stroke** | yes | 116 | First ischemic | 4.8 | **State of higher mental functions** (MMSE); **memory - short term and word memory** (Luriya's 10-word test); and **time to find increasing numbers** (Schulte test) | Not specified in methods | mean & SD |
| 97 | Vocat et al. (Switzerland) | 2010 | **Anosognosia for hemiplegia: a clinical-anatomical prospective study** | no | 50 | First ever ischemic or hemorrhagic | 6.0 | **General cognitive functioning** (MMSE [acute & chronic stages only]); **Unilateral visuospatial neglect** (Star Cancellation [from BIT], line bisection and copy of the Gainotti-Ogden figure, Reading of a short text of four lines); **personal neglect** (procedure of Bisiach); **mental flexibility** (VF - categorical [animals], VF - phonological [words]); self-monitoring of non-motor performance (Marcel et al. 2004 procedure); **mental flexibility and reasoning** (Weigl CST); **Short-term memory** (verbal span); **general long-term memory** (memorize three words [from MMSE]); **awareness of visuospatial neglect** (Catherine Bergego scale) | All tests were chosen to be given at the bedside and across a wide range of stroke severities | no raw data |
| 98 | Wagle et al. (Norway) | 2010 | **Cognitive impairment and the role of the ApoE epsilon4-allele after stroke--a 13 months follow-up study** | no | 104 | Ischemic and hemorrhagic | 13.0 | **Cognitive functioning** (MMSE and RBANS) | Evaluated by authors | non-parametric |
| 99 | Barret et al. (USA) | 2011 | **Enhancing Recovery After Acute Ischemic Stroke with Donepezil as an Adjuvant Therapy to Standard Medical Care: Results of a Phase IIa Clinical Trial** | yes | 33 | Ischemic | 15.0 | **[no area] (**MMSE, TMT A & B, COWAT, NPI-Q, IQCODE-SF) | Not addressed | mean & SD |
| 100 | Bouffioulx et al. (Belgium) | 2011 | **Satisfaction with activity and participation and its relationships with body functions, activities, or environmental factors in stroke patients.** | no | 45 | Acute CVA [subtype not defined] | 6.0 | **Cognitive function** (MMSE) | All participants assessed by the same examiner and included in the study. The patients received instructions on how to fill out the questionnaires and were tested individually in a quiet room. | non-parametric |
| 101 | Boy et al. (Germany) | 2011 | **Mobilisation of hematopoietic CD34+ precursor cells in patients with acute stroke is safe--results of an open-labeled non randomized phase I/II trial** | yes | 20 | First ischemic | 3.0 | **Long-term memory** (LM-I & LM-II, and ROCF [delay score]); **working memory** (DSB & DSF, and block span); **attention** (TMT B, Ruff 2 and Ruff 7); **lexical fluency** (COWAT); **semantic fluency** (animals); **visual perception** (ROCF [copy score], and TMT A) | NA | incomplete |
| 102 | Chang et al. (Korea) | 2011 | **Neural correlates of donepezil-induced cognitive improvement in patients with right hemisphere stroke: a pilot study** | yes | 14 | First-ever ischemic or hemorrhagic (including ICH) | 20.5 | **Cognitive function** (MMSE, ROCF, and SCNT) | Not specified in methods | mean & SD |
| 103 | Delbari et al. (Iran) | 2011 | **Effect of Methylphenidate and/or Levodopa Combined with Physiotherapy on Mood and Cognition after Stroke: A Randomized, Double-Blind, Placebo-Controlled Trial** | yes | 100 | Ischemic | 8.2 | **Cognitive function** (MMSE) | Not addressed | mean & SD |
| 104 | Hadidi et al. (USA) | 2011 | **Natural Patterns of Change in Poststroke Depressive Symptoms and Function** | no | 23 | First ischemic within the last 48 hours | 3.0 | **Function** (FIM); **cognitive mental status** (MMSE) | All instruments administered by principal investigator. Baseline and 2 weeks obtained in-person; last 2 assessments via telephone. | no raw data |
| 105 | Liman et al. (Germany) | 2011 | **Changes in Cognitive Function over 3 Years after First-Ever Stroke and Predictors of Cognitive Impairment and Long-Term Cognitive Stability: The Erlangen Stroke Project** | no | 705 | First ever stroke (ischemic or hemorrhagic) | 36.0 | **Global cognitive function** (MMSE) | Specially trained research nurses took interviews using standardized questionnaires | frequencies |
| 106 | Lin et al. (Taiwan) | 2011 | **Neurocognitive improvement after carotid artery stenting in patients with chronic internal carotid artery occlusion and cerebral ischemia** | yes | 20 | Ischemic ipsilateral hemisphere | 12.0 | **Global cognition** (MMSE); **memory, orientation, language, and ideational and constructional praxis** (ADAS-Cog); **verbal fluency** (category naming [fruits-vegetables & fish]); and **[no area]** (TMT A & B) | Performed by an independent clinical psychologist, who was blinded to the outcome of the intervention. | mean & SD |
| 107 | Markle-Reid et al. (Canada) | 2011 | **Interprofessional Stroke Rehabilitation for Stroke Survivors Using Home Care** | yes | 101 | Ischemic or TIA | 18.0 | **Cognitive function** (SPMSQ) | Interviews completed by independent health professionals, blinded to the purpose of the study and group assignment | mean & SD |
| 108 | Pendlebury et al. (England) | 2011 | **Transient Cognitive Impairment in TIA and Minor Stroke** | no | 280 | TIA or minor stroke (NIHSS 3 or less) | 60.0 | **Cognition** (MMSE) | Patients generally seen in outpatient setting on both initial and follow-up visits; none seen at home | mean & SD |
| 109 | Rengachari et al. (USA) | 2011 | **A behavioral analysis of spatial neglect and its recovery after stroke** | no | 30 | First ever right hemisphere stroke, ischemic or hemorrhagic | 8.7 | **Extinction** (double simultaneous stimulation of visual + auditory + tactile modalities); **visuospatial scanning** (BIT-star); **visuospatial scanning** (Mesulam shape cancelation); **functional neglect** (Baking Tray Test, and BIT-reading) | quiet dimmed testing room | mean & SD |
| 110 | Roosink et al. (Netherlands) | 2011 | **Persistent shoulder pain in the first 6 months after stroke: results of a prospective cohort study** | no | 31 | First Ischemic | 6.0 | **Cognitive function** (MMSE) | Assessment at t0 was performed during the hospital stay, whereas assessments at t1 and t2 were performed at a regional rehabilitation clinic or, if the patient had no transportation, at the patient’s residence | frequencies |
| 111 | Tang et al. (China) | 2011 | **Absence of cerebral microbleeds predicts reversion of vascular 'cognitive impairment no dementia' in stroke** | no | 47 | Ischemic | 15.0 | **Global cognitive function** (MMSE); **EF** (FAB); **attention** (DSB & DSF, and ADT); **language** (BNT [modified version], verbal fluency [animal & food categories]); **verbal memory** (WLR [IR-DR & DRec], and story recall [IR & DR]); **visual memory** (PR [IR-DR & DRec], VR [I - II & DRec] [from WMS-III]); **visuoconstruction** (CDT, BDT [from WAIS-III], and VR copy [from WMS-III]); **visuomotor speed** (SDMT, DCT, and maze task) | A neurologist and a research assistant administered neuropsychological tests in the form of the modified Vascular Dementia Battery | no raw data |
| 112 | Viscogliosi et al. (Canada) | 2011 | **Participation after a stroke: changes over time as a function of cognitive deficits** | no | 197 | Ischemic and hemorrhagic | 6.0 | **Episodic memory** (LM [from WMS-III]); **visual perception** (MVPT-V); **EF - inhibition** (stroop [Victoria version]); unilateral visual neglect (Bells test); **language-picture naming** (BNT); **language-sentence comprehension** (Token Test [SF]); **language-reading capacity** (Montreal-Toulouse reading test) | Assessments at home by an occupational therapist | no raw data |
| 113 | Wang et al. (USA) | 2011 | **Time to inpatient rehabilitation hospital admission and functional outcomes of stroke patients** | no | 1908 | Multiple subtypes (ischemic, ICH, SAH, and undefined) | 1.6 | **Cognition** (FIM) | Inpatient rehabilitation | mean & SD |
| 114 | Whiting et al. (Australia) | 2011 | **Predictors for 5-year survival in a prospective cohort of elderly stroke patients** | no | 186 | Ischemic and hemorrhagic | 60.0 | **Cognitive function** (FIM) | Administered by an investigator or their relative or carer if the patient was unable to communicate | mean & SD |
| 115 | Chaiyawat and Kulkantrakorn (Thailand) | 2012 | **Randomized controlled trial of home rehabilitation for patients with ischemic stroke: impact upon disability and elderly depression** | yes | 60 | Ischemic (MCA infarction) | 24.0 | **Dementia** (MMSE [Thai Version]) | All planned FU visits at patients’ residences. Telephone interviews for patients outside of study area | mean & SD |
| 116 | Gregoire et al. (England) | 2012 | **Cerebral Microbleeds and Long-Term Cognitive Outcome: Longitudinal Cohort Study of Stroke Clinic Patients** | no | 55 | Ischemic and TIA | 68.0 | **General intellectual functioning** (WAIS-R or RCPM)**; verbal and visual memory functions** (RMT)**; naming skills** (GNT or ONT)**; perceptual functions** (VOSPB); **speed and attention** (Letter Cancellation, Digit Copy, SDMT, or TMT A); **EF** (Stroop, Word Fluency, TMT B, WCFST, and MCST) | Neuropsychologist blinded to clinical details and MRI data at the time of assessing | frequencies |
| 117 | Kettunen et al. (Finland) | 2012 | **Recovery From Visual Neglect After Right Hemisphere Stroke: Does Starting Point in Cancellation Tasks Change After 6 Months?** | no | 43 | First ischemic stroke | 6.0 | **Visual neglect** (BIT) | Acute setting | mean & SD |
| 118 | Kim (Korea) | 2012 | **Effects of an Enjoyable Nurse-Led Intervention to Promote Movement in Poststroke Inpatients** | yes | 45 | Stroke patients (type undefined) | 4.7 | **Functional status** (FIM) | Blindly measured by physicians | mean & SD |
| 119 | Koh et al. (Singapore) | 2012 | **Effect of duration, participation rate, and supervision during community rehabilitation on functional outcomes in the first poststroke year in Singapore** | no | 215 | Ischemic and hemorrhagic | 12.0 | **Cognitive impairment** (AMT) | Interviews and measurements were administered by 3 research nurses who were trained | no raw data |
| 120 | Liman et al. (Germany) | 2012 | **Impact of low mini-mental status on health outcome up to 5 years after stroke: the Erlangen Stroke Project** | no | 1631 | First ever Ischemic, primary ICH, SAH and undefined type of stroke | 60.0 | **Global cognitive function** (MMSE) | NA | frequencies |
| 121 | Mok et al. (China) | 2012 | **Predictors for cognitive decline in patients with confluent white matter hyperintensities** | no | 61 | Ischemic | 24.0 | **General cognition (MMSE); EF** (MDRS [initiation-perseveration]); and dementia (CDR) | An experienced psychologist administered the CDR (other instruments not specified) | no raw data |
| 122 | Pahlman et al. (Sweden) | 2012 | **Cognitive Dysfunction and Physical Activity After Stroke: The Gothenburg Cognitive Stroke Study in the Elderly** | no | 74 | Stroke (WHO definition) | 12.0 | **Global cognition** (CIMP-QUEST**); Speed and attention** [psychomotor speed] (TMT A, Stroop Test 1 & 2 [Victoria version]); a**uditory memory** (WLM); n**on-verbal visual memory** (CMT); v**isuospatial function** (draw mirror image of a cup, count number of cubes, and copy a cube); **higher visual perception** (VOSP [Silhouettes subtest]); l**anguage** [judged by neuropsychologist] (spontaneous speech fluency, auditory comprehension, anomia, verbal & literal paraphasia, reading & writing capacity); **EF** (I-Flex, Stroop [Victoria version]); **logical deductive ability** (RCPM [set A]); **gnosia** (Visual interpretation of pictures & objects, visual recognition of photographs of well-known faces & colors); **sensory and visual neglect** (simultaneous stimulation of both hands, double simultaneous stimulation of both visual fields, and LBT); **and praxia** (cut a paper with a pair of scissors, handle a matchbox, and strike a match) | All patients were assessed by a neuropsychologist | frequencies |
| 123 | Radman et al. (Switzerland) | 2012 | **Poststroke fatigue following minor infarcts: a prospective study** | no | 109 | First ischemic or hemorrhagic "nondisabling" stroke (< 7 days of onset and < 7 NIHSS) | 12.0 | **Attention-**phasic alert and divided attention (TEA, and D2); **language** - **object naming from line drawing** (BNT [French version]); **written comprehension** (BDAE); **EF** (Stroop [modified version]); **category and letter fluency tasks, and nonverbal directed fluency task** ([test not specified]); memory-s**hort-term verbal and nonverbal memory** (DS and CBT); memory-l**ong-term memory** (RAVMT) | A neuropsychologist conducted a detailed cognitive examination | frequencies |
| 124 | Allan et al. (England) | 2013 | **Long-term incidence of depression and predictors of depressive symptoms in older stroke survivors** | no | 355 | Ischemic (OCSP scale) | 120.0 | **Global cognitive performance** (CAMCOG) | Not addressed | no raw data |
| 125 | Andersson et al. (Sweden) | 2013 | **Hip fractures in persons with stroke** | no | 377 | First ischemic or hemorrhagic | 24.0 | **Cognitive function** (MMSE) | Stroke unit during acute stay | no raw data |
| 126 | Chan et al. (China) | 2013 | **Unilateral Neglect in Stroke A Comparative Study** | yes | 40 | Not specified | 1.5 | **General cognitive functions** (MMSE); **unilateral neglect** (BIT-conventional subtest); **visual fields** (Visual Confrontation Test) | Subjects were tested and trained on a one-to-one basis during their normal daily therapy sessions | no raw data |
| 127 | Dong et al. (Australia) | 2013 | **Ischaemic stroke: the ocular motor system as a sensitive marker for motor and cognitive recovery** | no | 25 | First ever ischemic | 3.0 | **Cognitive function** (MMSE, Depression Anxiety Stress Scale, and DSB) | Not addressed | incomplete |
| 128 | Duoiri et al. (England) | 2013 | **Prevalence of Poststroke Cognitive Impairment South London Stroke Register 1995-2010** | no | 4212 | First ever (multiple subtypes) TOAST criteria | 180.0 | **Cognitive state** (MMSE and AMT) | Special trained study nurses and field workers collected all data | frequencies |
| 129 | El Senousy et al. (Egypt) | 2013 | **Leukoaraiosis as a predictor of short term outcome of acute Ischemic stroke** | no | 45 | First ever ischemic | 6.0 | **Cognitive function** (MoCA) | Not specified in methods | frequencies |
| 130 | Kang et al. (South Korea) | 2013 | **White matter hyperintensities and functional outcomes at 2 weeks and 1 year after stroke** | no | 408 | Ischemic | 12.0 | **Cognitive function** (MMSE) | Not addressed | incomplete |
| 131 | Ku et al. (Taiwan) | 2013 | **Association Between Cerebral Lesions and Emotional Changes in Acute Ischemic Stroke Patients** | no | 26 | Ischemic stroke | 1.0 | **Cognitive function** (MMSE) | performed by a clinical psychologist | mean & SD |
| 132 | Mikami et al. (USA) | 2013 | **Incident Apathy During the First Year After Stroke and Its Effect on Physical and Cognitive Recovery** | no | 56 | Ischemic or hemorrhagic | 15.0 | **Global cognitive impairment** (MMSE) | Not specified in methods | mean & SD |
| 133 | Ng et al. (Singapore) | 2013 | **Functional outcomes after inpatient rehabilitation in a prospective stroke cohort** | no | 1332 | Ischemic or hemorrhagic | 0.9 | **Communication, social interaction, problem-solving, and memory** (FIM) | A multidisciplinary team led by a rehabilitation physician assesses and scores the FIM for all stroke rehabilitation inpatients | mean & SD |
| 134 | Paul et al. (India) | 2013 | **Depression Among Stroke Survivors: A Community-based, Prospective Study from Kolkata, India** | no | 241 | Ischemic or hemorrhagic | 93.0 | **Cognition** (MMSE [Bengali Version]) | Door-to-door interviews | mean & SD |
| 135 | Prokopenko et al. (Russia) | 2013 | **Correction of post-stroke cognitive impairments using computer programs** | yes | 43 | Hemisphere stroke | 15.0 | **Cognitive status** (MMSE); **cognitive disorders** (FAB); **optical-spatial gnosis and EF** (CDT); **moderate cognitive dysfunction** (MoCA); **concentration and switching of attention** (Schulte's tables) | Trained assessor blind to randomization | non-parametric |
| 136 | Rachpukdee et al. (Thailand) | 2013 | **Quality of Life of Stroke Survivors: A 3-Month Follow-up Study** | no | 125 | Ischemic or hemorrhagic (including ICH and SAH) | 3.0 | **Neurologic function** (CNS) | Hospital and proxy | mean & SD |
| 137 | Smania et al. (Italy) | 2013 | **Factors Predicting Functional and Cognitive Recovery Following Severe Traumatic, Anoxic, and Cerebrovascular Brain Damage** | no | 104 | Ischemic, hemorrhagic and SAH | 2.8 | **Cognitive and behavioral improvement** (LCF test) | Assessments recorded upon admission to and discharge from the rehabilitation unit | mean & SD |
| 138 | Suzuki et al. (Japan) | 2013 | **Predicting Recovery of Cognitive Function Soon after Stroke: Differential Modeling of Logarithmic and Linear Regression** | no | 57 | Ischemic and hemorrhagic | 1.0 | **Global cognition** (MMSE) | Evaluations performed in the context of treatment with PT and OT | non-parametric |
| 139 | Yang et al. (Singapore) | 2013 | **Level and predictors of participation in patients with stroke undergoing inpatient rehabilitation** | no | 122 | First ischemic or hemorrhagic | 0.9 | **Cognition** (ECAQ) | Inpatient rehabilitation | no raw data |
| 140 | Zhang et al. (China) | 2013 | **Prophylactic Effects of Duloxetine on Post-Stroke Depression Symptoms: An Open Single-Blind Trial** | yes | 118 | First time ischemic | 9.0 | **Cognitive function** (MMSE) | Administered by a neurologist blinded to randomization procedures | mean & SD |
| 141 | Aben et al. (Netherlands) | 2014 | **Long-lasting effects of a new memory self-efficacy training for stroke patients: a randomized controlled trial** | yes | 153 | First time ischemic (67%), left-sided stroke (57%) | 66.0 | **Memory self-efficacy** (Memory in Adulthood Questionnaire) | Home environment | incomplete |
| 142 | Boe et al. (Denmark) | 2014 | **Cognitive status does not predict motor gain from post stroke constraint-induced movement therapy** | yes | 21 | Ischemic or hemorrhagic | 9.0 | **Processing speed** (Symbol Search, and TMT A); **attention and working memory** (DS and Spatial Span [from WMS-III]); **learning and memory (**AVLT, RAVLT [DR], LLT [displacement score-modified version]); verbal ability (BNT); **visuospatial construction** (BDT [from WAIS-III]); **EF** (TMT B, phonemic fluency [letter s], ToL) | Tests administered in the same order at every session by an experienced neuropsychologist or by a student in psychology supervised by the former | non-parametric |
| 143 | Chau et al. (China) | 2014 | **Can short-term residential care for stroke rehabilitation help to reduce the institutionalization of stroke survivors?** | yes | 155 | Ischemic or hemorrhagic | 14.0 | **Cognitive status** (MMSE) | Interviews were conducted by trained research assistants using structured questionnaires | non-parametric |
| 144 | Chaudhari et al. (India) | 2014 | **Clinico-radiological predictors of vascular cognitive impairment (VCI) in patients with stroke: a prospective observational study** | no | 102 | Ischemic or hemorrhagic | 6.0 | **Cognition** (MMSE and SPMSQ) | Not specified in methods | non-parametric |
| 145 | Dacosta-Aguayo et al. (Spain) | 2014 | **Structural Integrity of the Contralesional Hemisphere Predicts Cognitive Impairment in Ischemic Stroke at Three Months** | no | 14 | First ischemic | 3.0 | **Global cognition** (MMSE); **sustained attention** (DSF [WAIS-III-R], MoCA [attention subtest], LCT); **EF and working memory** (DSB [from WAIS-III-R], TMT B); **phonological fluency** (letter fluency [words with P]); semantic fluency (animals); **language** (interview, and BNT); **premotor abilities** (Luria's sequences, rythms subtest [from MoCA], FAB); **speed and visuomotor coordination** (TMT A and GPT) | Acute examination performed in a fixed order that took approximately 60 minutes to complete. If patient fatigued, testing was split between two sessions carried out in the same day. FU eval lasted 2 hours. Information about previous cognitive impairment by a trained neuropsychologist | no raw data |
| 146 | De Ryck et al. (Belgium) | 2014 | **Poststroke depression and its multifactorial nature: Results from a prospective longitudinal study** | no | 222 | Ischemic, hemorrhagic, and TIA | 18.0 | **Cognitive function** (MMSE, FIM and SIS) | All patients were assessed by trained interviewers within seven days of stroke onset and re-evaluated at 1, 3, 6, 12 and 18 months P-S | incomplete |
| 147 | Ding et al. (China) | 2014 | **Patterns in default-mode network connectivity for determining outcomes in cognitive function in acute stroke patients** | no | 20 | First-ever ischemic | 3.0 | **Cognitive function** (MMSE and MoCA) | Not specified in methods | mean & SD |
| 148 | Dundar et al. (Turkey) | 2014 | **A comparative study of conventional physiotherapy versus robotic training combined with physiotherapy in patients with stroke** | no | 107 | First ischemic and hemorrhagic | 10.4 | **Cognitive function** (MMSE) | Not addressed | mean & SD |
| 149 | El Hachioui et al. (Netherlands) | 2014 | **Nonlinguistic cognitive impairment in poststroke aphasia: A prospective study** | no | 147 | First ischemic or hemorrhagic | 12.0 | **Aphasia** (ScreeLing and Token Test); **abstract reasoning** (Matrix reasoning [from WAIS-III], and VSAT); **visual memory** (direct recall and DR [from WMS-III], and SRMT-Faces [from CMT]); **visual perception and construction** (BCT, BDT [from WAIS-III], and CDT); **and EF** (WCST, TMT A & B, and Weigl Sorting Test) | FU assessments were conducted in the subsequent setting, that is, nursing home, rehabilitation center, or at home | frequencies |
| 150 | Ihle-Hansen et al. (Norway) | 2014 | **Multifactorial vascular risk factor intervention to prevent cognitive impairment after stroke and TIA: a 12-month randomized controlled trial** | yes | 195 | First ever ischemic, hemorrhagic or TIA | 12.0 | **Focused visual attention and information processing** (TMT A); and **verbal memory** (10-word test); **EF** (TMT B) | Not specified in methods | mean & SD |
| 151 | Kim et al. (Korea) | 2014 | **Effect of Dual-task Rehabilitative Training on Cognitive and Motor Function of Stroke Patients** | yes | 20 | Stroke patients (subtype not specified) with hemiparalysis | 18.5 | **Cognitive abilities** (Stroop) | Not specified in methods | mean & SD |
| 152 | Motta et al. (USA) | 2014 | **Diffusion-perfusion mismatch: An opportunity for improvement in cortical function** | no | 38 | Acute ischemic | 0.3 | **Neglect** (LCT - for right hemisphere stroke); **and [no area]** (Picture naming test - for left hemisphere stroke) | NA | non-parametric |
| 153 | Ntsiea et al. (South Africa) | 2014 | **The effect of a workplace intervention programme on return to work after stroke: a randomised controlled trial** | yes | 80 | Stroke (not specified) | 7.2 | **Global cognition** (MoCA) | Researchers who assessed outcomes at three and six months remained blinded to participant allocation throughout the study | mean & SD |
| 154 | Park et al. (South Korea) | 2014 | **Significance of longitudinal changes in the default-mode network for cognitive recovery after stroke** | no | 180 | First ischemic or hemorrhagic | 6.0 | **Global cognitive function** (MMSE); **attention, verbal memory, nonverbal memory and visuo-motor coordination** (SCNT) | Not addressed | mean & SD |
| 155 | Penaloza et al. (Spain) | 2014 | **Language recovery and evidence of residual deficits after nonthalamic subcortical stroke: A 1year follow-up study** | no | 59 | First subcortical nonthalamic stroke (ischemic or hemorrhagic) | 12.0 | **Language abilities -** **speech fluency and information content in spontaneous speech** (Conversational Narrative Speech Test); **narrative speech** (Picture description Test); **word generation** (Animal Naming Fluency Test); **Auditory comprehension** (Commands Test); **verbal short term memory** (Auditory Digit forward test); and **verbal comprehension of complex material** (Token Test) | Not addressed | mean & SD |
| 156 | Pihlaja et al. (Finland) | 2014 | **Post-stroke fatigue is associated with impaired processing speed and memory functions in first-ever stroke patients** | no | 133 | First ever supratentorial ischemic stroke | 24.0 | **Processing speed** (TMT A, Stroop [color naming], and Digit Symbol [coding subtest from WAIS-III]); **memory** (LM I [from WMS-R], 10-word list learning task, and BVRT-SF); **EF** (TMT [set B minus A], stroop [interference minus naming], and phonemic fluency task); **reasoning** (similarities and BDT [from WAIS-III]) | Not specified in methods | mean & SD |
| 157 | Xiong et al. (China) | 2014 | **Prestroke Statins, Progression of White Matter Hyperintensities, and Cognitive Decline in Stroke Patients with Confluent White Matter Hyperintensities** | no | 81 | Ischemic | 24.0 | **Global cognition** (MMSE); and **EF** (MDRS [initiation-perseveration]) | Not specified in methods | non-parametric |
| 158 | Ben Assayag et al. (Israel) | 2015 | **Gait Measures as Predictors of Poststroke Cognitive Function Evidence From the TABASCO Study** | no | 298 | First-ever ischemic stroke or TIA | 24.0 | **[no area]** (MoCA); **memory, executive functions, visuospatial perception, verbal function and attention** (NTCCTB) | Assessments reviewed by consensus forum including the assessor [not specified], 3 senior neurologists specializing in memory disorders, and a neuropsychologist | mean & SD |
| 159 | Brey et al. (USA) | 2015 | **Socioeconomic disparities in work performance following mild stroke** | no | 21 | Mild stroke (NIHSS LT 6) | 7.0 | **Alertness, orientation, attention and short-term memory** (SBT); **verbal working memory** (DSF & DSB [from WMS]); **visual scanning patterns, number sequencing, letter sequencing, number-letter switching and motor speed** (TMT); **verbal memory** (CVLT-II); **item omissions, commissions** [number of times person responds to a non-target item], **hit reaction time, attentiveness, perseveration, vigilance and adjustment to presentation speed** (CCPT); **general intelligence** (WTAR) | Three-hour visit in research laboratory. | no raw data |
| 160 | Burke Quinlan et al. (USA) | 2015 | **Neural function, injury, and stroke subtype predict treatment gains after stroke** | yes | 29 | Ischemic and hemorrhagic | 6.1 | **Cognition** (MMSE) | A single rater performed all behavioral assessments | no raw data |
| 161 | Gregorovich et al. (Canada) | 2015 | **Restricted Participation in Stroke Caregivers: Who Is at Risk?** | no | 399 | First ischemic or hemorrhagic | 24.0 | **Global cognitive status** (MMSE and SIS) | Data were collected by phone with trained research assistants during structured, 1-hour long interviews | no raw data |
| 162 | Horstmann et al. (Germany) | 2015 | **Cognitive Impairment Is Not a Predictor of Failure to Adhere to Anticoagulation of Stroke Patients with Atrial Fibrillation** | no | 990 | Ischemic stroke or TIA | 12.0 | **Cognitive performance** (MoCA) | Not specified in methods | non-parametric |
| 163 | Kauranen et al. (Finland) | 2015 | **Use of Stroke-Related Income Supplements and Predictors of Use in a Working-Aged Finnish Ischemic Stroke Cohort** | no | 230 | First-ever ischemic | 24.8 | **EF** (TMT B, go-no-go task, and phonemic fluency); **psychomotor speed** (TMT A, time of copying task, and Token Test [modified version-time to complete]); **episodic memory** (LM II [from WMS-R], learning a series of 10 unrelated words, and modified BVRT); **working memory** (DS [from WAIS - III], homogeneous interference task, and heterogeneous interference task); **language** (MTT, VNT, and repetition of a long sentence); **visual-spatial and construction skills** (copying 4 geometric figures, clock arms test with 10 clocks, and visuospatial searching task); and **motor skills** (bimanual movements task, fist-edge-palm task, and finger-tapping test) | Administered by experienced clinical neuropsychologists following a written study protocol | frequencies |
| 164 | Kumral et al. (Turkey) | 2015 | **Cognitive Decline in Patients with Leukoaraiosis Within 5 Years after Initial Stroke** | no | 8784 | Ischemic, TIA, hemorrhagic and undefined | 60.0 | **Global cognitive function** (MMSE) | NA | frequencies |
| 165 | Lawrence et al. (England) | 2015 | **Pattern and Rate of Cognitive Decline in Cerebral Small Vessel Disease: A Prospective Study** | no | 121 | Lacunar stroke patients | 36.0 | **Working Memory** (DSB & DSF); **long term memory** (LM and VR); **processing speed** (BMIPB SOIP, Digit Symbol, and GPT); **EF** (TMT B, Verbal Fluency [letters F-A-S & B-H-R], and WCST [modified version]); **global cognition** (all tests combined) | Not specified in methods | no raw data |
| 166 | Lee et al. (Korea) | 2015 | **Six-month functional recovery of stroke patients: a multi-time-point study** | no | 29 | First ever ischemic or hemorrhagic stoke | 6.0 | **Recovery of cognition** (MMSE) | All patients were screened by one physiotherapist | mean & SD |
| 167 | Liu-Ambrose et al. (Canada) | 2015 | **Exercise training and recreational activities to promote executive functions in chronic stroke: A proof-of-concept study** | yes | 25 | Ischemic or hemorrhagic | 30.0 | **Selective attention and conflict resolution** (Stroop); **set shifting** (TMT A & B); **working memory** (verbal digits forward & backward) | Not specified in methods | mean & SD |
| 168 | Lu et al. (China) | 2015 | **Impact of repetitive transcranial magnetic stimulation on post-stroke dysmnesia and the role of BDNF Val66Met SNP** | yes | 50 | First ever ischemic or hemorrhagic | 3.0 | **Short-term memory recall, visuospatial abilities, executive functions, attention, concentration, working memory, language, and orientation to time & space** (MoCA); **orientation, perception, visual movement organization, thought operation, attention and concentration** (LOTCA); **everyday memory problems** (RBMT) | Assessment done associate chief physician from the rehabilitation division. Accuracy of results verified by the chief physician | no raw data |
| 169 | Mehrabian et al. (Bulgaria) | 2015 | **Neuropsychological and neuroimaging markers in prediction of cognitive impairment after ischemic stroke: A prospective follow-up study** | no | 85 | First ever ischemic stroke | 12.0 | **Global cognitive functioning** (MMSE); **learning and episodic verbal memory** (WLL [IR & DR from CERAD], and recognition); **attention** (TMT A); **EF -** cognitive flexibility and set shifting (TMT B); EF - verbal fluency and set shifting (IST [15-second version]); **language abilities** (15-item subset of the BNT); **constructive praxis** (figure copying [from CERAD]) | Not specified in methods | mean & SD |
| 170 | Narasimhalu et al. (Singapore) | 2015 | **Severity of CIND and MCI predict incidence of dementia in an ischemic stroke cohort** | no | 362 | Ischemic and TIA | 63.0 | **Attention** (DS, Visual Span, and auditory detection); **language** (BNT [modified version] and category fluency [animals & food subtasks]); v**isuomotor speed** (SDMT, Digit Cancellation, and Maze Task); v**isuoconstruction** (VR copy task [from WMS-R], Clock Drawing, and BDT [from WAIS-R]); v**erbal memory** (WLR [IR-DR & DRec] and Story Recall [IR & DR]); v**isual memory** (PR [IR-DR & DRec] and VR [IR-DR & DRec] [from WMS-R]) | Trained research psychologists administered a neuropsychological test battery that has previously been validated for use in Singapore | frequencies |
| 171 | Persson et al. (Sweden) | 2015 | **Spouses of Stroke Survivors Report Reduced Health-Related Quality of Life Even in Long-Term Follow-Up Results From Sahlgrenska Academy Study on Ischemic Stroke** | no | 248 | Ischemic | 84.0 | **Cognitive impairment** (MMSE) | Research nurse (face to face) or by telephone. MMSE scored by research physician | no raw data |
| 172 | Qu et al. (China) | 2015 | **Improved visual, acoustic, and neurocognitive functions after carotid endarterectomy in patients with minor stroke from severe carotid stenosis** | no | 80 | Minor stroke (NIHSS 5 or less) | 4.5 | **Cognitive Impairment** (MMSE) | These examinations were conducted by independent physicians of relevant specialties who were not involved in the operation or in the administration of medication | mean & SD |
| 173 | Rode et al. (France) | 2015 | **Long-term sensorimotor and therapeutical effects of a mild regime of prism adaptation in spatial neglect. A double-blind RCT essay** | yes | 18 | Ischemic and hemorrhagic | 1.7 | **Therapeutic efficacy** (FIM); **Spatial neglect severity** (BIT) | Researchers carried out evaluations | no raw data |
| 174 | Song et al. (China) | 2015 | **Background Rhythm Frequency and Theta Power of Quantitative EEG Analysis: Predictive Biomarkers for Cognitive Impairment Post-Cerebral Infarcts** | no | 105 | Ischemic | 1.0 | **Cognitive impairment** (MoCA) | NA | frequencies |
| 175 | Turner-Stokes et al. (Australia) | 2015 | **Comparison of Rehabilitation Outcomes for Long Term Neurological Conditions: A Cohort Analysis of the Australian Rehabilitation Outcomes Centre Dataset for Adults of Working Age** | no | 12527 | Unspecified (not SAH) | 1.0 | **Communication and social cognition** (FIM) | Trained clinical staff | non-parametric |
| 176 | Wang et al. (China) | 2015 | **Pulse Pressure and Cognitive Decline in Stroke Patients With White Matter Changes** | no | 406 | Ischemic and TIA | 16.0 | **Cognition** (MMSE and CDR) | NA | frequencies |
| 177 | Xia et al. (China) | 2015 | **Effect of Carotid Artery Stenting on Cognitive Function in Patients with Internal Carotid Artery Stenosis and Cerebral Lacunar Infarction: A 3-Year Follow-Up Study in China** | no | 579 | First ischemic stroke | 36.0 | **Cognitive function** (MMSE and MoCA) | Administered by physicians who had received systematic training. | mean & SD |
| 178 | Alexandrova et al. (Bulgaria) | 2016 | **Cognitive impairment one year after ischemic stroke: Predictors and dynamics of significant determinants** | no | 47 | Ischemic | 12.0 | **Cognitive status** (MMSE) | Not specified in methods | mean & SD |
| 179 | Alvarez-Sabin et al. (Spain) | 2016 | **Long-Term Treatment with Citicoline Prevents Cognitive Decline and Predicts a Better Quality of Life after a First Ischemic Stroke** | yes | 347 | First time ischemic | 24.0 | **Attention and EF** (Stroop, TMT A & B, SDMT, Mental Control [from WMS-III], DSB & DSF [from WMS-III]); **memory** (AVLT and VR [from WMS-III]); **language** (BNT, Verbal Fluency [animals] and COWAT, Pseudo words & Sentences Repetition, and Token Test); **spatial perception** (JLO); **motor speed** (GPT); **temporal orientation** (BTO) | Tests administered by two clinical neuropsychologists blinded to the patients' treatment. FU's evaluations completed by the same neuropsychologist in identical order of administration | incomplete |
| 180 | Aydin et al. (Turkey) | 2016 | **Functional independence measure scores of patients with hemiplegia followed up at home and in university hospitals** | yes | 144 | Ischemic and hemorrhagic | 28.9 | **Cognitive disability** (FIM) | Not specified in methods | mean & SD |
| 181 | Barker-Collo et al. (New Zealand) | 2016 | **Neuropsychological Outcome and its Predictors Across the First Year after Ischaemic Stroke** | no | 683 | Ischemic stroke | 12.0 | **Verbal memory, visual memory and working memory, psychomotor speed, reaction time, processing speed, executive functioning, attention and sustained attention, cognitive flexibility and social acuity** (CNS-vital signs test) | Computerised and self-administered | mean & SD |
| 182 | Benjamin et al. (England) | 2016 | **Progression of MRI markers in cerebral small vessel disease: Sample size considerations for clinical trials** | no | 120 | Lacunar infarct | 36.0 | **EF** (TMT B, WCST [modified version], and phonemic fluency [letters F-A-S]); **processing speed** (DSS [from WAIS-III], SIPT, and GPT) | NA | incomplete |
| 183 | Biffi et al. (USA) | 2016 | **Delayed seizures after intracerebral haemorrhage** | no | 978 | First ICH | 46.8 | **Cognitive performance** (TICS [modified version]) | Trained staff by telephone | no raw data |
| 184 | Blanchet et al. (Canada) | 2016 | **Cardiorespiratory fitness and cognitive functioning following short-term interventions in chronic stroke survivors with cognitive impairment: a pilot study** | yes | 14 | Chronic stroke (type not specified) | 54.5 | **Episodic memory** (HVLT-R); **working memory** (Brown-Peterson paradigm); **attention omission and commission errors** (CPT) | Assessors blinded to group allocation | mean & SD |
| 185 | Caratozzolo et al. (Italy) | 2016 | **Dementia after Three Months and One Year from Stroke: New Onset or Previous Cognitive Impairment?** | no | 114 | Ischemic or hemorrhagic | 12.0 | **Cognitive abilities** (MMSE [Italian telephone version]); **premorbid cognitive impairment** (IQCODE) | IQCODE was self- administered by a patient’s informant. MMSE administered by telephone | mean & SD |
| 186 | Chen et al. (China) | 2016 | **Intracranial Atherosclerosis and Poststroke Depression in Chinese Patients with Ischemic Stroke** | no | 207 | Ischemic stroke | 3.0 | **Global cognitive function** (MMSE [Chinese Version]) | Assessment performed by 2 qualified neurologists in the acute stage | mean & SD |
| 187 | Deniz et al. (Turkey) | 2016 | **Evaluation and follow-up of cognitive functions in patients with minor stroke and transient ischemic attack** | no | 40 | Minor stroke and TIA | 14.0 | **Cognitive functions** (IADL, MMSE); **attention** (forward & backward counting, and calculation test); **verbal memory** (word list memory [or flash memory], word list recall [or learning period], **and word list recognition** [or recall of knowledge]); **language** (BNT); **planning visuospatial abilities** (CDT); **constructing and visual memory** **[or visuospatial functions]** (construction ability); **EF** (FAB) | Not specified in methods | mean & SD |
| 188 | Di Cesare et al. (Multinational) | 2016 | **Phosphodiesterase-5 Inhibitor PF-03049423 Effect on Stroke Recovery: A Double-Blind, Placebo-Controlled Randomized Clinical Trial** | yes | 137 | acute ischemic stroke | 3.0 | [no area] (RBANS [Naming & Coding subtests]); [no area] (LCT); and **[no area]** (Recognition Memory Test [from RBANS]) | administered by examiners with appropriate clinical training and certification, provided by delegates of the sponsor prior to the study start | no raw data |
| 189 | Fang et al. (China) | 2016 | **Comprehensive rehabilitation with integrative medicine for subacute stroke: A multicenter randomized controlled trial** | yes | 156 | Ischemic stroke | 6.2 | **Cognitive impairment** (MMSE and MoCA) | Not specified in methods | mean & SD |
| 190 | Fordell et al. (Sweden) | 2016 | **RehAtt - scanning training for neglect enhanced by multi-sensory stimulation in Virtual Reality** | yes | 15 | Ischemic | 48.5 | **Spatial attention** (SCT, Baking Tray Task, LBT, Extinction, Posner Task [reaction time unified index]) | quiet dimmed room at a table with the computer screen straight ahead | non-parametric |
| 191 | Mutai et al. (Japan) | 2016 | **Longitudinal functional changes, depression, and health-related quality of life among stroke survivors living at home after inpatient rehabilitation** | no | 27 | Ischemic and hemorrhagic | 25.2 | **Cognitive disability** (FIM) | A certified occupational therapist conducted face-to-face interviews at the subjects’ homes. For subjects who had difficulty answering the survey questions, a family member responded instead. | mean & SD |
| 192 | Park et al. (South Korea) | 2016 | **The association between post-stroke depression and the activities of daily living/gait balance in patients with first-onset stroke patients** | no | 24 | First ever ischemic | 6.0 | **Cognitive function** (GDS) | Not specified in methods | mean & SD |
| 193 | Pavlovic et al. (Serbia) | 2016 | **Baseline characteristic of patients presenting with lacunar stroke and cerebral small vessel disease may predict future development of depression** | no | 487 | First lacunar stroke | 49.0 | **[no area]** (MMSE); **[no area]** (TMT A & B , WCST, ROCF, RAVLT, 60-item BNT, and Animal Naming test) | NA | frequencies |
| 194 | Rayegani et al. (Iran) | 2016 | **Evaluation of complete functional status of patients with stroke by Functional Independence Measure scale on admission, discharge, and six months poststroke** | no | 108 | first ever ischemic or hemorrhagic | 6.0 | **Communication and social cognition** (FIM) | evaluations completed by a physical medicine and rehabilitation specialist. In six-month follow- up visit, telephone calls were made and verbal answers were recorded | non-parametric |
| 195 | Rozental-Iluz et al. (Israel) | 2016 | **Improving executive function deficits by playing interactive video-games: secondary analysis of a randomized controlled trial for individuals with chronic stroke** | yes | 39 | Ischemic and hemorrhagic | 47.4 | **EF -** visuomotor scanning, divided attention and cognitive flexibility **(TMT A & B); EF - initiation, planning, judgment and completion** (EFPT [bill payment subtest]); **EF - information seeking, problem solving, planning and working memory** (EFRT) | Assessments were administered pre and post the intervention and at 3-month FU by assessors blind to treatment allocation. | mean & SD |
| 196 | Turunen et al. (Finland) | 2016 | **Executive Impairment Is Associated with Impaired Memory Performance in Working-Aged Stroke Patients** | no | 223 | First infarction [ischemic] | 24.0 | **List learning** (learning of 10 unrelated words [IR & DR]); **IR and DR** (LM I & LM II); **visual memory** (BRVRT)**; reasoning** (similarities, information, digit symbol, and BDT); **EF** (TMT [set B minus A], Stroop [modified] [set B minus A], DSB [from WAIS - III]) | A clinical neuropsychologist performed the neuropsychological examinations according to a written research protocol | mean & SD |
| 197 | Uiterwijk et al. (Netherlands) | 2016 | **Total cerebral small vessel disease MRI score is associated with cognitive decline in executive function in patients with hypertension** | no | 62 | First lacunar | 48.0 | **Memory** (IR, DR, and DRec [from RAVLT], and DSF [from WAIS-III]); **EF** (Stroop [interference score], TMT [interference score], category fluency [animals & professions], letter fluency, letter-number fluency, letter-number sequencing [from WAIS-III], and DSB [from WAIS-III]); **information processing speed** (symbol substitution - coding subtest [from WAIS-III], TMT A, and Stroop [SCWT parts 1 & 2]) | NA | no raw data |
| 198 | Wang et al. (China) | 2016 | **Risk Factors and Cognitive Relevance of Cortical Cerebral Microinfarcts in Patients With Ischemic Stroke or Transient Ischemic Attack** | no | 231 | Ischemic and TIA | 28.0 | **Global cognitive function** (MoCA) | NA | mean & SD |
| 199 | Wang et al. (China) | 2016 | **Efficacy and safety assessment of acupuncture and nimodipine to treat mild cognitive impairment after cerebral infarction: a randomized controlled trial** | yes | 119 | Ischemic | 9.0 | **Cognitive function** (MoCA) | Both the trained assessor, who evaluated patient MoCA score, and the statistician, who conducted the data analysis, were blinded to the treatment allocation. | mean & SD |
| 200 | Arba et al. (Multinational) | 2017 | **Determinants of post-stroke cognitive impairment: analysis from VISTA** | no | 5453 | First ischemic, hemorraghic, and TIA | 36.0 | **Cognitive impairment** (MMSE) | Not addressed | no raw data |
| 201 | Askin et al. (Turkey) | 2017 | **Effects of low-frequency repetitive transcranial magnetic stimulation on upper extremity motor recovery and functional outcomes in chronic stroke patients: A randomized controlled trial** | yes | 40 | First ever ischemic | 26.5 | **Cognition (MMSE and FIM)** | Blinded asssessor familiar with the assessments | non-parametric |
| 202 | Bath et al. (England) | 2017 | **Intensive versus Guideline Blood Pressure and Lipid Lowering in Patients with Previous Stroke: Main Results from the Pilot 'Prevention of Decline in Cognition after Stroke Trial' (PODCAST) Randomised Controlled Trial** | yes | 83 | Ischemic and hemorrhagic | 28.5 | **Cognition** (MMSE, Stroop, TMT A & B, category fluency [animal naming], and TICS [modified version]); **executive and attentional tasks** (ACE-R); **premorbid cognitive function** (IQCODE); **dementia** (knafelc [MMSE + IQCODE]) | Clinic measurements performed and recorded by trained research nurses/coordinators. Telephone follow-up was performed centrally, again by trained staff. | mean & SD |
| 203 | Bunketorp-Kall et al. (Sweden) | 2017 | **Long-Term Improvements After Multimodal Rehabilitation in Late Phase After Stroke: A Randomized Controlled Trial** | yes | 123 | Ischemic and hemorrhagic | 42.1 | **Higher cerebral functions** (BNIS); **working memory** (letter-number sequencing) | Observer assessed | non-parametric |
| 204 | Davalos et al. (Spain) | 2017 | **Safety and efficacy of thrombectomy in acute ischaemic stroke (REVASCAT): 1-year follow-up of a randomised open-label trial** | yes | 207 | Ischemic | 12.0 | **Cognitive status** (TMT) | Not addressed | no raw data |
| 205 | De Boer et al. (Netherlands) | 2017 | **The Role of the BDNF Val66Met Polymorphism in Recovery of Aphasia After Stroke** | no | 60 | Ischemic and hemorrhagic | 2.8 | Communication in daily life situations (ANELT); word finding (BNT) | Not addressed | mean & SD |
| 206 | Dignam et al. (Australia) | 2017 | **Influence of Cognitive Ability on Therapy Outcomes for Anomia in Adults With Chronic Poststroke Aphasia** | yes | 34 | Stroke patients (type undefined) | 51.0 | **Language** (CAT); **attention** (TEA); **verbal memory and learning** (HVLT-R); **visuospatial memory and learning** (BVMT-R); **EF** (TMT B and sorting subtest [both from D-KEFS]) | NA | no raw data |
| 207 | Geng et al. (China) | 2017 | **Midterm blood pressure variability is associated with poststroke cognitive impairment: A Prospective cohort study** | no | 708 | Ischemic | 12.0 | Cognitive function (MoCA) | Trained neurologists | mean & SD |
| 208 | Glize et al. (France) | 2017 | **Language features in the acute phase of poststroke severe aphasia could predict the outcome** | no | 86 | First ischemic or hemorrhagic | 3.0 | **Language** (LAST and ASRS) | Not addressed | frequencies |
| 209 | Guekht et al. (Multinational) | 2017 | **ARTEMIDA Trial (A Randomized Trial of Efficacy, 12 Months International Double-Blind Actovegin): A Randomized Controlled Trial to Assess the Efficacy of Actovegin in Poststroke Cognitive Impairment** | yes | 503 | Ischemic | 12.0 | **Cognition** (MoCA and ADAS-cog) | Not addressed | non-parametric |
| 210 | Hung et al. (Taiwan) | 2017 | **Cognitive effects of weight-shifting controlled exergames in patients with chronic stroke: a pilot randomized comparison trial** | yes | 37 | Ischemic and hemorrhagic | 30.0 | **Cognitive ability** (CASI [chinese version]) | Research assisstant blinded to group assignment | non-parametric |
| 211 | Lee et al. (Taiwan) | 2017 | **The Effect of a Virtual Reality Game Intervention on Balance for Patients with Stroke: A Randomized Controlled Trial** | yes | 50 | Ischemic and hemorrhagic | 32.5 | **[no area]** (TUG-Cog) | Blinded therapist | mean & SD |
| 212 | Lim et al. (Korea) | 2017 | **A Methodological Perspective on the Longitudinal Cognitive Change after Stroke** | no | 375 | Ischemic | 34.1 | **Cognition** (K-VCIHS) | Not addressed | no raw data |
| 213 | Nijsse et al. (Netherlands) | 2017 | **Temporal Evolution of Poststroke Cognitive Impairment Using the Montreal Cognitive Assessment** | no | 324 | Ischemic and hemorrhagic | 6.0 | **Cognitive functioning** (MoCA) | Assessments of cognitive functioning were conducted by a trained research assistant | mean & SD |
| 214 | Ojagbemi et al. (Nigeria) | 2017 | **Stroke severity predicts poststroke delirium and its association with dementia: Longitudinal observation from a low income setting** | no | 101 | Ischemic or hemorrhagic | 3.0 | **Global cognitive impairment** (MMSE); **memory registration** (10 Word list learning); **recall** (delayed recall test); EF (animal naming test) | NA | no raw data |
| 215 | Penn et al. (South Africa) | 2017 | **Early recovery profiles of language and executive functions after left hemisphere stroke in bilingualism** | no | 19 | Ischemic and hemorrhagic | 4.0 | **Language** (CAT); **EF** (n-back, stroop [victoria version], WCST, number-letter [shifting]) | Home or patient's work in a quiet room | mean & SD |
| 216 | Poulin et al. (Canada) | 2017 | **Comparison of two cognitive interventions for adults experiencing executive dysfunction post-stroke: a pilot study** | yes | 9 | Ischemic and hemorrhagic | 8.0 | **EF - speed, attention and cognitive flexibility** (TMT); **EF - cognitive flexibility and inhibition** (Stroop-CWIT [from the D-KEFS]); **working memory** (DS [from WAIS-IV]) | Outcome measures were administered at baseline, post-intervention and one-month follow-up by a blinded evaluator | mean & SD |
| 217 | Prince et al. (Canada) | 2017 | **Persistent visual perceptual disorders after stroke: Associated factors** | no | 70 | Ischemic and hemorrhagic | 9.7 | **Memory** (iR & DR [from WMS]); **inhibition** (stroop); **verbal comprehension** (token test); word reading (Montreal-Tolouse) | Not addressed | no raw data |
| 218 | Prior et al. (Canada) | 2017 | **Comprehensive Cardiac Rehabilitation for Secondary Prevention After Transient Ischemic Attack or Mild Stroke PSYCHOLOGICAL PROFILE AND OUTCOMES** | no | 100 | Ischemic and TIA | 10.5 | **Cognitive impairment** (MMSE); **attention and working memory** (DS and TMT A); **learning** (RAVLT [trials 1-5]); **memory post-interference** (RAVLT [trial 6]); **memory** (RAVLT [trial 7 - 20-min delay]); **speed, EF, and abstraction** (Digit-Symbol, TMT B, clock-drawing, FAS oral-verbal fluency, and Similarities); **pre-morbid intellectual functioning** (NAART) | Neurocognitive testing was administered by experienced psychometrists | mean & SD |
| 219 | Sagnier et al. (France) | 2017 | **Admission Brain Cortical Volume: An Independent Determinant of Poststroke Cognitive Vulnerability** | no | 248 | Ischemic | 12.0 | **Global cognition** (MoCA); **EF** (Isaacs test); **[no area]** (Zazzo's cancellation task) | Administered in a dedicated room by a neurologist together with a clinical research assisstant | mean & SD |
| 220 | Schleiger et al. (Australia) | 2017 | **Poststroke QEEG informs early prognostication of cognitive impairment** | no | 35 | Ischemic | 3.3 | **Mild cognitive impairment** (MoCA) | Trained assessor | mean & SD |
| 221 | Scrutinio et al. (Italy) | 2017 | **Development and Validation of a Predictive Model for Functional Outcome After Stroke Rehabilitation: The Maugeri Model** | no | 875 | Ischemic and hemorrhagic | 2.6 | **Cognition** (FIM) | Trained therapists administered assessments | non-parametric |
| 222 | Shi et al. (China) | 2017 | **Effect and safety of arterial thrombolysis added to solitaire AB stents interventional treatment in acute ischemic stroke** | yes | 72 | Ischemic | 0.7 | **Cognition** (Glasgow outcome scale) | Not addressed | mean & SD |
| 223 | Sivakumar et al. (Canada) | 2017 | **White matter hyperintensity volume predicts persistent cognitive impairment in transient ischemic attack and minor stroke** | no | 115 | Ischemic | 3.0 | **Cognition** (MoCA) | Not addressed | mean & SD |
| 224 | Tan et al. (Singapore) | 2017 | **Decline in changing Montreal Cognitive Assessment (MoCA) scores is associated with post-stroke cognitive decline determined by a formal neuropsychological evaluation** | no | 275 | Ischemic and TIA | 12.0 | **Cognitive screening** (MMSE); **mild cognitive impairment** (MoCA) | Administered by trained research psychologists | non-parametric |
| 225 | Tessier et al. (France) | 2017 | **Resting Heart Rate Predicts Depression and Cognition Early after Ischemic Stroke: A Pilot Study** | no | 54 | First ischemic | 3.0 | **Global cognitive function** (MoCA) | Not addressed | mean & SD |
| 226 | Tse et al. (Australia) | 2017 | **Increased work and social engagement is associated with increased stroke specific quality of life in stroke survivors at 3 months and 12 months post-stroke: A longitudinal study of an Australian stroke cohort** | no | 185 | Ischemic | 12.0 | **Cognitive impairment** (MoCA) | Health professionals trained in the conduct of outcome measures | non-parametric |
| 227 | Yan et al. (China) | 2017 | **Dl-3-n-butylphthalide can improve the cognitive function of patients with acute ischemic stroke: a prospective intervention study** | yes | 104 | First ischemic | 1.0 | **Cognitive function** (MoCA and MMSE) | The enrolled patients were independently assessed by a trained neurology physician using standard instructions | no raw data |
| 228 | Yoon et al. (Korea) | 2017 | **Factors associated with improvement or decline in cognitive function after an ischemic stroke in Korea: the Korean stroke cohort for functioning and rehabilitation (KOSCO) study** | no | 6625 | First time ischemic | 12.0 | **Cognitive function** (MMSE [Korean Version]) | Not specified in methods | mean & SD |
| 229 | Zhang et al. (China) | 2017 | **Leukoaraiosis is Associated with Worse Short-Term Functional and Cognitive Recovery after Minor Stroke** | no | 217 | First ischemic | 1.0 | **Cognition** (MMSE) | Evaluated by srtoke trained physician | no raw data |
| 230 | Zhao et al. (China) | 2017 | **Comparing Cerebralcare Granule and aspirin for neurological dysfunction in acute stroke in real-life practice** | yes | 3251 | Stroke (undefined) | 3.2 | **Neurological function** (MMSE and MoCA) | Evaluators trained in scoring before the study and were blinded to treatment allocation. | mean & SD |
| 231 | Zhou et al. (China) | 2017 | **The efficacy of gastrodin in combination with folate and vitamin B12 on patients with epilepsy after stroke and its effect on HMGB-1, IL-2 and IL-6 serum levels** | yes | 92 | Stroke (not specified) | 94.2 | **Cognition** (MoCA) | Not addressed | mean & SD |
| 232 | Bush et al. (USA) | 2018 | **Fever Burden and Health-Related Quality of Life After Intracerebral Hemorrhage** | no | 106 | ICH | 12.0 | **EF** (Neuro-QoL) | Internet survey or telephone interview | mean & SD |
| 233 | De Luca et al. (Italy) | 2018 | **Computerized Training in Poststroke Aphasia: What About the Long-Term Effects? A Randomized Clinical Trial** | yes | 32 | First ever ischemic | 14.8 | **Language comprehension** (Token Test); **language impairment** (NPEA); **attention, visual search, scanning, sequencing, shifting, psychomotor speed, abstraction, flexibility, and EF** (TMT); **praxis** (constructional apraxia test, and ideomotor apraxia test); **visual selective attention** (attentive matrices test) | Neuropsychologist blinded to treatment allocation | no raw data |
| 234 | Dickie et al. (Scotland) | 2018 | **The brain health index: Towards a combined measure of neurovascular and neurodegenerative structural brain injury** | no | 157 | Ischemic | 12.0 | **Cognitive impairment** (ACER) | Not addressed | no raw data |
| 235 | Douven et al. (Netherlands) | 2018 | **Co-occurrence of depressive symptoms and executive dysfunction after stroke: associations with brain pathology and prognosis** | no | 245 | Ischemic and hemorrhagic | 15.0 | **Global cognition** (MMSE); **EF** (TMT B, 1-min fluency test [animals & professions], zoo map [from BADS], and key search [from BADS]); **episodic verbal memory** (RAVLT); **information processing** (TMT A and DSS) | Not addressed | no raw data |
| 236 | Ezeugwu et al. (Canada) | 2018 | **The Feasibility and Longitudinal Effects of a Home-Based Sedentary Behavior Change Intervention After Stroke** | yes | 34 | Ischemic and hemorrhagic | 7.5 | **Cognitive status** (MoCA) | Not addressed | mean & SD |
| 237 | Faria et al. (Portugal) | 2018 | **Combined Cognitive-Motor Rehabilitation in Virtual Reality Improves Motor Outcomes in Chronic Stroke - A Pilot Study** | yes | 32 | Ischemic and hemorrhagic | 35.0 | **Cognition** (MoCA); **attention** (single LCT, digit cancellation, and Bells Test) | Assessor not blinded | non-parametric |
| 238 | Fonseca et al. (Portugal) | 2018 | **Cognitive performance and aphasia recovery** | no | 50 | First ischemic | 3.3 | **Memory** (5 objects memory test, spatial span [from WMS-III], memory of faces [from WMS-III], camel & cactus test); **EF** (Tower of Hanoi, Matrix reasoning [from WASI], clock drawing [from BLAD], and motor initiative [from BLAD]); **attention and speed processing** (symbol search [from WAIS], cancelation task [from BLAD]); **language** (BAAL, speech fluency, object naming, verbal comprehension [object identification & sentence comprehension], word repetition, and Token test [22-item-sf]) | Not addressed | mean & SD |
| 239 | Furie et al. (Multinational) | 2018 | **Effects of pioglitazone on cognitive function in patients with a recent ischaemic stroke or TIA: A report from the IRIS trial** | yes | 3876 | Ischemic or TIA | 60.0 | **Cognitive function** (MMSE [modified version]) | In-person visits | frequencies |
| 240 | Grau-Sanchez et al. (Spain) | 2018 | **Music-supported therapy in the rehabilitation of subacute stroke patients: a randomized controlled trial** | yes | 40 | First ischemic or hemorrhagic | 3.2 | **Working memory and attention** (DSF and DSB); **response inhibition** (Stroop); **processing speed and mental flexibility** (TMT); **verbal memory** (RAVLT and RBMT) | Performed by a neuropsychologist | mean & SD |
| 241 | Kim et al. (Korea) | 2018 | **Can the integrity of the corticospinal tract predict the long-term motor outcome in poststroke hemiplegic patients?** | no | 48 | first ischemic and hemorrhagic | 24.0 | **Cognitive function** (MMSE) | Not addressed | mean & SD |
| 242 | Lee et al. (Korea) | 2018 | **Effect of Computerized Neuropsychologic Test in Subacute Post-Stroke Patient With Cognitive Impairment** | no | 125 | Ischemic and hemorrhagic | 5.3 | **Cognitive function** (MMSE [Korean Version]) | Not addressed | mean & SD |
| 243 | Makin et al. (Scotland) | 2018 | **The effect of different combinations of vascular, dependency and cognitive endpoints on the sample size required to detect a treatment effect in trials of treatments to improve outcome after lacunar and non-lacunar ischaemic stroke** | no | 264 | 154/110 | 12.0 | **Cognitive function** (ACER) | Evaluations in clinical research center, by telephone or via their GP | no raw data |
| 244 | Nijboer et al. (Netherlands) | 2018 | **Impact of clinical severity of stroke on the severity and recovery of visuospatial neglect** | no | 90 | First ischemic | 6.5 | **Visuospatial neglect** (LCT) | Not addressed | non-parametric |
| 245 | Nyberg et al. (Norway) | 2018 | **A longitudinal study of computerized cognitive training in stroke patients - effects on cognitive function and white matter** | yes | 26 | first ischemic, hemorrhagic and SAH | 44.9 | **General cognition** (vocabulary [from WASI], matrix reasoning [from WAIS]); **visuospatial working memory** (grid & cube); **verbal working memory and visuospatial working memory** (numbers); **verbal working memory** (hidden numbers); **working memory** (letter memory, DSF, DSB, CVLT-II, RCFT [recall score], n-back paradigm [2-back & 3-back condition], spatial working memory test [from CANTAB]); **EF** (plus-minus task [shifting], and stroop) | Not addressed | mean & SD |
| 246 | Oh et al. (Korea) | 2018 | **A 2‐year prospective follow‐up study of temporal changes associated with post‐stroke cognitive impairment** | no | 52 | Ischemic and hemorrhagic | 24.0 | **Cognitive impairment** (MVCI) | Face-to-face or telephone interviews | mean & SD |
| 247 | Pan et al. (China) | 2018 | **Effects of paroxetine on motor and cognitive function recovery in patients with non-depressed ischemic stroke: An open randomized controlled study** | yes | 170 | Ischemic | 6.0 | **Cognitive impairment** (MoCA) | Blinded, trained evaluators | mean & SD |
| 248 | Pang et al. (China) | 2018 | **Dual-Task Exercise Reduces Cognitive-Motor Interference in Walking and Falls After Stroke A Randomized Controlled Study** | yes | 84 | Ischemic and hemorrhagic | 79.3 | **Verbal fluency** (naming [categories]); **mental tracking** (serial-3-subtractions) | Blinded researchers | no raw data |
| 249 | Pasinska et al. (Poland) | 2018 | **Frequency and predictors of post-stroke delirium in PRospective Observational POLIsh Study (PROPOLIS)** | no | 750 | Ischemic, hemorrhagic and TIA | 0.2 | **Cognition** (MoCA) | Assessed in hospital | no raw data |
| 250 | Salihovic et al. (Bosnia & Herzegovina) | 2018 | **Cognitive syndromes after the first stroke** | no | 275 | First ischemic or hemorrhagic | 12.0 | **Cognitive function** (MMSE, MoCA, WAIS, WMS, ROCFT, and DRS) | Not addressed | no raw data |
| 251 | Turunen et al. (Finland) | 2018 | **Domain-specific cognitive recovery after first-ever stroke: A 2-year follow-up** | no | 153 | First ischemic | 24.0 | **EF** (TMT A & B, DSB [from WAIS-III], and phonemic fluency); **verbal memory** (list learning, LM-I & LM-II [from WMS-R]); **visual memory** (BRVRT); **psychomotor speed** (TMT A, tapping-device on each hand); **visuospatial functions** (visuospatial searching task, and time to draw 4 figures); **language** (Token Test [SF], modified visual naming task [from BDAE], and repetition of a long sentence); **and reasoning** (similarities, and BDT [from WAIS-III]) | Neuropsychologist evaluated patients according to a written protocol | non-parametric |
| 252 | Vaes et al. (Belgium) | 2018 | **Rehabilitation of visuospatial neglect by prism adaptation: effects of a mild treatment regime. A randomised controlled trial** | yes | 43 | Stroke (not specified) | 4.7 | **Neglect** (Visuospatial Neglect Test Battery of Vaes [cancellation + bisection & drawing + peripersonal navigation + visual extinction + anterograde memory]) | Not addressed | non-parametric |
| 253 | Zengin-Metli et al. (Turkey) | 2018 | **Effects of robot assistive upper extremity rehabilitation on motor and cognitive recovery, the quality of life, and activities of daily living in stroke patients** | yes | 35 | Stroke (WHO definition) | 3.4 | [no area] (MMSE and FIM) | Not addressed | mean & SD |
| 254 | Zietemann et al. (Multinational) | 2018 | **Early MoCA predicts long-term cognitive and functional outcome and mortality after stroke** | no | 274 | Ischemic and hemorrhagic | 36.0 | **Global cognition** (MoCA); **EF and attention** (TMT B, TMT A, Stroop, DSST); **memory** (Word List Memory [encoding], recall, discriminability [recognition], constructional praxis [recall & savings], FCSRT [total of the 3 free recalls trials], FCSRT [delayed free recall], RCFT [immediate & delayed recall]); **language** (DO-80, BNT, semantic fluency [animals], phonemic fluency [s-words]); **visuospatial ability** (CERAD [copy visual construction], RCFC, and VOSP) | Face-to-face interviews | frequencies |
| 255 | Griauzde et al. (USA) | 2019 | **A Population-Based Study of Intracerebral Hemorrhage Survivors' Outcomes** | no | 245 | ICH | 3.0 | **Cognitive function** (MMSE [modified]) | Not addressed | non-parametric |
| 256 | Huang et al. (China) | 2019 | **Predictors of Remission of Early-Onset Poststroke Depression and the Interaction Between Depression and Cognition During Follow-Up** | no | 820 | Ischemic and hemorrhagic | 3.0 | **Cognitive impaiment** (MMSE) | Blinded neurologists | mean & SD |
| 257 | Sun et al. (Canada) | 2019 | **Lithium Carbonate in a Poststroke Population Exploratory Analyses of Neuroanatomical and Cognitive Outcomes** | yes | 12 | Ischemic | 5.0 | **Cognitive function** (MMSE and MoCA); **verbal memory** (HVLT-R) | Not addressed | non-parametric |

List of Abbreviations

10 WDRT= 10 words list learning and delayed recall tests

3MSE= Modified Mini Mental State Examination

ACE-R= Addenbrooke’s Cognitive Examination-R

ADAS= Alzheimer's Disease Assessment Scale

ADL= Activities of Daily Living Scale

ADT= Auditory Detection Test

ALL= Auditory List Learning

AM-PAC= Activity Measure for Post-Acute Care - Computer Adaptive test version

AMT= Abbreviated Mental Test

ANELT= Amsterdam Nijmegen Everyday Language Test

ASRS= Aphasia Severity Rating Scale

ATB= Attention Test Battery

AVLT= Auditory Verbal Learning test

BAAL= Lisbon Battery for Assessment of Aphasia

BAB= Boston Aphasia Battery

BADS= Behavioral Assessment of Dysexecutive Syndrome

BCT= Bells Cancellation Test

BDAE= Boston Diagnostic Aphasia Examination

BDI= Beck Depression Inventory

BDT= Block Design Test

BEM 144= Batterie d’évaluation de l’efficience mnésique

BIT= Behavior Inattention Test

BLAD= Lisbon Battery for Assessment of Dementia

BLO= Benton Line Orientation

BMIPB=BIRT Memory and Information Processing Battery

BNIS= Barrow Neurological Institute Screen

BNT= Boston Naming Test

BRVRT= Benton Revised Visual Retention Test

BSAT= Brixton Spatial Anticipation Test

BTO= Benton's Temporal Orientation

BUN= Neglect for personal body space

BVMT-R= Brief Visuo-spatial Memory Test – Revised

BVRT= Benton Visual Retention Test

CAMCOG= Cambridge Cognition Examination

CAMT= Camden Memory Tests

CAS= Cognitive Ability Scale

CASI= Cognitive Abilities Screening Instrument

CAT= Comprehensive Aphasia Test

CBS= Corsi Block Span

CBT= Corsi Blocks Test

CCPT= Connor's Continuous Performance Test

CDR= Clinical Dementia Scale

CDRCP= Cognitive Drug Research Computarised Battery

CDT= Clock Drawing Test

CERAD= Consortium to Establish a Registry for Alzheimer's Disease

CFST= Color Form Sorting Test

CIMP-QUEST= Cognitive Impairment Questionnaire

CMT= Cronholm‒Molander Test

CNS= Canadian Neurological State

CNST= Conversational Narrative Speech Test

CNT= Computerized Neuropsychological Test

COGNISTAT= Neurobehavioral Cognitive Status Examination

COWAT= Controlled Oral Word Association Test

CP= Clock Perception

CPS= Cognitive Performance Scale

CPT= Continuous Performance Test

CRT= Chapman Reading Task

CST= Concept Shifting Test

CTPMA= Chicago Tests of Primary Mental Abilities

CVLT= California Verbal Learning Test

D2= Aufmerksamkeits-Belastungs-Test

DAS= Dutch Aphasia Society

DASS= Depression Anxiety Stress Scale

DEX= Dysexecutive Questionnaire

DMN= Default Mode Network

DO-80= test de Dénomination Orale d’images-80

DR= Delayed Recall

DRec= Delayed Recognition

DRS= Dementia Rating Scale

DS=Digit Span

DSB= Digit Span Backward

DSCT= Digit Symbol-Coding Test

DSF= Digit Span Forward

DSM IV= Diagnostic and Statistic Manual of Mental Disorders, Fourth Edition

DSMT= Digit Symbol Modalities Test

DSS= Digit Symbol Substitution

DST= Digit Symbol Test

ECAQ= Elderly Cognitive Assessment Questionnaire

EDI= Executive Dysfunction Index

EF= Executive Functioning

EFPT= Executive Function Performance Test

EFRT= Executive Function Route Finding Task

EXIT= Executive Interview

FAB= Frontal Assessment Battery

FCSRT= Free and Cued Selective Reminding Test

FOM= Fuld Object-Memory Evaluation

FU= Follow-up

FUN= neglect for far space

GCT= Greek Cross Test

GDS= Global Deterioration Scale

GIS= Groninger Intelligence Scale

GIT= Groninger Intelligence Scale

GNT= Graded Naming Test

GPT= Grooved Pegboard Test

HIS= Hachinski Ischemic Score

HRQoL= Health-Related Quality of Life

HVLT-R= Hopkins Verbal Learning Test- Revised Version

HVLT= Hopkins Verbal Learning Test

I-Flex= Short form of the Executive Interview

IADL= Instrumental Activities of Daily Living

IPS= Information Processing Speed

IR= Immediate Recall

IR= Immediate Recall

IST= Isaacs Set Test

IVA-CPT= Integrated Visual Auditory Continuous Performance Test

JLO= Judgment of Line Orientation

K-SNAP= Kaufman short neuropsychological assessment procedure

K-VCIHS= Korean version of the Vascular Cognitive Impairment Harmonization Standards

KSNAP= Kaufman short neuropsychological assessment procedure

LAST= Language Screening Test

LBT= Line Bisection Test

LCF= Levels of Cognitive Functioning

LCT= Letter Cancellation test

LLT= Location Learning Test

LM= Logical Memory

LOS= Length of stay

LOTCA= Lowenstein Occupational Therapy of Cognitive Assessment

MBEA= Montreal Battery of Evaluation of Amusia

mBN= Modified Boston Naming

MCST= Modified Card Sorting Test

MDRS= Mattis Dementia Rating Scale

MDRS= Mattis Dementia Rating Scale

MIS= Memory Impairment Screen

mLLT= Modified Location Learning Test

MMSE [std] = Standardized Mini-Mental State Examination

MMSE= Mini Mental State Examination

MMT= Modified Mental Test

MoCA= Montreal Cognitive Assessment

mRS= Modified Rankin Scale

MSOT= Mental Slowness Observation Test

MSQ= Mental Slowness Questionnaire

MTT= Modified Token Test

MVCI= mild cognitive impairment assessment

MVPT-v= motor free visual perceptual test, vertical version

mWCST= Modified Wisconsin Card Sort Testing

n.s.= not significant

NART-R= National Adult Reading Test-Revised

NCSE= Neurobehavioral Cognitive Status Examination

NPEA= neuropsychological exam for aphasia

NPI= Neuropsychiatric Inventory

NTCCTB= NeuroTrax computerized cognitive test battery

ONT= Oldfield Naming Test

OT= Occupational Therapy

P-S= Post-stroke

PASAT= Paced Auditory Serial Addition Test

PR= Picture Recall

PSD= Post-Stroke Depression

PT= Physical Therapy

PUN= Neglect for peripersonal state

R-CAMCOG= Rotterdam CAMCOG

RAPM= Raven Advanced Progressive Matrices

RAVLT= Rey Auditory Verbal Learning Test

RAVMT= Rey Auditory Verbal Memory Task

RBANS= Repeatable Battery for the Assessment of Neuropsychological Status

RBMT= The Rivermead Behavioural Memory Test

RCFT= Rey-Osterrieth Complex Figure Test

RCM= Raven's coloured matrices

RCPM= Raven's Coloured Progressive Matrices

RIC-FAS= Rehabilitation Institute of Chicago Functional Assessment Scale

RMT= Recognition Memory Test

RO= Rey Osterrieth

ROCF= Rey-Osterrieth Complex Figure Test

RSCM= Raven's Standard Coloured Matrices

RSPM= Raven's Standard Progressive Matrices

RVR= Rapid Verbal Retrieval

RWT= Regensburger Wortflüssigkeits-Test

SBT= Short Blessed Test

SCNT= Seoul-computerized neuropsychological tests

SCT= Star Cancellation Test

SDMT= Symbol Digit Modalities Test

SDSA= Stroke Driver Screening Assessment

SEVLT= Spanish and English Verbal Learning Test

SF= Short From

SIPT= Speed of Information Processing Task

SOIP= Speed of information Processing Motor control Task

SPMSQ= Short Portable Mental Status Questionnaire

SRMT= Short recognition Memory Test

SRT= Simple Reaction Time

ST= Speech Therapy

STT= Short Token Test

TAP= Testbatterie zur Aufmerksamkeitsprüfung

TAPr= Test for Attention Performance

TBAP= Test Battery for attention Performance

TEA= Test of Everyday Attention

TFP= Test of Facial Perception

TFR= Test of Facial Recognition

TICS= Telephone Interview for Cognitive Status

TMT= Trail Making Test

ToL= Tower of London

UFOV= Useful test of view

VET= Visual Elevator Test

VFT= Phonemic verbal fluency test

VMSB= Visual Memory Span Backward

VMSF= Visual Memory Span Forward

VNT= Visual Naming Test

VOSPB= Visual Object and Space Perception Battery

VPA= Visual Paired Associates

VR= Visual Reproduction

VS= Visual Span

VSAT= Visual Semantic Association Test

VSB= Visual Span Backward

VSF= Visual Span Forward

WAB= Western Aphasia Battery

WAIS= Wechsler Adult Intelligence Scale

WASI= Wechsler Abbreviated Scale for Intelligence

WCFST= Weigl Colour Form Sorting Task

WCST= Wisconsin Card Sort Test

WDS= Wechsler Digit Span

WLL= Word Learning List

WLM= Wechsler Logical Memory

WLR= Word List Recall

WMS= Wechsler Memory Scale

WTAR= Wechsler Test of Adult Reading

WWT= walking while taking
