# Supplementary material for: Longitudinal evaluation of cognition after stroke – A systematic scoping review
Source: PLoS One. 2019 Aug 29;14(8):e0221735. doi: 10.1371/journal.pone.0221735 (PMC6715188; doi:10.1371/journal.pone.0221735)
Supplement: S1 Fig — (PDF) [file pone.0221735.s005.pdf]

Scoping review – S1 Figure

Recoding of cognitive domains and instruments

Domain cleaning according to the ICF (code extract)

```
  x[x=='Cognition'|
      x=='Cognitive'|
      x=='Cognitive Abilities'|
      x=='Cognitive Ability'|
      x=='Cognitive Decline'|
      x=='Cognitive Disability'|
      x=='Cognitive Disorders'|
      x=='Cognitive Dysfunction'|
      x=='Cognitive Function'|
      x=='Cognitive Functioning'|
      x=='Cognitive Functions'|
      x=='Cognitive Impact Resulting from Stroke'|
      x=='Cognitive Impairment'|
      x=='Cognitive Mental Status'|
      x=='Cognitive Performance'|
      x=='Cognitive State'|
      x=='Cognitive Status'|
      x=='Degree of Cognitive Abilities'|
      x=='General Cognition'|
      x=='General Cognitive Function'|
      x=='General Cognitive Functioning'|
      x=='General Cognitive Functions'|
      x=='Global Cognition'|
      x=='Global Cognitive Assessment'|
      x=='Global Cognitive Function'|
      x=='Global Cognitive Functioning'|
      x=='Global Cognitive Functions'|
      x=='Global Cognitive Impairment'|
      x=='Global Cognitive Performance'|
      x=='Global Cognitive Status'|
      x=='Mental Status'|
      x=='Mild Cognitive Impairment'|
      x=='Moderate Cognitive Dysfunction'|
      x=='Neuropsychological Functions'|
      x=='Neuropsychological Impairment'|
      x=='Premorbid Cognitive Function'|
      x=='Premorbid Cognitive Impairment'|
      x=='Recovery of Cognition'|
      x=='Cognitive Changes-State of Higher Mental Functions'|
      x=='General Cognition-Higher Cerebral Functions'|
      x=="Cognitive Impairment"|
      x=="Cognitive Screening"
      ]="Global (ICF-ch1)"
```

Raw Domains

"ICF" Domain

```
x[x=='Alertness'|
        x=='Intrinsic Alertness'|
        x=='Vigilance'
    ]='Consciousness (b110)'

x[x=='Orientation'|
        x=='Orientation to Time & Space'|
        x=='Temporal Orientation'
    ]='Orientation (b114)'

x[x=='Constructing'|
        x=='Construction'|
        x=='Construction Skills'|
        x=='Constructional'|
        x=='Constructional Function'|
        x=='Constructional Praxis'|
        x=='Constructive Praxis'|
        x=='Visuospatial Construction'|
        x=='Visuoconstruction'|
        x=='Visuoconstructive Ability'|
        x=='Visuoconstuctive Functions'
    ]="HLCF (b164)"

x[x=='Neurologic Function'|
        x=='Neurological Function'
    ]="Neurologic Function (ICF-ch7)"

x[x=='Dementia'|
    x=='Dementia Diagnosis'|
    x=='Dementia Diagnostic Instrument'|
    x=='General Inteligence'|
    x=='General Intellectual Functioning'|
    x=='General Intelligence'|
    x=='Intellectual Abilities'|
    x=='Intellectual Ability'|
    x=='Pre-Morbid Intellectual Functioning'
    ]= 'Intellectual (b117)'

    x[x=='Social Acuity'|
        x=='Social Cognition'|
        x=='Social Interaction'
    ]='Global Psychosocial (b122)'

    x[x=='Alternating Attention'|
        x=='Attention'|
        x=='EF - Attention'|
        x=='Attention-Phasic Alert'|
        x=='Attentional Tasks'|
        x=='Attentiveness'|
```

```
        x=='Concentration'|
        x=='Divided'|
        x=='Divided Attention'|
        x=='EF - Divided Attention'|
        x=='EF - Attention Switching'|
        x=='Focused Visual Attention'|
        x=='General Visual Attention'|
        x=='Phasic Attention'|
        x=='Selective'|
        x=='Selective Attention'|
        x=='Spatial Attention'|
        x=='Sustained'|
        x=='Sustained Attention'|
        x=='Switching of Attention'|
        x=='Shifting'|
        x=='Visual Selective Attention'|
        x=='Tonic Attention'
      ]='Attention (b140)'
```

```
     x[x=='Auditory Memory'|
        x=='Delayed Memory'|
        x=='EF - Delayed Recall'|
        x=='Delayed Word Recall'|
        x=='DR'|
        x=='Episodic Memory'|
        x=='Episodic Verbal Memory'|
        x=='Everyday Memory Problems'|
        x=='General Long-Term Memory'|
        x=='Immediate Memory'|
        x=='Immediate Recall'|
        x=='IR'|
        x=="List Learning"|
        x=='Long Term Memory'|
        x=='Long-Term Memory'|
        x=='EF - Long-Term Memory'|
        x=='Long-Term Verbal Memory'|
        x=='Memory'|
        x=="Memory Post-Interference"|
        x=='Memory - Short Term'|
        x=='Memory Registration'|
        x=='Memory Self-Efficacy'|
        x=='Memory-Long Term'|
        x=='Memory-Long-Term Memory'|
        x=='Memory-Short Term'|
        x=='Memory-Short-Term Verbal'|
        x=='Non-Verbal Visual Memory'|
        x=='Nonverbal Memory'|
        x=='Recall'|
        x=='Recall over Time'|
        x=='Short-Term Memory'|
```

```
        x=='Short-Term Memory Recall'|
        x=='Short-Term Verbal Memory'|
        x=='Verbal'|
        x=='Verbal Declarative Memory'|
        x=='Verbal Memory'|
        x=='Verbal Short Term Memory'|
        x=='Verbal Working Memory'|
        x=='Visual Memory'|
        x=='Visual Memory [nonverbal]'|
        x=='Visual Memory [or Visuospatial Functions]'|
        x=='Visual Memory Functions'|
        x=='Visuospatial Memory'|
        x=='Visuospatial Working Memory'|
        x=='Word Memory'|
        x=='Working Memory'|
        x=='EF - Working Memory'|
        x=='Recall over Time'
      ]="Memory (b144)"
```

```
     x[x=='Adjustment to Presentation Speed'|
        x=='Attention [psychomotor Speed]'|
        x=='EF - Psychomotor Performance'|
        x=='EF - Speed'|
        x=='Hit Reaction Time'|
        x=='Motor Speed'|
        x=='Praxis-Gnosis'|
        x=='Psychomotor Speed'|
        x=='Reaction Speed'|
        x=='Reaction Time'|
        x=='Speed'
      ]='Psychomotor (b147)'
```

```
    x[x=='Gestual Praxis'|
      x=="Ideational"|
      x=='Praxia'|
      x=='Praxis'|
      x=='Planning Visuospatial Abilities'|
      x=='Visual Movement Organization'|
      x=='Visuo-Motor Coordination'|
      x=='Visuomotor Coordination'|
      x=='EF - Visuomotor Scanning'|
      x=='Visuomotor Speed'
    ]='Complex mov. seq. (b176)'
```

```
    x[x=='Gnosia'|
        x=="Functional Neglect"|
        x=='Higher Level Perception'|
        x=='Higher Visual Perception'|
        x=='Optical-Spatial Gnosis'|
```

```
        x=='Perception'|
        x=='Perceptual Functions'|
        x=='Spatial Neglect Severity'|
        x=='Spatial Perception'|
        x=='Spatial Skills'|
        x=='Unilateral Visual Neglect'|
        x=='Unilateral Visuospatial Neglect'|
        x=='Visual Inattention'|
        x=='Visual Neglect'|
        x=='Visual Perception'|
        x=='Visual Scanning'|
        x=='Visual Scanning Patterns'|
        x=='Attention Omission'|
        x=='Scanning'|
        x=='Signal Detection'|
        x=='Visual-Spatial'|
        x=='Visuo-Perceptual'|
        x=='Visuospatial'|
        x=='Visuospatial Abilities'|
        x=='Visuospatial Ability'|
        x=='Visuospatial Scanning'|
        x=='Visuospatial Cognition'|
        x=='Visuospatial Function'|
        x=='Visuospatial Functions'|
        x=='Visuospatial Perception'|
        x=='Visual Search']='Perceptual (b156)'
```

```
    x[x=='Field of Vision'|
      x=='Visual Fields']='Visual Field (b2101)'
```

```
    x[x=='Conceptual Thinking'|
        x=='Mental Control'|
        x=='Perseveration'|
        x=='Thought Operation']='Thought (b160)'
```

```
    x[x=='Abstract Reasoning'|
        x=='Abstraction'|
        x=='Cognitive Flexibility'|
        x=='Concept Formation'|
        x=='Conceptualization'|
        x=='Conflict Resolution'|
        x=='EF - Cognitive Flexibility'|
        x=='EF - Completion'|
        x=='EF'|
        x=='EF - Information Seeking'|
        x=='EF - Inhibition'|
        x=='EF - Initiation'|
        x=='EF - Mental Flexibility'|
        x=='EF - Judgment'|
```

```
        x=='EF - Planning'|
        x=='EF - Problem Solving'|
        x=='EF - Response Inhibition'|
        x=='EF - Set Shifting'|
        x=='Executive'|
        x=='Executive Functioning'|
        x=='Executive Functions'|
        x=='Executive Reasoning'|
        x=='Flexibility'|
        x=='Frontal Lobe Functions'|
        x=='Information Processing'|
        x=='Information Processing Speed'|
        x=='Initiation'|
        x=="Inhibition"|
        x=='Letter Sequencing'|
        x=='Logical Deductive Ability'|
        x=='Mental Flexibility'|
        x=="Mental Tracking"|
        x=='Mental Slowness in Relation to Daily Activities'|
        x=='Number-Letter Switching'|
        x=='Number Sequencing'|
        x=='Nonverbal Reasoning'|
        x=='Performance in Time Pressure Situations'|
        x=='Problem-Solving'|
        x=='Processing Speed'|
        x=='Processing Skills'|
        x=='Reasoning'|
        x=='Response Inhibition'|
        x=='Sequencing'|
        x=='Speed Processing'|
        x=='Time to Find Increasing Numbers'|
        x=='Self-Monitoring of Non-Motor Performance'|
        x=='Set Shifting']='HLCF (b164)'
```

```
    x[x=='Aphasia'|
        x=='Auditory Comprehension'|
        x=='Category'|
        x=='Cognitive Communicative Skills'|
        x=='Communication'|
        x=="Communication in Daily Life Situations"|
        x=='Comprehension'|
        x=='EF - Verbal Fluency'|
        x=='Expression'|
        x=='Language'|
        x=='Language - Object Naming from Line Drawing'|
        x=='Language [judged by Neuropsychologist]'|
        x=='Language Abilities'|
        x=='Language Abilities - Speech Fluency'|
        x=='Language Ability'|
        x=='Language-Auditory Comprehension'|
```

```
        x=='Language Comprehension'|
        x=='Language-Confrontation Naming'|
        x=='Language Impairment'|
        x=='Language-Phonemic Verbal Fluency'|
        x=='Language-Picture Naming'|
        x=='Language-Reading Capacity'|
        x=='Language-Sentence Comprehension'|
        x=='Information Content in Spontaneous Speech'|
        x=='Verbal'|
        x=='Verbal Ability'|
        x=='Verbal Comprehension of Complex Material'|
        x=='Verbal Expression'|
        x=='Verbal Fluency'|
        x=='Verbal Function'|
        x=='Verbal Processing'|
        x=="Verbal Comprehension"|
        x=='Letter Fluency Tasks'|
        x=='Lexical Fluency'|
        x=='Phonological Fluency'|
        x=='Semantic Fluency'|
        x=='Narrative Speech'|
        x=='Naming'|
        x=='Naming Skills'|
        x=='Word Finding'|
        x=='Word Reading'|
        x=='Word Generation'|
        x=='Word Naming'|
        x=='Written Comprehension']="Language (b167)" # language functions
under the ICF look at the expression and reception of language. In the
literature included language tests are mostly looking at higher-level
cognition because of the incorporation of different rules in addition to the
language task

    x[x=='Awareness of Visuospatial Neglect'|
        x=='Neglect'|
        x=='Personal Neglect'|
        x=='Spatial Neglect Severity'|
        x=='Unilateral Neglect'|
        x=='Unilateral Visual Neglect'|
        x=='Unilateral Visuospatial Neglect'|
        x=='Visual Neglect'|
        x=='Visuospatial Neglect'|
        x=='Extinction'|
        x=='Sensory']="Exp. of self & time (b180)"

    x[x=='Arithmetic'|
        x=='Calculation']='Calculation (b172)'

    x[x=='Alertness'|
        x=='Intrinsic Alertness'|
```

```
        x=='Vigilance']='Consciousness (b110)'


# Cognitive functions that could not be allocated to any cognitive ICF
category below

x[x=='[no Area]']='[Not Reported]'

x[x=='Commission Errors'|
        x=='Commissions [number of Times Person Responds to a Non-Target
Item]']='Error Commission'

    x[x=='Omissions'|
        x=='Item Omissions']='Omission'

    x[x=='Behavior'|
        x=='Behavioral Change'|
        x=='Behavioral Improvement']="Behavior"

    x[x=="Motor Functions"|
        x=="Motor Skills"]="Movement Functions (b750-b789)"

    x[x=='Learning'|
        x=='New Learning'|
        x=='EF - Learning'|
        x=='Verbal Learning']='Basic learning (d130-159)'

    x[x=='Premotor Functions'|
        x=='Premotor Abilities']='Premotor Function'

    x[x=='Function'|
        x=='Functional Status'|
        x=='Disability'|
        x=='Degree of Disability'|
        x=='Impairment'|
        x=='Extent of Disability']='Functional status'
```

Cognitive domains - recoding into broader semantic categories (code extract)

```
x[x=="Executive"|
    x=="Executive Functioning"|
    x=="Executive Functions"|
    x=="Executive Functions"|
    x=="Executive Reasoning"]="Executive Function"
```

```
x[x=='Alternating Attention'|
    x=='Attention'|
    x=='Attention [psychomotor Speed]'|
    x=='Attention Omission'|
    x=='Attention-Phasic Alert'|
    x=='Attentional Tasks'|
    x=='Attentiveness'|
    x=='Divided Attention'|
    x=='Focused Visual Attention'|
    x=='General Visual Attention'|
    x=='Phasic Attention'|
    x=='Selective Attention'|
    x=='Sustained Attention'|
    x=='Switching of Attention'|
    x=='Tonic Attention'|
    x=='Visual Inattention'|
    x=='Sustained'|
    x=='Selective'|
    x=='Divided'|
    x=="Spatial Attention"|
    x=='Visual Selective Attention']="Attention"
```

```
x[x=='Behavior'|
    x=='Behavioral Change'|
    x=='Behavioral Improvement']="Behavior"
```

```
x[x=='Constructing'|
    x=='Construction'|
    x=='Construction Skills'|
    x=='Constructional'|
    x=='Constructional Function'|
    x=='Constructional Praxis'|
    x=='Constructive Praxis'|
```

```
    x=='Visuospatial Construction'|
    x=='Visuoconstruction'|
    x=='Visuoconstructive Ability'|
    x=='Visuoconstuctive Functions']="Construction skills"

x[x=='Concept Formation'|
    x=='Conceptual Thinking']=
  'Conceptualization'

x[x=='Dementia'|
    x=='Dementia Diagnosis'|
    x=='Dementia Diagnostic Instrument']="Dementia"

x[x=='EF - Attention'|
    x=='EF - Cognitive Flexibility'|
    x=='EF - Completion'|
    x=='EF - Delayed Recall'|
    x=='EF - Divided Attention'|
    x=='EF'|
    x=='EF - Attention Switching'|
    x=='EF - Information Seeking'|
    x=='EF - Inhibition'|
    x=='EF - Initiation'|
    x=='EF - Learning'|
    x=='EF - Mental Flexibility'|
    x=='EF - Psychomotor Performance'|
    x=='EF - Response Inhibition'|
    x=='EF - Speed'|
    x=='EF - Verbal Fluency'|
    x=='EF - Visuomotor Scanning'|
    x=='EF - Judgment'|
    x=='EF - Long-Term Memory'|
    x=='EF - Planning'|
    x=='EF - Problem Solving'|
    x=='EF - Set Shifting'|
    x=='EF - Working Memory'|
    x=="State of Higher Mental Functions"|
    x=='Higher Cerebral Functions']="Executive Function"


x[x=='Cognition'|
    x=='Cognitive'|
    x=='Cognitive Abilities'|
    x=='Cognitive Ability'|
    x=='Cognitive Decline'|
    x=='Cognitive Disability'|
    x=='Cognitive Disorders'|
    x=='Cognitive Dysfunction'|
    x=='Cognitive Function'|
```

```
    x=='Cognitive Functioning'|
    x=='Cognitive Functions'|
    x=='Cognitive Impact Resulting from Stroke'|
    x=='Cognitive Impairment'|
    x=='Cognitive Mental Status'|
    x=='Cognitive Performance'|
    x=='Cognitive State'|
    x=='Cognitive Status'|
    x=='Degree of Cognitive Abilities'|
    x=='General Cognition'|
    x=='General Cognitive Function'|
    x=='General Cognitive Functioning'|
    x=='General Cognitive Functions'|
    x=='Global Cognition'|
    x=='Global Cognitive Assessment'|
    x=='Global Cognitive Function'|
    x=='Global Cognitive Functioning'|
    x=='Global Cognitive Functions'|
    x=='Global Cognitive Impairment'|
    x=='Global Cognitive Performance'|
    x=='Global Cognitive Status'|
    x=='Mild Cognitive Impairment'|
    x=='Moderate Cognitive Dysfunction'|
    x=='Recovery of Cognition'|
    x=='Neuropsychological Functions'|
    x=='Neuropsychological Impairment'|
    x=='Mental Status'|
    x=='Cognitive Screening'
    ]="General Cognition"


x[x=='Premorbid Ability'|
    x=='Premorbid Cognitive Function'|
    x=='Premorbid Cognitive Impairment']="Premorbid Cognitive Status"

x[x=='Premotor Abilities'|
    x=='Premotor Functions']='Premotor Function'

x[x=='Language'|
    x=='Language - Object Naming from Line Drawing'|
    x=='Language [judged by Neuropsychologist]'|
    x=='Language Abilities'|
    x=='Language Abilities - Speech Fluency'|
    x=='Language Ability'|
    x=='Language-Auditory Comprehension'|
    x=='Language Comprehension'|
    x=='Language-Confrontation Naming'|
    x=='Language Impairment'|
    x=='Language-Phonemic Verbal Fluency'|
```

```
    x=='Language-Picture Naming'|
    x=='Language-Reading Capacity'|
    x=='Language-Sentence Comprehension'|
    x=='Cognitive Communicative Skills'|
    x=='Verbal'|
    x=='Verbal Ability'|
    x=='Verbal Expression'|
    x=='Verbal Fluency'|
    x=='Verbal Function'|
    x=='Verbal Processing'|
    x=='Letter Fluency Tasks'|
    x=='Lexical Fluency'|
    x=='Phonological Fluency'|
    x=='Semantic Fluency'|
    x=='Narrative Speech'|
    x=='Naming'|
    x=='Naming Skills'|
    x=='Word Naming']="Language Skills"
```

```
x[x=='Auditory Memory'|
    x=='Delayed Memory'|
    x=='Episodic Memory'|
    x=='Episodic Verbal Memory'|
    x=='Everyday Memory Problems'|
    x=='General Long-Term Memory'|
    x=='Immediate Memory'|
    x=='Long Term Memory'|
    x=='Long-Term Memory'|
    x=='Long-Term Verbal Memory'|
    x=='Memory'|
    x=='Memory - Short Term'|
    x=='Memory Registration'|
    x=='Memory Self-Efficacy'|
    x=='Memory-Long Term'|
    x=='Memory-Long-Term Memory'|
    x=='Memory-Short Term'|
    x=='Memory-Short-Term Verbal'|
    x=='Non-Verbal Visual Memory'|
    x=='Nonverbal Memory'|
    x=='Short-Term Memory'|
    x=='Short-Term Memory Recall'|
    x=='Short-Term Verbal Memory'|
    x=='Verbal Declarative Memory'|
    x=='Verbal Memory'|
    x=='Verbal Short Term Memory'|
    x=='Verbal Working Memory'|
    x=='Visual Memory'|
    x=='Visual Memory [nonverbal]'|
    x=='Visual Memory [or Visuospatial Functions]'|
```

```
    x=='Visual Memory Functions'|
    x=='Visuospatial Memory'|
    x=='Word Memory'|
    x=='Working Memory'|
    x=='Verbal'|
    x=="Visuospatial Working Memory"]=
  "Memory"

x[x=='Awareness of Visuospatial Neglect'|
    x=='Neglect'|
    x=='Personal Neglect'|
    x=='Spatial Neglect Severity'|
    x=='Unilateral Neglect'|
    x=='Unilateral Visual Neglect'|
    x=='Unilateral Visuospatial Neglect'|
    x=='Visual Neglect'|
    x=='Sensory']="Neglect"

x[x=='Neurologic Function'|
    x=='Neurological Function']="Neurologic Function"



x[x=='Abstract Reasoning'|
    x=='Nonverbal Reasoning'|
    x=='Reasoning']="Reasoning Skills"

x[x=='Learning'|
    x=='New Learning'|
    x=='Verbal Learning']="Learning Skills"

x[x=='Auditory Comprehension'|
    x=='Comprehension'|
    x=='Verbal Comprehension of Complex Material'|
    x=='Written Comprehension']="Comprehension Skills"

# x[x=='Naming'|
#     x=='Naming Skills'|
#     x=='Word Naming']='Naming Skills'## merged with language skills



x[x=='Higher Visual Perception'|
    x=='Planning Visuospatial Abilities'|
    x=='Visual Fields'|
    x=='Visual Movement Organization'|
    x=='Visual Perception'|
    x=='Visual Scanning'|
```

```
    x=='Visual Scanning Patterns'|
    x=='Visual-Spatial'|
    x=='Visuo-Motor Coordination'|
    x=='Visuo-Perceptual'|
    x=='Visuomotor Coordination'|
    x=='Visuomotor Speed'|
    x=='Visuospatial'|
    x=='Visuospatial Abilities'|
    x=='Visuospatial Ability'|
    x=='Visuospatial Cognition'|
    x=='Visuospatial Function'|
    x=='Visuospatial Functions'|
    x=='Visuospatial Scanning'|
    x=='Visual Search'|
    x=='Visuospatial Perception']="Visual Skills"

x[x=='Information Processing'|
    x=='Information Processing Speed'|
    x=='Processing Speed']="Processing Skills"


x[x=='Orientation'|
    x=='Orientation to Time'|
    x=='Temporal Orientation'|
    x=='Orientation to Time & Space']="Orientation Skills"


x[x=='Social Acuity'|
    x=='Social Cognition'|
    x=='Social Interaction']="Social Cognitive Skills"

x[x=='Delayed Word Recall'|
    x=='Immediate Recall'|
    x=='IR'|
    x=='DR'|
    x=='Recall'|
    x=='Recall over Time']="Recall"

x[x=='Higher Level Perception'|
    x=='Perception'|
    x=='Perceptual Functions'|
    x=='Spatial Perception']='Perception'

x[x=='Gestual Praxis'|
    x=='Praxia'|
    x=='Praxis'|
    x=='Praxis-Gnosis'|
    x=="Ideational"]="Praxis"
```

```
x[x=='Motor Functions'|
    x=='Motor Skills'|
    x=='Motor Speed']='Motor Skills'

x[x=='General Inteligence'|
    x=='General Intellectual Functioning'|
    x=='General Intelligence'|
    x=='Intellectual Abilities'|
    x=='Intellectual Ability']='Intellectual Abilities'

x[x=='Hit Reaction Time'|
    x=='Reaction Speed'|
    x=='Reaction Time']='Reaction Time'

x[x=='Function'|
    x=='Functional Status'|
    x=='Disability'|
    x=='Degree of Disability'|
    x=='Extent of Disability']='Functional Status'

x[x=='Cognitive Flexibility'|
    x=='Mental Flexibility']='Cognitive Flexibility'


x[x=='[no Area]']='[Not Reported]'
```

Cognitive domains grouped into categories

	Cognitive domain	Times used
1	General Cognition	174
2	Memory	110
3	Executive Function	87
4	Attention	63
5	Language Skills	55
6	Visual Skills	34
7	Any [Not Reported]	18
8	Construction skills	17
9	Neglect	15
10	Processing Skills	13
11	Orientation Skills	10
12	Learning Skills	9
13	Functional Status	8
14	Reasoning Skills	8
15	Social Cognitive Skills	8
16	Comprehension Skills	7
17	Communication	6
18	Praxis	6
19	Recall	6
20	Dementia	5
21	Intellectual Abilities	5
22	Perception	5
23	Speed	5
24	Cognitive Flexibility	4
25	Concentration	4
26	Motor Skills	4
27	Psychomotor Speed	4
28	Conceptualization	3
29	Premorbid Cognitive Status	3
30	Reaction Time	3
31	Abstraction	2
32	Alertness	2
33	Behavior	2
34	Expression	2
35	Gnosia	2
36	Logical Deductive Ability	2
37	Neurologic Function	2
38	Perseveration	2
39	Premotor Function	2
40	Problem-Solving	2
41	Vigilance	2
42	Adjustment to Presentation Speed	1
43	Aphasia	1
44	Arithmetic	1
45	Calculation	1
46	Category	1
47	Cognitive Impairment	1
48	Commission Errors	1
49	Commissions [number of Times Person Responds to a Non-Target Item]	1
50	Communication in Daily Life Situations	1
51	Conflict Resolution	1
52	Disengagement Time	1
53	Extinction	1
54	Field of Vision	1
55	Flexibility	1
56	Frontal Lobe Functions	1
57	Functional Neglect	1
58	Impairment	1
59	Information Content in Spontaneous Speech	1
60	Inhibition	1
61	Initiation	1
62	Intrinsic Alertness	1
63	Item Omissions	1
64	Letter Sequencing	1
65	List Learning	1
66	Memory Post-Interference	1
67	Mental Control	1
68	Mental Slowness in Relation to Daily Activities	1
69	Mental Tracking	1

70	Music Cognition	1
71	Nonverbal Directed Fluency Task	1
72	Number Sequencing	1
73	Number-Letter Switching	1
74	Optical-Spatial Gnosis	1
75	Outcome of Rehabilitation	1
76	Performance in Time Pressure Situations	1
77	Pre-Morbid Intellectual Functioning	1
78	Repetition	1
79	Response Inhibition	1
80	Scanning	1
81	Self-Monitoring of Non-Motor Performance	1
82	Sequencing	1
83	Set Shifting	1
84	Shifting	1
85	Signal Detection	1
86	Spatial Skills	1
87	Speed Processing	1
88	Therapeutic Efficacy	1
89	Thought Operation	1
90	Time to Find Increasing Numbers	1
91	Verbal Comprehension	1
92	Visuospatial Neglect	1
93	Word Finding	1
94	Word Generation	1
95	Word Reading	1

Instrument categorization (code extract)

```
b[b=="1-Min Animal Naming Test" |
      b=="1-Min Semantic Fluency Tests with Animals" |
      b=="Animal Fluency Test" |
      b=="Animal Naming Fluency Test" |
      b=="Animal Naming Test" |
      b=="Animals"]=
    "Fluency Test [animals]"

  b[b=="15-Item BNT"|
      b=="15-Item Subset of the BNT"]=
    "BNT [15-item version]"

  b[b=="60-Item BNT"]=
    "BNT [60-item version]"

  b[b=="10 Word List Learning"|
      b=="10-Word List"|
      b=="10-Word List Learning Task"|
      b=='Learning a Series of 10 Unrelated Words'|
      b=="10-Word Memory Test"|
      b=="10-Word Test"]=
    "10-Word List Learn"

  b[b=="5-Word Repetition"|
      b=="5-Word Repetition with 3 Min Delay"]=
    "5-Word Rep"
  b[b=='ACE-R']='ACER'

  b[b=="ADT"]="Auditory Detection"

  b[b=="Aphasia Scale [from ADAS-Cog]"]=
    "ADAS-Cog [aphasia Scale]"

  b[b=="Orientation [from ADAS-Cog]"]=
    "ADAS-Cog [orientation]"

  b[b=="Arithmetic"|
      b=="Arithmetic [from WAIS-R]"|
      b=="Arithmetic Problem Solving"]=
    "Arithmetic"

  b[b=="Baking Tray Task"]=
    "Baking Tray Test"

  b[b=="Calculation"|
```

```
      b=="Calculation"|
      b=="Calculation Test"]=
    "Calculation Test"

  b[b=='DCT'|
      b=='Digit Cancellation']=
    'Digit Cancel Task'

  b[b=="Category"|
      b=="Category Fluency"]=
    "Fluency Test [categories]"

  b[b=="Categorical Verbal Fluency [animals-1-Min]"|
      b=="Category Fluency [animal Naming]"|
      b=="Category Fluency [animals]"|
      b=="Category of Animals"]=
    "Fluency Test [animals]"

  b[b=="Category Fluency [animals & Food Subtasks]"|
      b=="Category Naming [fruits-Vegetables & Fish]"]=
    "Fluency Test [categories]"

  b[b=="CBS"|
      b=="CBT"]=
    "Corsi Blocks Test"

  b[grep('CNT ',b)]='CNT battery [vcpt + acpt+ dsf & b + vsf & b + avlt &
vrt]'


  b[b=="CDT"|
      b=="Clock Drawing"|
      b=="Clock Drawing"|
      b=="Clock Task"|
      b=="Clock-Drawing"|
      b=="Clock Drawing [from BLAD]"]=
    "Clock Drawing Test"

  b[b=="CNS"]=
    "CNS-Vital Signs Test"


  b[b=="BIT-Conventional Subtest"]=
    "BIT [Conventional Subtest]"

  b[b=="Copying Task [from BIT]"]=
    "BIT [Copying Task]"
```

```
b[b=="Picture Scanning [from BIT]"]=
  "BIT [Picture Scanning]"

b[b=="Star Cancellation [from BIT]"]=
  "BIT [Star Cancellation]"

b[b=="Copy Cube"]=
  "Copy a Cube"
b[b=="Construction Ability"|
    b=="Constructional Ability"]=
  'Construction Abilities'


b[b=="CP"]=
  "Clock Perception"

b[b=="Depression Anxiety Stress Scale"]=
  "DASS"


b[b=="Delayed Recall [from RAVLT]"|
    b=="Delayed Recall Test"|
    b=="DR"|
    b=="DR [from WMS-III]"]=
  "DR"

b[b=="DS [from WAIS - III]"|
    b=="DS [from WAIS-II]"|
    b=="DS [from WAIS-III]"|
    b=="DS [from WAIS-IV]"|
    b=="DS [from WAIS-R]"|
    b=="DS [from WAIS]"|
    b=="DS [from WMS-R]"|
    b=="DS"]=
  "Digit sp"

b[b=='DSB'|
  b=="DSB [from WAIS-III-R]"|
    b=="DSB [from WAIS-III]"|
    b=="DSB [from WAIS - III]"]=
  "Digit sp-b"

b[b=="DSB & DSF" |
    b=="DSB & DSF [from WMS-III]"|
    b=="DSF & DSB" |
    b=="DSF & DSB [from WMS]"
  ]=
  "Digit sp-f & b"
```

```
b[b=="DSF"|
    b=="DSF [from WAIS-III]"|
    b=="DSF [from WAIS]"|
    b=="DSF [wais-III-R]"]=
  "Digit sp-f"

b[b=="Go-No-Go Task"|
    b=="Go-No-Go Test"|
    b=="Go-No-Go Test [initiation & Response-Inhibition]"]=
  "Go-No-Go Test"

b[b=="GP"|
    b=="GPT"]=
  "Grooved Peg Test"

b[b=="I-Flex"|
    b=="I-Flex [a Short Form of the EXIT]"]=
  "I-Flex"

b[b=="IQCODE-SF"|
    b=="IQCODE-SS"]=
  "IQ-CODE"

b[b=="JLO"|
    b=="JLO [sf]"]=
  "Judg Line Orient"

b[b=="LCT"|
    b=="LCT - For Right Hemisphere Stroke"|
    b=="Letter Cancellation"|
    b=="Letter Cancellation Task"|
    b=="Single LCT"]=
  "Letter Cancellation"

b[b=="1-Min Phonemic Verbal Fluency [letters F-a-S]"|
    b=="1-Minute Word Naming Trial [letters C-F-L]"|
    b=="Letter Fluency"|
    b=="Letter Fluency [words with P]"|
    b=="Letters F-a-S"]=
  "Fluency Test [Letters]"

b[b=="LM [from WMS-III]"|
    b=="LM [from WMT-R]"|
    b=="LM I & LM II [from WMS-R]"|
    b=="LM-I & LM-II [from WMS-R]"|
    b=="LM-I & LM-II"|
    b=='LM'|
    b=='WLM']=
```

```
    "Log Mem"

  b[b=="LM [dr]"|
      b=="LM II [from WMS-R]"]=
    "Log Mem II"

  b[b=="LM I [from WMS-R]"]=
    "Log Mem I"

  b[b=="Construction of the MMSE"]=
    "MMSE [Construction Item]"

  b[b=="Memorize Three Words [from MMSE]"]=
    "MMSE [Three-Word Memory Item]"

  b[b=="Orientation Items of the MMSE"]=
    "MMSE [Orientation Items]"

  b[b=="Spatial Orientation [from MMSE]"]=
    "MMSE [Spatial Orientation Item]"

  b[b=="MMSE [acute & Chronic Stages Only]"|
      b=="MMSE [bengali Version]"|
      b=="MMSE [brief Version]"|
      b=="MMSE [chinese Version]"|
      b=="MMSE [italian Telephone Version]"|
      b=="MMSE [korean Version]"|
      b=="MMSE [thai Version]"]=
    "MMSE"

  b[b=="Modified BVRT"]=
    "BVRT [modified Version]"

  b[b=="or TMT a"]=
    "TMT A"

  b[b=="Phonemic Fluency"|
      b=="Phonemic Fluency [letter S]"|
      b=="Phonemic Fluency [letters F-a-S]"|
      b=="Phonemic Fluency Task"|
      b=="Phonemic Test"|
      b=="Phonetic Fluency"]=
    "Fluency Test [phonemic]"

  b[b=="Semantic Fluency"]=
    "Fluency Test [semantic]"

  b[b=="Verbal Fluency"|
```

```
      b=="Verbal Fluency [animal & Food Categories]"|
      b=="Verbal Fluency [animals]"|
      b=="Verbal Fluency [animals & Professions]"|
      b=="Verbal Fluency [animals] [from DAS]"|
      b=="Verbal Fluency [letters F-a-S & B-H-R]"|
      b=="Verbal Fluency Test"|
      b=="VF - Categorical [animals]" |
      b=="VF - Phonological [words]" |
      b=="VFT"|
      b=="VFT [generation]"|
      b=="Word Fluency"|
      b=="Word Fluency [phonemic & Semantic]"]=
    "Fluency Test [verbal]"

  b[b=="Picture Completion" |
      b=="Picture Completion [from WAIS-R]"]=
    "Picture Completion"

  b[b=="R-CAMCOG"]=
    "CAMCOG"

  b[b=="RAPM [sf]"]=
    "RAPM"

  b[b=="WCFST"|
      b=="Weigl CST"|
      b=="Weigl Sorting Test"]=
    "Weigl Color Sorting Test"



  ######CLEANING FURTHER (REMOVING SUBTEST NAMES)

b[b=='ADAS [four Pictures]'|
    b=='ADAS [recall of Four Pictures]'|
    b=='ADAS-Cog'|
    b=='ADAS-Cog [aphasia Scale]'|
    b=='ADAS-Cog [orientation]']='ADAS-Cog'


b[b=="BDT"|
    b=="BDT [from WAIS-II]"|
    b=="BDT [from WAIS-III]"|
    b=="BDT [from WAIS-R]"|
    b=="BDT [from WAIS]"]=
  "Block Design Test"

b[b=='BIT'|
```

```
    b=='BIT [Conventional Subtest]'|
    b=='BIT [Copying Task]'|
    b=='BIT [Picture Scanning]'|
    b=='BIT [Star Cancellation]'|
    b=="BIT-Reading"|
    b=="BIT-Star"]='BIT'


b[b=="BNT"|
    b=="BNT [15-item version]"|
    b=="BNT [60-item version]"|
    b=="BNT [french Version]" |
    b=="BNT [modified Version]"|
    b=="BNT [sf]"]=
  "Boston Naming Test"

b[b=='BVRT [modified Version]'|
    b=='BVRT [sf]'|
    b=='BVRT-SF']='BVRT'

b[b=="CASI [chinese Version]"]=
  'CASI'

b[b=='CAMCOG'|
    b=='CAMCOG-R [section B Only]']='CAMCOG'


b[b=='Copying 4 Geometric Figures'|
    b=='Copying Designs'|
    b=='Copying Simple Figures'|
    b=='Figure Copying [from CERAD]'|
    b=='Time of Copying Task']='Figure Copying'

b[b=="COWAT [total Number of Words]"]=
  "COWAT"

b[b=='CVLT'|
    b=='CVLT-II']='CVLT'


b[b=='Digit Symbol'|
    b=="Digit-Symbol"|
    b=='Digit Symbol [coding Subtest from WAIS-III]'|
    b=='Digit Symbol [from WAIS-II]'|
    b=="DST"|
    b=='Digit Symbol [from WAIS]'|
    b=='DSS [from WAIS-III]'|
    b=="DSST"]='Digit Symbol'
```

```
b[b=="Digit sp"|
    b=="Digit sp-b" |
    b=='Digit sp-f & b'|
    b=="Digit sp-f"]="Digit sp"


b[b=='FCSRT [delayed Free Recall]'|
    b=='FCSRT [total of the 3 Free Recalls Trials]'|
    b=='Immediate Recall [from FACSRT]'|
    b=='FACSRT']=
  'FCSRT'

b[b=="Fluency Test [animals]"|
    b=="Fluency Test [categories]"|
    b=="1-Min Fluency Test [animals & Professions]"|
    b=="Category Fluency [animals & Professions]"|
    b=="Fluency Test [Letters]"|
    b=="Fluency Test [phonemic]"|
    b=="Fluency Test [semantic]"|
    b=="Fluency Test [verbal]"]=
  "Verbal Fluency"

b[b=="Geometric Figures [dr]" |
  b=="Geometric Figures [ir]" ]=
  "Geometric Figures"

b[b=='IQ-CODE'|
    b=='IQCODE']='IQ-CODE'

b[b=="Ideomotor Apraxia Subtest Items"]=
  "Ideomotor Apraxia Test"

b[b=="KSNAP [four Letter Word Subtest]"|
    b=="KSNAP [gestalt Closure Subtest]"|
    b=="KSNAP [mental Status Subtest]"]=
  'KSNAP'

b[b=="Letter-Number Sequencing [from WAIS-III]"]=
    "Letter-Number Sequencing"

b[b=="Log Mem" |
    b=="Log Mem I"|
    b=="Log Mem II"|
    b=="Delayed Logic Memory"|
    b=="LM I & LM II"]=
  "Log Mem"
```

```
b[b=="Luria's Sequences"|
  b=="Lurias Premotor Sequences" ]=
  "Luria's sequences"

b[b=="Matrix Reasoning [from WAIS-III]"|
  b=="Matrix Reasoning [from WAIS]"|
  b=="Matrix Reasoning [from WASI]"]=
  "Matrix Reasoning"

b[b=='MDRS'|
    b=='MDRS [initiation-Perseveration]'|
    b=='Perseveration [from MDRS]'|
    b=='Subtests of the MDRS']="MDRS"

b[b=="Mental Control [from WMS-III]" |
    b=="Mental Control [from WMS]"]=
  "Mental Control"

b[b=='MMSE'|
    b=='MMSE [Construction Item]'|
    b=='MMSE [Orientation Items]'|
    b=="MMSE [modified Version]"|
    b=="MMSE [modified]"|
    b=='MMSE [Spatial Orientation Item]'|
    b=="Temporal & Spatial Orientation [from MMSE]"|
    b=='MMSE [Three-Word Memory Item]']=
  'MMSE'

b[b=='MoCA'|
    b=='MoCA [attention Subtest]'|
    b=='Rythms Subtest [from MoCA]']='MoCA'

b[b=="Naming"|
    b=="Naming [from CERAD]"|
    b=="Naming 5 Objects"|
    b=="Naming 5 Parts of Object"|
    b=="Naming of Pictures of Objects"|
    b=="Naming [categories]"]=
  "Naming"

b[b=='PASAT'|
    b=='PASAT [2 Slowest Trials]']=
  'PASAT'

b[b=="RAVLT"|
    b=="RAVLT [dr]"|
    b=="RAVLT [ir-DR-DRec]"|
    b=="RAVLT Total Number of Learnt Words from A1-A5"|
```

```
    b=="RAVLT [trial 6]"|
    b=="RAVLT [trial 7 - 20-Min Delay]"|
    b=="DRec [from RAVLT]"|
    b=="RAVLT [trials 1-5]"]=
  'RAVLT'

b[b=="RCPM"|
  b=="RCPM [set A]"]=
  'RCPM'

b[b=="ROCF"|
    b=="ROCF [copy Score]"|
    b=='ROCFT'|
    b=='RCFT [immediate & delayed Recall]'|
    b=='RCFT [recall Score]'|
    b=='RCFC'|
    b=="ROCF [delay Score]"]=
  'ROCF'

b[b=='RBANS'|
    b=='RBANS [naming & Coding Subtests]'|
    b=='Recognition Memory Test [from RBANS]']=
  'RBANS'


b[b=="Similarities [from WAIS-III]"|
    b=="Similarities Subtest [from WAIS-II]"|
    b=="Similarities Subtests [from WAIS-R]"|
    b=="Similarity Test"]=
  "Similarities"

b[b=="SIS"|
    b=="SIS Memory"]=
  'SIS'

b[b=="Spontaneous Speech"]=
  "Spontaneous Speech Fluency"

b[b=='Simple Reaction Time']=
  'SRT'

b[b=="Stroop  [modified] [set B Minus A]"|
    b=="Stroop [color Naming]"|
    b=="Stroop [interference Minus Naming]"|
    b=="Stroop [interference Score]"|
    b=="Stroop [interference Subtest]"|
    b=="Stroop [modified Version]" |
    b=="Stroop [scwt Parts 1 & 2]"|
    b=="Stroop [victoria Version]"|
```

```
    b=="Stroop Animal Test"|
    b=="Stroop Test 1 & 2 [victoria Version]"|
    b=="Stroop-CWIT [from the D-KEFS]"|
    b=="Stroop [modified] [set B Minus A]"]=
  "Stroop"

b[grep('Two Computerized Attention',b)]=
  "Attention Subtests [from TAPr]"

b[b=="SR"|
  b=="Story Recall [from WMS]"|
  b=="SR [ir & DR]"|
  b=="Story [dr]"|
  b=="Story [ir]"|
  b=="Story Recall [from RBMT]"|
  b=="Story Recall [ir & DR]"]=
  'Story Recall'

b[b=='Symbol Search [from WAIS]']=
  'Symbol Search'

b[b=='Attention Subtests [from TAPr]']=
  'TAPr'


b[b=="TMT [interference Score]"|
    b=="TMT [set B Minus A]"|
    b=="TMT [set-Shifting]"|
    b=="TMT [sets A1-A2 & B]"|
    b=="TMT a"|
    b=="TMT A"|
    b=="TMT a & B"|
    b=="TMT B"]=
  "TMT"
b[b=="Token Test [modified Version-Time to Complete]"|
    b=="Token Test [sf]"|
    b=='Token Test [22-Item-Sf]']=
  "Token Test"

b[b=="Vocabulary [from WAIS]"|
    b=="Vocabulary Subtest [from WAIS-R]"]=
  "Vocabulary [from WAIS]"

b[b=="VR"|
    b=="VR [copy Score] [from WMS-R]"|
    b=="VR [from WMS-III]"|
    b=="VR [from WMS]"|
    b=="VR [i - II & DRec] [from WMS-III]"|
    b=="VR [ir-DR & DRec - From WMS-R]"|
```

```
    b=="VR [ir-DR & DRec] [from WMS-R]"|
    b=="VR Copy [from WMS-III]"|
    b=="VR Copy Task [from WMS-R]"|
    b=="VR I & VR II [from WMS-R]"]=
  "Visual Reproduct"

b[b=="VOSP"|
    b=="VOSPB"|
    b=="VOSP [silhouettes Subtest]"]=
  'VOSP'

b[b=="WAB"|
  b=="WAB [finger Gnosis & Stereognosis]"]=
  'WAB'

b[b=="WCST [modified Version]"|
    b=="WCST [nelson Version]"|
    b=="WCST"]=
  "Wisconsin Card Sort"


b[b=="Writing Task"]=
  "Writing"

b[b=="Word List Memory [or Flash Memory]"|
    b=="Word List Learn"|
    b=="Word List Memory [encoding]"]=
  "Word List Mem"

b[b=="WLR [ir-DR & DRec]"|
    b=="WLR [ir-DR & DRec]"|
    b=="WLR [ir-DR & DRec]" |
    b=="Word List Recall [or Learning Period]"|
    b=="Word List Recognition [or Recall of Knowledge]"]=
  "Word List Recall & Recog"

b[b=="WLL [from CERAD]"|
    b=="WLL [ir & DR from CERAD]"|
    b=="WLL"|
    b=="Word List Recall & Recog"]=
  "Word List Learn"
```
